# Eco-Friendly Synthesis of a New Class of Pyridinium-Based Ionic Liquids with Attractive Antimicrobial Activity

**DOI:** 10.3390/molecules200814936

**Published:** 2015-08-14

**Authors:** Mouslim Messali

**Affiliations:** Department of Chemistry, Taibah University, Al-Madina Al-Mounawara 30002, Saudi Arabia; E-Mail: mouslim@mail.be; Tel.: +966-562-441-572

**Keywords:** eco-friendly synthesis, ultrasound irradiation, ionic liquid, antimicrobial activity

## Abstract

The present study reports a green synthesis of a new family of ionic liquids (ILs) based on functionalized 4-dimethylaminopyridinium derivatives. The structures of 23 newly synthesized ILs (**2**–**24**) were confirmed by FT-IR, ^1^H-, ^13^C-, ^11^B-, ^19^F-, and ^31^P-NMR spectroscopy and mass spectrometry. The antimicrobial activity of all novel ILs was tested against a panel of bacteria and fungi. The results prove that all tested ILs are effective antibacterial and antifungal agents, especially 4-(dimethylamino)-1-(4-phenoxybutyl)pyridinium derivatives **5** and **19**.

## 1. Introduction 

Ionic liquids (ILs) have received increased attention in recent years due to their outstanding and unique properties, such as negligible vapor pressure, non-volatility, non-flammability, excellent thermal stability, and high electrical conductivity [1,2,3,4,5,6,7]. Generally, ILs are defined as organic salts with a melting point below 100 °C that contain an organic cation combined with various anions, such as halides or fluorinated anions [8]. An extensive range of applications of ILs has been reported based on the above-cited characteristics. For example, as an alternative solvent of volatile organic compounds [9,10], as media for the electrodeposition of metals [11], catalysts and biocatalysts [12,13,14], potential corrosion inhibitors [15,16], and in food chemical science [17]. Additionally, the antimicrobial activity of various families of ILs against both environmental and clinically important microorganisms has been studied by different research groups [18,19].

In our previous research, we investigated green procedures, including microwave and ultrasound irradiation, to provide a clean synthesis of ILs compared with their conventional preparation. The reduction in reaction times and the increase in the product yields were the most important advantages from using these eco-friendly technologies [20,21].

Continuing our interest in the design and synthesis of potential antimicrobial agents based on ionic liquids [22,23], we herein present an interesting preparation of a new series of ILs based on 4-(dimethylamino)pyridinium derivatives. All newly-synthesized ILs were screened for their antibacterial and antifungal activity against eight pathogenic strains.

## 2. Results and Discussion

### 2.1. Chemistry

**Scheme 1:**
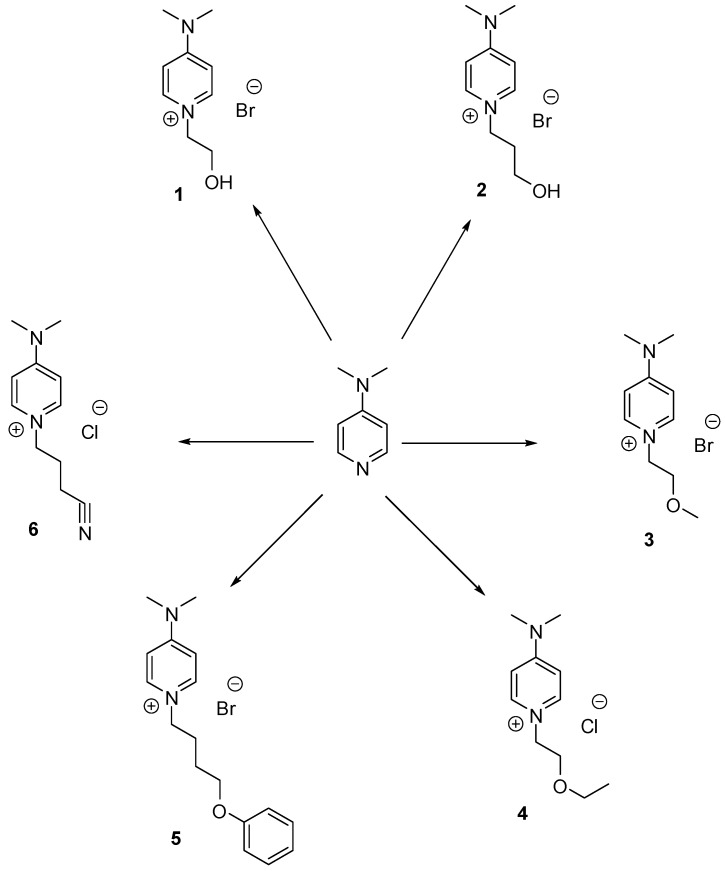
*N*-alkylation of 4-dimethylaminopyridine under ultrasonic irradiation conditions. RX/toluene, 80 °C, 5 h. R = –(CH_2_)_2_OH for **1**; –(CH_2_)_3_OH for **2**; –(CH_2_)_2_OCH_3_ for **3**; –(CH_2_)_2_OCH_2_CH_3_ for **4**; –(CH_2_)_4_OPh for **5**; –(CH_2_)_3_CN for **6**; X = Cl, Br.

**Scheme 2:**
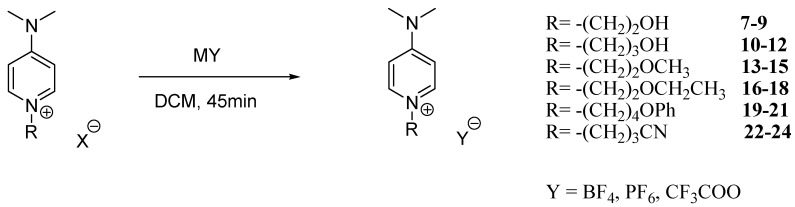
Anion metathesis under ultrasonic irradiation conditions (US): MY, dichloromethane, 70 °C, 45 min. M = Na, K.

ILs **2**–**24** were synthesized under ultrasound irradiation, as shown in Scheme 1 and Scheme 2.

To the best of our knowledge, all are novel ILs except 4-(dimethylamino)-1-(2-hydroxyethyl)pyridinium bromide **1** [24]. Initially, the nucleophilic alkylation of 4-dimethylaminopyridine (DMAP) with various functionalized alkyl halides in toluene was carried out under ultrasound irradiation for 5 h at 80 °C, and afforded the desired ILs **1**–**6** in 79%–85% yield as solids (Table 1).

**Table 1:** Alkylation and anion metathesis using ultrasound irradiation.

Ionic Liquid	RX	Yield (%) for the *N*-Alkylation ^a^	MY	Yield (%) for the Anion Metathesis ^b^
**1**	HO(CH_2_)_2_Br	82		
**7**			NaBF_4_	97
**8**			KPF_6_	98
**9**			NaOOCCF_3_	96
**2**	HO(CH_2_)_3_Br	85		
**10**			NaBF_4_	97
**11**			KPF_6_	99
**12**			NaOOCCF_3_	97
**3**	CH_3_O(CH_2_)_2_Br	81		
**13**			NaBF4	94
**14**			KPF6	92
**15**			NaOOCCF3	94
**4**	CH_3_CH_2_O(CH_2_)_2_Cl	79		94
**16**			NaBF_4_	93
**17**			KPF_6_	95
**18**			NaOOCCF_3_	92
**5**	PhO(CH_2_)_4_Br	83		93
**19**			NaBF_4_	94
**20**			KPF_6_	94
**21**			NaOOCCF_3_	93
**6**	NC(CH_2_)_3_Cl	78		
**22**			NaBF_4_	92
**23**			KPF_6_	93
**24**			NaOOCCF_3_	92

^a^ Time (5 h), temperature (80 °C) in toluene; ^b^ Time (45 min), temperature (70 °C) in dichloromethane.

In the second step, three fluorine-containing anions were introduced to obtain low melting point ILs. This metathesis reaction consisted of a halide anion exchange using sodium tetrafluoroborate, potassium hexafluorophosphate or sodium trifluoroacetate under ultrasonic irradiation (Scheme 2).

The desired ionic liquids **7**–**24** were synthesized by reacting the mixture of 4-dimethylaminopyridinium ILs **1**–**6** and different metal salts in a closed vessel exposed to ultrasound irradiation for 45 min at 70 °C. The excellent yields for this step are summarized in Table 1.

The structures of ILs **1**–**6** were confirmed by ^1^H-NMR, ^13^C-NMR, FT-IR, and LCMS. The ^1^H-NMR spectrum contained a singlet around δ_H_ 3.20 ppm corresponding to the six protons for N(CH_3_)_2_. The protons of the different methylene groups (CH_2_) of all the ILs were observed at their usual chemical shifts. In addition, the signals of the pyridinium protons appeared as two doublets around δ_H_ 7 and 8 ppm. For IL **5**, more aromatic protons for the phenyl group were observed as a multiplet at δ_H_ 6.89–6.93 ppm. It is also important to note the disappearance of the singlet around δ_H_ 5.1 ppm for the OH proton in the spectra of ILs **1** and **2**, as the NMR solvent was D_2_O.

All of the ^13^C-NMR spectra of ILs **1**–**6** showed the CH_2_ and CH_3_ signals at their usual chemical shifts. For example, the signals for the N(CH_3_)_2_, (OCH_2_), and (NCH_2_) carbons of IL **2** appeared at δ_C_ 39.6, 54.6, and 57.9 ppm, respectively. Furthermore, the aromatic carbons and the C=N gave signals between δ_C_ 107–158 ppm.

The IR spectra of ILs **1** and **2** showed a major absorption band at 3213 cm^−1^, indicating the presence of hydroxyl group (OH). In addition, the FT-IR spectra of ILs **1**–**5** contained peaks around 1160 cm^−1^, which is consistent with the presence of a C–O bond belonging to an ether or hydroxyl group. To support the NMR evidence, the band at 2247 cm^−1^ (characteristic of a cyano group), clearly confirms the structure of IL **6**.

The structures of ILs **7**–**24** were also fully characterized. The ^1^H- and ^13^C-NMR spectra were essentially the same as those recorded for the parent ILs **1**–**6**, and the ^11^B-, ^19^F-, and ^31^P-NMR were also recorded to confirm the success of the metathesis reactions for these compounds. All peaks related to B or F in BF_4_ appeared around δ_B_ −1 ppm and δ_F_ −148 ppm. The ^31^P-NMR and ^19^F-NMR spectra contained a septuplet at δ_P_ −131 to −157 ppm related to ***P***F_6_, and a doublet around δ_F_ −69 to −71 ppm related to P***F***_6_. Finally, the presence of CF_3_COO was also confirmed by the ^19^F NMR, and gave a peak around δ_F_ −73 ppm.

### 2.2. Antimicrobial Activity

As mentioned, one of the aims of the current work was to test the antibacterial and antifungal activities of all newly-synthesized ILs. ILs **1**–**24** were screened *in vitro* for their antibacterial activity against a panel of bacteria and fungi. These were two Gram-positive bacteria (*Streptococcus pneumonia* and *Bacillus subtilis*) and two Gram-negative bacteria (*Pseudomonas aeruginosa* and *Escherichia coli*) using an agar diffusion method with Mueller-Hinton agar medium for the bacteria [25]. The ILs **1**–**24** were also screened against four fungal strains (*Aspergillus fumigates*, *Syncephalastrum* *racemosum*, *Geotrichum* *candidum*, and *Candida albicans*) using an agar diffusion method with Sabouraud’s agar medium for the fungi [26].

The mean values for inhibition zone diameter summarized in Table 2 show that, except IL **4**, **7**–**10** and **22**–**24**, which did not show any antimicrobial activity against all the tested bacterial and fungal strains, all ILs displayed good to excellent antibacterial activities against the growth of all selected bacteria compared with the standards Amphotericin B, ampicillin and Gentamicin.

**Table 2:** Antimicrobial activities (inhibition zone; diameter in mm) of ILs **1**–**24** against four fungi and four bacteria.

Compd	Antifungal Activity				Antibacterial Activity			
	*A. fumigatus*	*S. racemosum*	*G. candidum*	*C. albicans*	*S. pneumoniae*	*B. subtilis*	*P. aeruginosa*	*E. coli*
**1**	11.9	13.2	14.4	11.6	11.2	13.6	15.8	16.2
**2**	19.3	20.4	17.7	15.6	16.5	18.7	15.2	18.4
**3**	12.2	13.1	13.9	10.8	11.6	12.3	10.1	10.9
**4**	NA	NA	NA	NA	NA	NA	NA	NA
**5**	23.4	22.3	28.1	26.3	23.2	27.4	22.3	23.2
**6**	11.1	12.1	12.9	10.3	12.4	12.9	10.2	11.9
**7**	NA	NA	NA	NA	NA	NA	NA	NA
**8**	NA	NA	NA	NA	NA	NA	NA	NA
**9**	NA	NA	NA	NA	NA	NA	NA	NA
**10**	NA	NA	NA	NA	NA	NA	NA	NA
**11**	14.3	15.9	17.3	13.1	18.3	20.1	14.6	16.2
**12**	21.3	20.2	22.1	19.6	20.4	21.3	17.3	20.6
**13**	18.3	19.6	18.2	17.3	18.1	19.6	15.2	17.3
**14**	16.3	17.8	19.2	16.7	17.4	18.3	17.2	20.9
**15**	NA	NA	NA	NA	NA	NA	NA	NA
**16**	18.3	19.3	21.2	15.7	18.4	19.2	16.7	17.3
**17**	16.3	18.2	19.4	14.6	17.3	18.2	15.3	14.6
**18**	18.2	19.6	15.6	13.9	14.5	16.2	13.3	16.5
**19**	24.2	23.3	29.2	27.3	24.6	28.3	24.2	24.9
**20**	16.3	18.2	20.1	14.3	19.3	20.4	16.2	20.3
**21**	15.3	16.9	19.2	13.4	18.2	16.8	15.9	17.5
**22**	NA	NA	NA	NA	NA	NA	NA	NA
**23**	NA	NA	NA	NA	NA	NA	NA	NA
**24**	NA	NA	NA	NA	NA	NA	NA	NA
**Amphotericin B**	20.4	17.3	26.3	22.0	---	---	---	---
**Ampicillin**	---	---	---	---	20.8	26.7	---	---
**Gentamicin**	---	---	---	---	---	---	16.1	18.3

The results also clearly reveal that *S. racemosum* and *P. aeruginosa* are susceptible to the action of all tested ILs. Furthermore, IL **5** (4-(dimethylamino)-1-(4-phenoxybutyl)pyridinium bromide) and IL **19** (4-(dimethylamino)-1-(4-phenoxybutyl)pyridinium tetrafluoroborate) exhibited spectacular antibacterial activities against all tested microorganisms at a concentration of 1 mg/mL.

#### Minimum Inhibitory Concentration (MIC)

Based on the excellent results obtained in the inhibition zone test, it seemed appropriate to evaluate the Minimum Inhibitory Concentration (MIC), which is the highest dilution of the compound that shows a clear fluid with no development of turbidity. For this, eight ILs were selected based on their activity, and the results are summarized in Table 3.

**Table 3:** Antimicrobial activity expressed as MIC (μg/mL).

Compd	Antifungal Activity				Antibacterial Activity			
	*A. fumigatus*	*S. racemosum*	*G. candidum*	*C. albicans*	*S. pneumoniae*	*B. subtilis*	*P. aeruginosa*	*E. coli*
**2**	3.9	3.9	7.81	31.25	15.63	3.9	62.5	7.81
**5**	0.98	0.98	0.24	0.49	0.98	0.49	1.95	0.98
**12**	1.95	3.9	0.98	3.9	3.9	1.95	15.63	1.95
**13**	7.81	3.9	7.81	15.63	7.81	3.9	125	15.63
**14**	31.25	7.81	3.9	15.63	15.63	7.81	15.63	1.95
**16**	7.81	3.9	1.95	62.5	7.81	0.49	31.25	7.81
**19**	0.49	0.98	0.49	0.49	0.49	0.24	0.49	0.24
**20**	31.25	7.81	3.9	125	3.9	3.9	31.25	3.9
**Amphotericin B**	3.9	15.63	0.49	0.98	---	---	---	---
**Ampicillin**	---	---	---	---	3.9	0.49	---	---
**Gentamicin**	---	---	---	---	---	---	31.25	7.81

From the MIC values obtained, all compounds exhibited antibacterial activity of varying degrees as well as spectrum. In general and as expected, all tested ILs (**2**, **5**, **12**, **13**, **14**, **16**, **19**, and **20**) possessed similar antibacterial activities.

IL **5** (4-(dimethylamino)-1-(4-phenoxybutyl)pyridinium bromide) and IL **19** (4-(dimethylamino)-1-(4-phenoxybutyl)pyridinium tetrafluoroborate) exhibited particularly impressive antimicrobial activities in the series against all tested bacteria and fungi, with MIC values significantly lower than those of the standard controls. The excellent antibacterial activity of ILs **5** and **19** confirm our recently published results and allows us to unambiguously attribute this to the presence of the butylphenoxy group [20].

However, in this case, exchanging the halides (Br or Cl) with fluorinated anions (BF_4_, PF_6_ or CF_3_CO_2_) did not cause any obvious trends in the activity, and different activities were observed depending on the bacteria or fungi and the ionic liquid tested.

## 3. Experimental Section

### 3.1. Apparatus

All new compounds were characterized by ^1^H-NMR, ^13^C-NMR and IR spectroscopy, and LCMS. ^1^H-NMR (400 MHz) and ^13^C-NMR (100 MHz) spectra were measured in DMSO and D_2_O at room temperature. Chemical shifts (δ) were reported in ppm, with tetramethylsilane (TMS) as an internal standard (Bruker, Faellanden, Switzerland). The LCMS spectra were measured with a Micromass LCT mass spectrometer (Agilent Technologies, Waldbronn Germany). IR spectra were recorded on a KBr disc with a Shimadzu 8201 PC FT-IR spectrophotometer (ν_max_ in cm^−1^) (Shimadzu Scientific Instruments INC, Canby, OR, USA). The elemental analyses were given by using the 2400 Series II CHNS/O Elemental Analyzer (Perkin Elmer, Waltham, MA, USA). Ultrasound-assisted reactions were performed with a high-intensity ultrasonic processor SUB Aqua 5 Plus-Grant with a temperature controller (750 W), microprocessor controlled-2004. The ultrasonic frequency of the cleaning bath used is 25 KHz (Grant Scientific, Cambridgeshire, UK).

### 3.2. Synthesis

General procedures for the synthesis of imidazolium halides (**1**–**6**)*.* To a solution of 4-dimethylaminopyridine (2 g, 0.0163 mol) in 20 mL of toluene, was added the appropriate alkyl halide (1.1 eq) at room temperature. The mixture was placed in a closed vessel and exposed to ultrasound irradiation for 5 h at 80 °C using a sonication bath. The completion of the reaction was marked by the separation of oil or a solid from the initially obtained clear and homogenous mixture of 4-dimethylaminopyridine and alkyl halide in toluene. The product was isolated by extraction or filtration to remove the unreacted starting materials and solvent. Subsequently, the pyridinium IL was washed with (3 × 20 mL) of ethyl acetate followed by drying under reduced pressure.

General procedure for the methathesis reaction of (**1**–**6**) leading to compounds (**7**–**24**) under ultrasound irradiation. The quaternary salt (0.3 g, 1 eq) was dissolved in in 10 mL of dichloromethane to obtain a clear solution. To this was added (1 eq) of sodium tetrafluoroborate, potassium hexafluorophosphate or sodium trifluoroacetate. The mixture were placed in a closed vessel and exposed to irradiation for 45 min at 70 °C using a sonication bath. The cooled reaction mixture was filtered through Celite to remove the solid metal halide. Evaporation of the dichloromethane quantitatively afforded the desired ionic liquids.

### 3.3. Characterization

*4-(Dimethylamino)-1-(2-hydroxyethyl)pyridinium bromide* (**1**). This compound was obtained as white solid (3.26 g); mp 148–150 °C, ^1^H-NMR (D_2_O, 400 MHz,): δ = 3.20 (s, 6H), 3.94 (t, 2H), 4.24 (t, 2H), 6.90 (d, 2Ar-H), 8.01 (d, 2Ar-H); ^13^C-NMR (D_2_O, 100 MHz,): δ = 39.5 (2CH_3_), 59.3 (CH_2_), 60.5 (CH_2_), 107.6 (CH), 141.7 (CH), 156.6 (C); IR (KBr) ν_max_ 3213 (O–H), 3161 (C–H, sp^2^), 1566 (C=C), 1161 (C–N), 1157 (C–O), LCMS (M-Br) 167.2 found for C_9_H_15_N_2_O^+^; (Found: C, 43.66%; H, 6.05%; N, 11.40%. Calc. for C_9_H_15_BrN_2_O (247.13); C, 43.74%; H, 6.12%; N, 11.34%).

*4-(Dimethylamino)-1-(3-hydroxypropyl)pyridinium bromide* (**2**). This compound was obtained as white solid (3.63 g); mp 112–114 °C, ^1^H-NMR (D_2_O, 400 MHz,): δ = 2.09 (quintet, 3H), 3.20 (s, 6H), 3.62 (t, 2H), 4.24 (t, 2H), 6.89 (d, 2H), 8.03 (d, 2H); ^13^C-NMR (D_2_O, 100 MHz,): δ = 32.3 (CH_2_), 39.6 (2CH_3_), 54.6 (CH_2_), 57.9 (CH_2_), 107.7 (CH), 141.6 (CH), 156.4 (C); IR (KBr) ν_max_ 3212 (O–H), 3160 (C–H, sp^2^), 1565 (C‚C), 1163 (C–N), 1158 (C–O); LCMS (M-Br) 181.2 found for C_10_H_17_N_2_O^+^; (Found: C, 46.04%; H, 6.49%; N, 10.68%. Calc. for C_10_H_17_BrN_2_O (261.16); C, 45.99%; H, 6.56%; N, 10.73%).

*4-(Dimethylamino)-1-(2-methoxyethyl)pyridinium bromide* (**3**)*.* This compound was obtained as white solid (3.46 g); mp 184–186 °C, ^1^H-NMR (DMSO, 400 MHz,): δ = 1.11 (t, 3H), 3.25 (s, 3H), 3.67 (t, 2H), 3.87 (t, 2H), 4.29 (t, 2H), 6.88 (d, 2H), 8.00 (d, 2H); ^13^C-NMR (DMSO, 100 MHz,): δ = 39.6 (2CH_3_), 56.2 (CH_2_), 58.1 (CH_3_) 70.4 (CH_2_), 107.3 (CH), 142.2 (CH), 155.9 (C); ); IR (KBr) ν_max_ 3159 (C–H, sp^2^), 1563 (C=C), 1161 (C–N), 1156 (C–O); LCMS (M-Br) 181.2 found for C_10_H_17_N_2_O^+^; (Found: C, 45.91%; H, 6.51%; N, 10.80%. Calc. for C_10_H_17_BrN_2_O (261.16); C, 45.99%; H, 6.56%; N, 10.73%).

*4-(Dimethylamino)-1-(2-ethoxyethyl)pyridinium chloride* (**4**)*.* This compound was obtained as brown solid; mp >280 °C (decomp) (2.98 g); ^1^H-NMR (D_2_O, 400 MHz,): δ = 3.17 (s, 3H), 3.20 (s, 6H), 3.55 (q, 2H), 4.34 (t, 2H), 7.00 (d, 2H), 8.20 (d, 2H); ^13^C-NMR (D_2_O, 100 MHz,): δ = 14.1 (CH_3_), 39.5 (2CH_3_), 56.9 (CH_2_), 66.8 (CH_2_), 68.4 (CH_2_), 107.5 (CH), 141.7 (CH), 156.5 (C); ); IR (KBr) ν_max_ 3160 (C–H, sp^2^), 1565 (C=C), 1163 (C–N), 1158 (C–O); LCMS (M-Cl) 195.3 found for C_11_H_19_N_2_^+^; (Found: C, 57.19%; H, 8.35%; N, 12.22%. Calc. for C_11_H_19_ClN_2_O (230.73); C, 57.26%; H, 8.30%; N, 12.14%).

*4-(Dimethylamino)-1-(4-phenoxybutyl)pyridinium bromide* (**5**)*.* This compound was obtained as brown solid (4.77 g); mp 98–100 °C, ^1^H-NMR (DMSO, 400 MHz,): δ = 1.68 (quintet, 2H), 1.93 (quintet, 2H), 3.218 (s, 6H), 3.98 (t, 2H), 4.29 (t, 2H), 6.89–6.93 (m, 5Ar-H 7.05 (d, 2Ar-H), 8.40 (d, 2Ar-H); ^13^C-NMR (DMSO, 100 MHz,): δ = 25.2 (CH_2_), 27.2 (CH_2_), 39.7 (2CH_3_), 56.2 (CH_2_), 66.6 (CH_2_), 107.5 (CH), 114.4 (CH), 120.5 (CH), 129.4 (CH), 142.0 (CH), 155.8 (C), 158.4 (C); IR (KBr) ν_max_ 3131 (C–H Ar), 1599–1469 (C=C), 1166 (C–N), 1079 (C–O) cm^−1^; LCMS (M-Br) 271.4 found for C_17_H_23_N_2_O^+^; (Found: C, 58.07%; H, 6.54%; N, 8.04%. Calc. for C_17_H_23_BrN_2_O (351.28); C, 58.12%; H, 6.60%; N, 7.97%).

*1-(3-Cyanopropyl)-4-(dimethylamino)pyridinium chloride* (**6**)*.* This compound was obtained as white solid; mp 78–80 °C (2.88 g), ^1^H-NMR (D_2_O, 400 MHz,): δ = 2.25 (quintet, 2H), 2.61 (t, 2H), 3.22 (s, 6H), 4.27 (t, 2H), 6.92 (d, 2Ar-H), 8.06 (d, 2Ar-H); ^13^C-NMR (D_2_O, 100 MHz,): δ = 13.7 (CH_2_), 25.7 (CH_2_), 39.5 (2CH_3_), 56.1 (CH_2_), 107.9 (CH), 120.4 (C), 141.4 (CH), 156.6 (C); IR (KBr) ν_max_ 3131 (C–H Ar), 2247 (C–N), 1597–1471 (C=C), 1169 (C–N)cm^−1^; LCMS (M-Cl) 190.2 found for C_11_H_16_N_3_^+^; (Found: C, 58.46%; H, 7.06%; N, 18.71%. Calc. for C_11_H_16_ClN_3_ (225.72); C, 58.53%; H, 7.145%; N, 18.62%).

*4-(Dimethylamino)-1-(2-hydroxyethyl)pyridinium tetrafluoroborate* (**7**). This compound was obtained as yellow solid; mp 62–63 °C (0.29 g), ^1^H-NMR (DMSO, 400 MHz,): δ = 3.18 (s, 6H), 3.71 (m, 2H), 4.24 (t, 2H), 5.09 (s, 1H), 7.02 (d, 2Ar-H), 8.25 (d, 2Ar-H); ^13^C-NMR (DMSO, 100 MHz,): δ = 39.7 (2CH_3_), 58.9 (CH_2_), 60.0 (CH_2_), 107.3 (CH), 142.4 (CH), 155.9 (C); ^19^F-NMR (DMSO, 376.5 MHz): δ = −148.30; ^11^B-NMR (DMSO, 128 MHz): δ = −1.27; IR (KBr) ν_max_ 3214 (O–H), 3162 (C–H, sp^2^), 1568 (C=C), 1162 (C–N), LCMS (M-Br) 167.2 found for C_9_H_15_N_2_O^+^; (Found: C, 42.63%; H, 6.03%; N, 10.94%. Calc. for C_9_H_15_BF_4_N_2_O (254.03); C, 42.55%; H, 5.95%; N, 11.03%).

*4-(Dimethylamino)-1-(2-hydroxyethyl)pyridinium hexafluorophosphate* (**8**). This compound was obtained as white solid; mp 66–68 °C (0.37 g), ^1^H-NMR (DMSO, 400 MHz,): δ = 3.18 (s, 6H), 3.71 (m, 2H), 4.24 (t, 2H), 5.09 (s, 1H), 7.02 (d, 2Ar-H), 8.25 (d, 2Ar-H); ^13^C-NMR (DMSO, 100 MHz,): δ = 39.7 (2CH_3_), 58.9 (CH_2_), 60.0 (CH_2_), 107.3 (CH), 142.4 (CH), 155.9 (C); ^19^F-NMR (DMSO, 376.5 MHz): δ = −71.11, −69.18 (d); ^31^P-NMR (DMSO, 162 MHz): δ = −157.34–−130.99 (sep*t*); IR (KBr) ν_max_ 3210 (O–H), 3159 (C–H, sp^2^), 1563 (C=C), 1160 (C–N), LCMS (M-PF_6_) 167.2 found for C_9_H_15_N_2_O^+^; (Found: C, 34.55%; H, 4.76%; N, 9.04%. Calc. for C_9_H_15_F_6_N_2_OP (312.19); C, 34.62%; H, 4.84%; N, 8.97%).

*4-(Dimethylamino)-1-(2-hydroxyethyl)pyridinium trifluoroacetate* (**9**). This compound was obtained as white solid; mp 86–88 °C (0.32 g), ^1^H-NMR (DMSO, 400 MHz,): δ = 3.19 (s, 6H), 3.73 (m, 2H), 4.24 (t, 2H), 5.23 (s, 1H), 7.04 (d, 2Ar-H), 8.26 (d, 2Ar-H); ^13^C-NMR (DMSO, 100 MHz,): δ = 39.6 (2CH_3_), 59.0 (CH_2_), 60.1 (CH_2_), 107.3 (CH), 142.5 (CH), 155.9 (C);^19^F-NMR (DMSO, 376.5 MHz): δ = −73.49; IR (KBr) ν_max_ 3213 (O–H), 3161 (C–H, sp^2^), 1564 (C=C), 1158 (C–N), LCMS (M-CF_3_CO_2_) 167.2 found for C_9_H_15_N_2_O^+^; (Found: C, 47.04%; H, 5.23%; N, 10.08%. Calc. for C_11_H_15_F_3_N_2_O_3_ (280.24); C, 47.14%; H, 5.39%; N, 10.00%).

*4-(Dimethylamino)-1-(3-hydroxypropyl)pyridinium tetrafluoroborate* (**10**). This compound was obtained as oil (0.29 g), ^1^H-NMR (DMSO, 400 MHz,): δ = 1.93 (quintet, 3H), 3.20 (s, 6H), 3.41 (t, 2H), 4.25 (t, 2H), 7.05 (d, 2H), 8.31 (d, 2H); ^13^C-NMR (DMSO, 100 MHz,): δ = 33.1 (CH_2_), 39.7 (2CH_3_), 54.2 (CH_2_), 56.9 (CH_2_), 107.6 (CH), 141.2 (CH), 155.8 (C); ^19^F-NMR (DMSO, 376.5 MHz): δ = −148.36; ^11^B NMR (DMSO, 128 MHz): δ = −1.27; IR (NaCl) ν_max_ 3216 (O–H), 3167 (C–H, sp^2^), 1566 (C=C), 1166 (C–N), 1153 (C–O), LCMS (M-BF_4_) 181.2 found for C_10_H_17_N_2_O^+^; (Found: C, 44.92%; H, 6.30%; N, 10.53%. Calc. for C_10_H_17_BF_4_N_2_O (268.06); C, 44.81%; H, 6.39%; N, 10.45%).

*4-(Dimethylamino)-1-(3-hydroxypropyl)pyridinium hexafluorophosphate* (**11**). This compound was obtained as white semi-solid (0.37 g), ^1^H-NMR (DMSO, 400 MHz,): δ = 1.92 (quintet, 3H), 3.19 (s, 6H), 3.40 (t, 2H), 4.23 (t, 2H), 7.02 (d, 2H), 8.24 (d, 2H); ^13^C-NMR (DMSO, 100 MHz,): δ = 33.1 (CH_2_), 39.7 (2CH_3_), 54.2 (CH_2_), 56.9 (CH_2_), 107.6 (CH), 141.2 (CH), 155.8 (C); ^19^F-NMR (DMSO, 376.5 MHz): δ = −71.10, −69.22 (d); ^31^P-NMR (DMSO, 162 MHz): δ = −157.35–−131.00 (sept); IR (NaCl) ν_max_ 3211 (O–H), 3160 (C–H, sp^2^), 1565 (C=C), 1164 (C–N), 1152 (C–O),LCMS (M-PF_6_) 181.2 found for C_10_H_17_N_2_O^+^; (Found: C, 36.73%; H, 5.16%; N, 8.67%. Calc. for C_10_H_17_F_6_N_2_OP (326.22); C, 36.82%; H, 5.25%; N, 8.59%).

*4-(Dimethylamino)-1-(3-hydroxypropyl)pyridinium trifluoroacetate* (**12**). This compound was obtained as oil (0.32 g), ^1^H-NMR (DMSO, 400 MHz,): δ = 1.91 (quintet, 3H), 3.19 (s, 6H), 3.39 (t, 2H), 4.26 (t, 2H), 7.03 (d, 2H), 8.32 (d, 2H); ^13^C-NMR (DMSO, 100 MHz,): δ = 33.1 (CH_2_), 39.7 (2CH_3_), 54.1 (CH_2_), 56.9 (CH_2_), 107.6 (CH), 142.2 (CH), 155.8 (C); ^19^F-NMR (DMSO, 376.5 MHz): δ = −73.56; IR (NaCl) ν_max_ 3209 (O–H), 3164 (C–H, sp^2^), 1562 (C=C), 1162 (C–N), 1150 (C–O); LCMS (M-CF_3_CO_2_) 181.2 found for C_10_H_17_N_2_O^+^; (Found: C, 49.06%; H, 5.73%; N, 9.61%. Calc. for C_12_H_17_F_3_N_2_O_3_ (294.27); C, 48.98%; H, 5.82%; N, 9.52%).

*4-(Dimethylamino)-1-(2-methoxyethyl)pyridinium tetrafluoroborate* (**13**)*.* This compound was obtained as white solid (0.29 g); mp 98–100 °C, ^1^H-NMR (DMSO, 400 MHz,): δ = 3.19 (s, 6H), 3.24 (s, 3H), 3.67 (t, 2H), 4.34 (t, 2H), 7.02 (d, 2H), 8.23 (d, 2H); ^13^C-NMR (DMSO, 100 MHz,): δ = 39.6 (2CH_3_), 56.2 (CH_2_), 58.1 (CH_3_) 70.4 (CH_2_), 107.4 (CH), 142.3 (CH), 155.9 (C); ^19^F-NMR (DMSO, 376.5 MHz): δ = −148.33; ^11^B NMR (DMSO, 128 MHz): δ = −1.26; IR (NaCl) ν_max_ 3161 (C–H, sp^2^), 1563 (C=C), 1163 (C–N), 1156 (C–O) LCMS (M-BF_4_) 181.2 found for C_10_H_17_N_2_O^+^; (Found: C, 44.84%; H, 6.33%; N, 10.51%. Calc. for C_10_H_17_BF_4_N_2_O (268.06); C, 44.81%; H, 6.39%; N, 10.45%).

*4-(Dimethylamino)-1-(2-methoxyethyl)pyridinium hexafluorophosphate* (**14**)*.* This compound was obtained as white solid; mp 80–82 °C (0.34 g), ^1^H-NMR (DMSO, 400 MHz,): δ = 3.17 (s, 6H), 3.25 (s, 3H), 3.67 (t, 2H), 4.34 (t, 2H), 7.00 (d, 2H), 8.20 (d, 2H); ^13^C-NMR (DMSO, 100 MHz,): δ = 39.6 (2CH_3_), 56.2 (CH_2_), 58.1 (CH_3_) 70.4 (CH_2_), 107.3 (CH), 142.2 (CH), 155.9 (C); ^19^F-NMR (DMSO, 376.5 MHz): δ = −71.10, −69.21 (d); ^31^P-NMR (DMSO, 162 MHz): δ = −157.31–−130.99 (sept); IR (NaCl) ν_max_ 3159 (C–H, sp^2^), 1564 (C=C), 1161 (C–N), 1158 (C–O); LCMS (M-PF_6_) 181.2 found for C_10_H_17_N_2_O^+^; (Found: C, 36.74%; H, 5.19%; N, 8.66%. Calc. for C_10_H_17_F_6_N_2_OP (326.22); C, 36.82%; H, 5.25%; N, 8.59%).

*4-(Dimethylamino)-1-(2-methoxyethyl)pyridinium trifluoroacetate* (**15**)*.* This compound was obtained as oil (0.31 g), ^1^H-NMR (DMSO, 400 MHz,): δ = 3.18 (s, 6H), 3.23 (s, 3H), 3.66 (t, 2H), 4.39 (t, 2H), 7.05 (d, 2H), 8.30 (d, 2H); ^13^C-NMR (DMSO, 100 MHz,): δ = 39.7 (2CH_3_), 56.0 (CH_2_), 58.1 (CH_3_) 70.5 (CH_2_), 107.4 (CH), 142.4 (CH), 155.9 (C); ^19^F-NMR (DMSO, 376.5 MHz): δ = −74.34; IR (NaCl) ν_max_ 3158 (C–H, sp^2^), 1560 (C=C), 1160 (C–N), 1158 (C–O); LCMS (M-CF_3_CO_2_) 181.2 found for C_10_H_17_N_2_O^+^; (Found: C, 49.06%; H, 5.74%; N, 9.59%. Calc. for C_12_H_17_F_3_N_2_O_3_ (294.27); C, 48.98%; H, 5.82%; N, 9.52%).

*4-(Dimethylamino)-1-(2-ethoxyethyl)pyridinium tetrafluoroborate* (**16**)*.* This compound was obtained as oil (0.34 g), ^1^H-NMR (DMSO, 400 MHz,): δ = 1.04 (t, 3H), 3.18 (s, 3H), 3.43 (q, 2H), 3.70 (t, 2H), 4.33 (t, 2H), 7.01 (d, 2H), 8.20 (d, 2H); ^13^C-NMR (DMSO, 100 MHz,): δ = 14.7 (CH_3_), 39.5 (2CH_3_), 56.4 (CH_2_), 65.5 (CH_2_), 68.2 (CH_2_), 107.3 (CH), 142.2 (CH), 156.5 (C); ^19^F-NMR (DMSO, 376.5 MHz): δ = −148.35; ^11^B NMR (DMSO, 128 MHz): δ = −1.20; IR (NaCl) ν_max_ 3161 (C–H, sp^2^), 1564 (C=C), 1160 (C–N), 1158 (C–O); LCMS (M-BF_4_) 195.3 found for C_11_H_19_N_2_O^+^; (Found: C, 46.76%; H, 6.71%; N, 7.01%. Calc. for C_11_H_19_BF_4_N_2_O (282.09); C, 46.84%; H, 6.79%; N, 9.93%).

*4-(Dimethylamino)-1-(2-ethoxyethyl)pyridinium hexafluorophosphate* (**17**)*.* This compound was obtained as oil (0.42 g), ^1^H-NMR (DMSO, 400 MHz,): δ = 1.05 (t, 3H), 3.19 (s, 3H), 3.43 (q, 2H), 3.70 (t, 2H), 4.33 (t, 2H), 7.02 (d, 2H), 8.21 (d, 2H); ^13^C-NMR (DMSO, 100 MHz,): δ = 14.7 (CH_3_), 39.6 (2CH_3_), 56.4 (CH_2_), 65.5 (CH_2_), 68.2 (CH_2_), 107.3 (CH), 142.3 (CH), 155.9 (C); ^19^F-NMR (DMSO, 376.5 MHz): δ = −71.13, −69.24 (d); ^31^P-NMR (DMSO, 162 MHz): δ = −157.34–−130.99 (sept); IR (NaCl) ν_max_ 3158 (C–H, sp^2^), 1564 (C=C), 1160 (C–N), 1157 (C–O); LCMS (M-PF_6_) 195.3 found for C_11_H_19_N_2_O^+^; (Found: C, 38.77%; H, 5.57%; N, 8.32%. Calc. for C_11_H_19_F_6_N_2_OP (340.25); C, 38.83%; H, 5.63%; N, 8.23%).

*4-(Dimethylamino)-1-(2-ethoxyethyl)pyridinium trifluoroacetate* (**18**)*.* This compound was obtained as oil (0.36 g), ^1^H-NMR (DMSO, 400 MHz,): δ = 1.05 (t, 3H), 3.20 (s, 3H), 3.45 (q, 2H), 3.71 (t, 2H), 4.37 (t, 2H), 7.05 (d, 2H), 8.28 (d, 2H); ^13^C-NMR (DMSO, 100 MHz,): δ = 13.7 (CH_3_), 39.6 (2CH_3_), 55.3 (CH_2_), 64.5 (CH_2_), 67.2 (CH_2_), 106.3 (CH), 141.3 (CH), 154.9 (C); ^19^F-NMR (DMSO, 376.5 MHz): δ = −73.57; IR (NaCl) ν_max_ 3160 (C–H, sp^2^), 1564 (C=C), 1162 (C–N), 1159 (C–O); LCMS (M-CF_3_CO_2_) 195.3 found for C_11_H_19_N_2_O^+^; (Found: C, 50.56%; H, 6.13%; N, 9.16%. Calc. for C_13_H_19_F_3_N_2_O_3_ (308.30); C, 50.65%; H, 6.21%; N, 9.09%).

*4-(Dimethylamino)-1-(4-phenoxybutyl)pyridinium tetrafluoroborate* (**19**)*.* This compound was obtained as white solid (0.28 g); mp 106–107 °C, ^1^H-NMR (DMSO, 400 MHz,): δ = 1.68 (quintet, 2H), 1.94 (quintet, 2H), 3.18 (s, 6H), 3.98 (t, 2H), 4.26 (t, 2H), 6.90–7.04 (m, 5Ar-H), 7.27 (d, 2Ar-H), 8.34 (d, 2Ar-H); ^13^C-NMR (DMSO, 100 MHz,): δ = 25.2 (CH_2_), 27.2 (CH_2_), 39.7 (2CH_3_), 56.3 (CH_2_), 66.6 (CH_2_), 107.6 (CH), 114.4 (CH), 120.5 (CH), 129.4 (CH), 141.9 (CH), 155.8 (C), 158.4 (C); ^19^F-NMR (DMSO, 376.5 MHz): δ = −148.34; ^11^B NMR (DMSO, 128 MHz): δ = −1.22; IR (KBr) ν_max_ 3132 (C–H Ar), 1600–1471 (C=C), 1164 (C–N), 1081 (C–O) cm^−1^; LCMS (M-BF_4_) 271.4 found for C_17_H_23_N_2_O^+^; (Found: C, 57.07%; H, 6.41%; N, 7.89%. Calc. for C_17_H_23_BF_4_N_2_O (358.18); C, 57.01%; H, 6.47%; N, 7.82%).

*4-(Dimethylamino)-1-(4-phenoxybutyl)pyridinium hexafluorophosphate* (**20**)*.* This compound was obtained as white solid (0.33 g); mp 128–130 °C, ^1^H-NMR (DMSO, 400 MHz,): δ = 1.68 (quintet, 2H), 1.93 (quintet, 2H), 3.18 (s, 6H), 3.97 (t, 2H), 4.29 (t, 2H), 6.89–7.06 (m, 5Ar-H), 7.26 (d, 2Ar-H), 8.40 (d, 2Ar-H); ^13^C-NMR (DMSO, 100 MHz,): δ = 25.2 (CH_2_), 27.2 (CH_2_), 39.7 (2CH_3_), 56.2 (CH_2_), 66.6 (CH_2_), 107.5 (CH), 114.4 (CH), 120.5 (CH), 129.4 (CH), 142.0 (CH), 155.8 (C), 158.4 (C); ^19^F-NMR (DMSO, 376.5 MHz): δ = −71.10, −69.21 (d); ^31^P-NMR (DMSO, 162 MHz): δ = −157.31–−130.96 (sept); IR (KBr) ν_max_ 3131 (C–H Ar), 1598–1471 (C=C), 1165 (C–N), 1079 (C–O) cm^−1^; LCMS (M-PF_6_) 271.4 found for C_17_H_23_N_2_O^+^; (Found: C, 48.97%; H, 5.50%; N, 6.81%. Calc. for C_17_H_23_F_6_N_2_OP (416.34); C, 49.04%; H, 5.57%; N, 6.73%).

*4-(Dimethylamino)-1-(4-phenoxybutyl)pyridinium trifluoroacetate* (**21**)*.* This compound was obtained as oil (0.30 g), ^1^H-NMR (DMSO, 400 MHz,): δ = 1.69 (quintet, 2H), 1.94 (quintet, 2H), 3.18 (s, 6H), 3.98 (t, 2H), 4.24 (t, 2H), 6.90–6.94 (m, 5Ar-H), 7.27 (d, 2Ar-H), 8.30 (d, 2Ar-H); ^13^C-NMR (DMSO, 100 MHz,): δ = 24.1 (CH_2_), 26.0 (CH_2_), 39.6 (2CH_3_), 53.7 (CH_2_), 55.1 (CH_2_), 106.5 (CH), 113.2 (CH), 119.3 (CH), 128.4 (CH), 140.8 (CH), 154.6 (C), 157.2 (C); ^19^F-NMR (DMSO, 376.5 MHz): δ = −73.58; IR (NaCl) ν_max_ 3130(C–H Ar), 1601–1473 (C=C), 1165 (C-N), 1080 (C-O) cm^−1^; LCMS (M-CF_3_CO_2_) 271.4 found for C_17_H_23_N_2_O^+^; (Found: C, 59.25%; H, 5.57%; N, 7.37%. Calc. for C_19_H_23_F_3_N_2_O_3_ (384.39); C, 59.37%; H, 6.03%; N, 7.29%).

*1-(3-Cyanopropyl)-4-(dimethylamino)pyridinium tetrafluoroborate* (**22**)*.* This compound was obtained as white solid (0.33 g); mp 120–121 °C, ^1^H-NMR (DMSO, 400 MHz,): δ = 2.12 (quintet, 2H), 2.56 (t, 2H), 3.19 (s, 6H), 4.22 (t, 2H), 7.04 (d, 2Ar-H), 8.26 (d, 2Ar-H); ^13^C-NMR (DMSO, 100 MHz,): δ = 13.3 (CH_2_), 25.8 (CH_2_), 39.5 (2CH_3_), 55.4 (CH_2_), 107.7 (CH), 119.5 (C), 141.9 (CH), 155.9 (C); ^19^F-NMR (DMSO, 376.5 MHz): δ = −148.41; ^11^B NMR (DMSO, 128 MHz): δ = −1.27; IR (KBr) ν_max_ 3131 (C-H Ar), 2251 (CN), 1598–1469 (C=C), 1170 (C–N) cm^−1^LCMS (M-BF_4_) 190.2 found for C_11_H_16_N_3_^+^; (Found: C, 47.60%; H, 5.74%; N, 15.23%. Calc. for C_11_H_16_ F_4_N_3_ (277.07); C, 47.68%; H, 5.82%; N, 15.17%).

*1-(3-Cyanopropyl)-4-(dimethylamino)pyridinium hexafluorophosphate* (**23**)*.* This compound was obtained as white solid (0.41 g); mp 121–122 °C, ^1^H-NMR (DMSO, 400 MHz,): δ = 2.12 (quintet, 2H), 2.56 (t, 2H), 3.19 (s, 6H), 4.22 (t, 2H), 7.04 (d, 2Ar-H), 8.26 (d, 2Ar-H); ^13^C-NMR (DMSO, 100 MHz,): δ = 13.4 (CH_2_), 25.8 (CH_2_), 39.7 (2CH_3_), 55.5 (CH_2_), 107.7 (CH), 119.5 (C), 142.0 (CH), 156.0 (C); ^19^F-NMR (DMSO, 376.5 MHz): δ = −71.12, −69.23 (d); ^31^P-NMR (DMSO, 162 MHz): δ = −157.36–−131.02 (sept); IR (KBr) ν_max_ 3130 (C–H Ar), 2246 (CN), 1599–1471 (C=C), 1169 (C-N)cm^−1^; LCMS (M-PF_6_) 190.2 found for C_11_H_16_N_3_^+^; (Found: C, 39.35%; H, 4.74%; N, 12.61%. Calc. for C_11_H_16_F_6_N_3_P (335.23); C, 39.41%; H, 4.81%; N, 12.53%).

*1-(3-Cyanopropyl)-4-(dimethylamino)pyridinium trifluoroacetate* (**24**). This compound was obtained as oil (0.37 g), NMR (DMSO, 400 MHz,): δ = 2.12 (quintet, 2H), 2.57 (t, 2H), 3.19 (s, 6H), 4.24 (t, 2H), 7.06 (d, 2Ar-H), 8.31 (d, 2Ar-H); ^13^C-NMR (DMSO, 100 MHz,): δ = 13.3 (CH_2_), 25.9 (CH_2_), 39.7 (2CH_3_), 55.4 (CH_2_), 107.7 (CH), 119.6 (C), 142.0 (CH), 156.0 (C); ^19^F-NMR (DMSO, 376.5 MHz): δ = −73.45; IR (NaCl) ν_max_ 3132 (C–H Ar), 2247 (CN), 1597–1476 (C=C), 1168 (C–N) cm^−1^; LCMS (M-CF_3_CO_2_) 190.2 found for C_11_H_16_N_3_^+^; (Found: C, 51.55%; H, 5.25%; N, 13.94%. Calc. for C_13_H_16_F_3_N_3_O_2_ (303.28); C, 51.48%; H, 5.32%; N, 13.86%).

### 3.4. Determination of Minimum Inhibitory Concentrations

Minimum inhibitory concentrations (MICs) were determined using the broth microdilution method based on recommended protocolemployed by the Clinical and Laboratory Standards Institute [27]. Tested compounds were dissolved in sterile, distilled water and diluted to a final concentration of 512 µg/mL in Mueller-Hinton broth (Becton Dickinson, USA) [28]. Two-fold, serially-diluted test compounds were dispensed into each of the 96 wells of a standard microdilution plates. The direct colony suspension method was used for inoculum preparation. Bacterial suspension was prepared by direct transfer of colonies from 24 h agar plates to Mueller Hinton broth. Bacterial suspensions were adjusted using bacterial counting chamber to contain approximately 1 × 10^8^ CFU/mL. A 50 µL volume of each bacterial suspension was mixed with 50 µL serially diluted tested compound in 96 microdilution plate according to the microdilution method [26]. Uninoculated wells were prepared as control samples. Plates were incubated at 35 °C for 24 h. The minimum (inhibitory) bactericidal concentration was defined as the lowest concentration of test compound producing no visible growth. Confirmation for MIC was achieved by transfer of aliquots from wells containing no growth on to nutrient agar plates and tested for colony formation upon subculturing. Given values of obtained MIC values are means of three independent experiments.

## 4. Conclusions

In summary, new functionalized 4-dimethylaminopyridinium-based ionic liquids (ILs) were synthesized using eco-friendly, ultrasound-assisted reactions which afforded many advantages, such as the reduction of reaction time and increase in yields. The MIC results show that the ILs studied display excellent antimicrobial activity, especially ILs **5** and **19**. Their activities are greatly improved by the presence of the butylphenoxy group.

## Supplementary Materials

Supplementary data (^1^H-NMR, ^13^C-NMR, ^11^B-NMR, ^19^F-NMR, and ^31^P-NMR) associated with this article are available at: http://www.mdpi.com/1420-3049/20/08/14936/s1.
