# Supplementary figures and images for: Eco-Friendly Synthesis of a New Class of Pyridinium-Based Ionic Liquids with Attractive Antimicrobial Activity
Source: Molecules. 2015 Aug 14;20(8):14936–49. doi: 10.3390/molecules200814936 (PMC6331848; doi:10.3390/molecules200814936)

—4.70  
—4.25  
—4.24  
—4.23  
—3.95  
—3.94  
—3.94  
—3.93

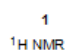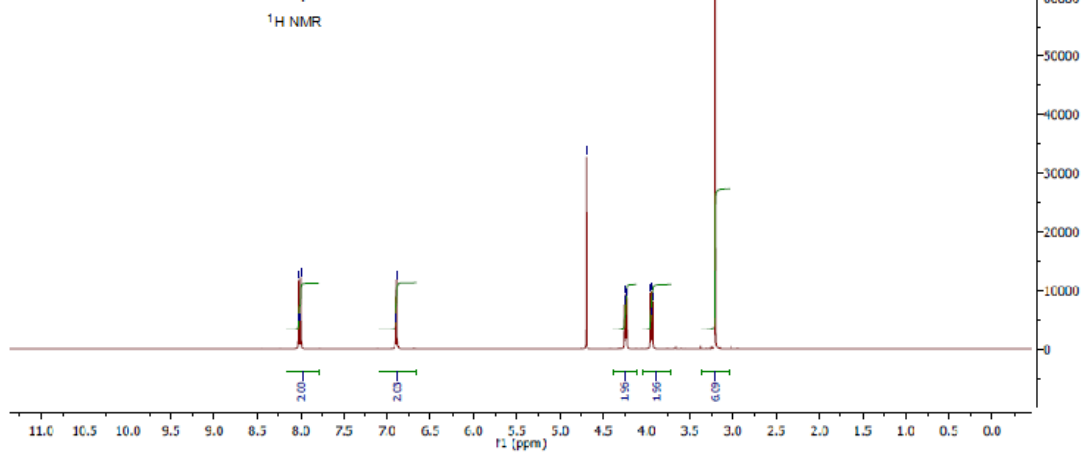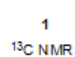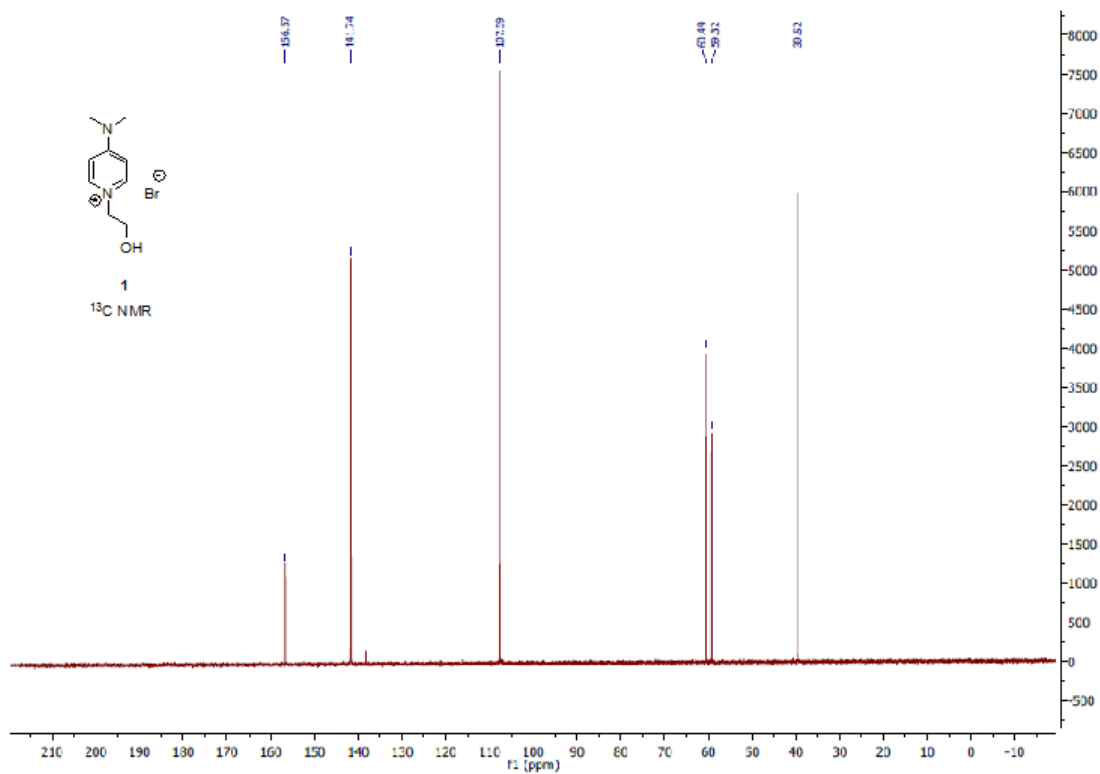

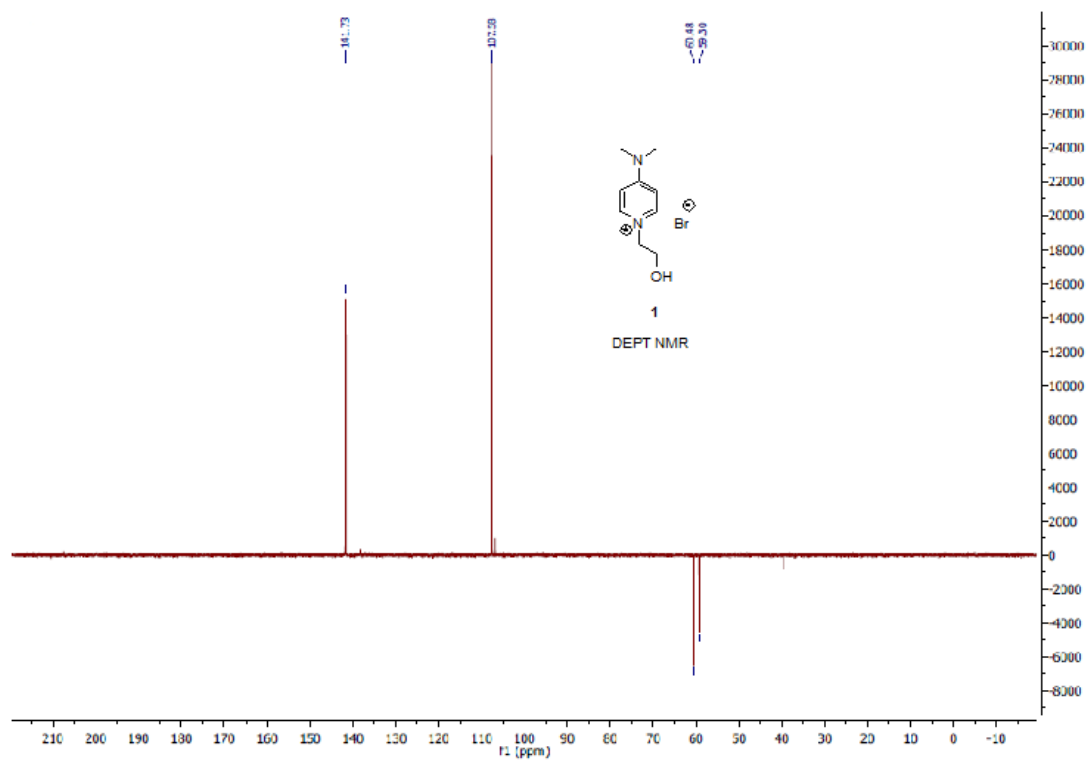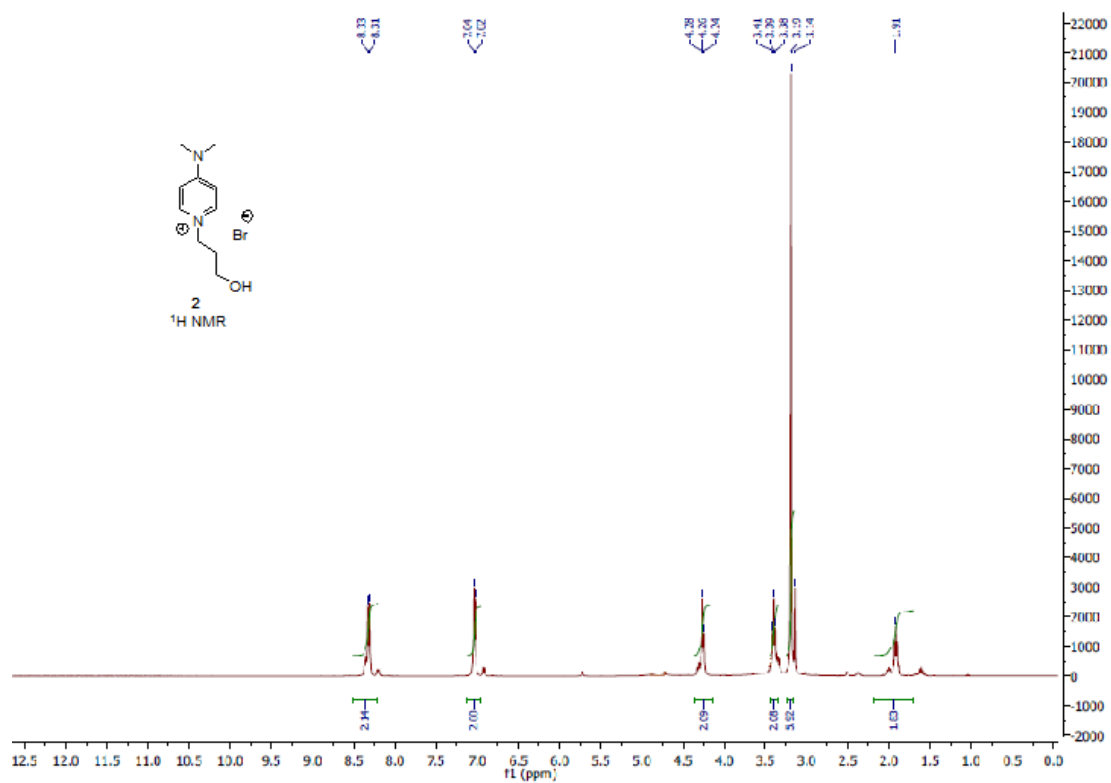

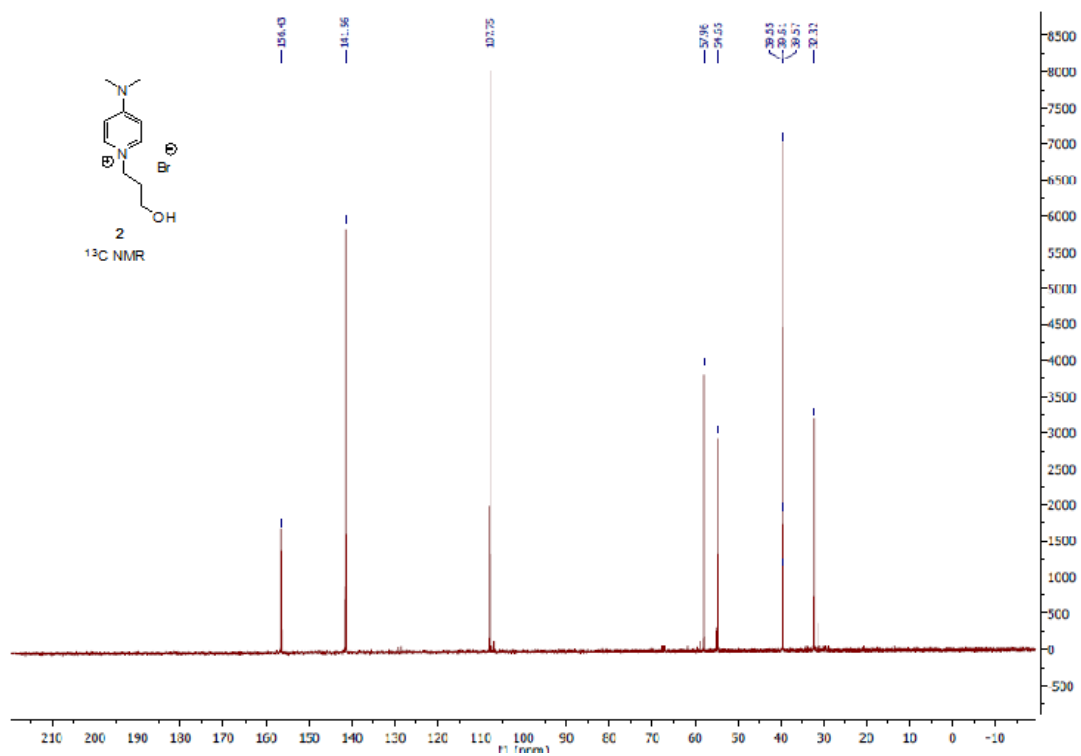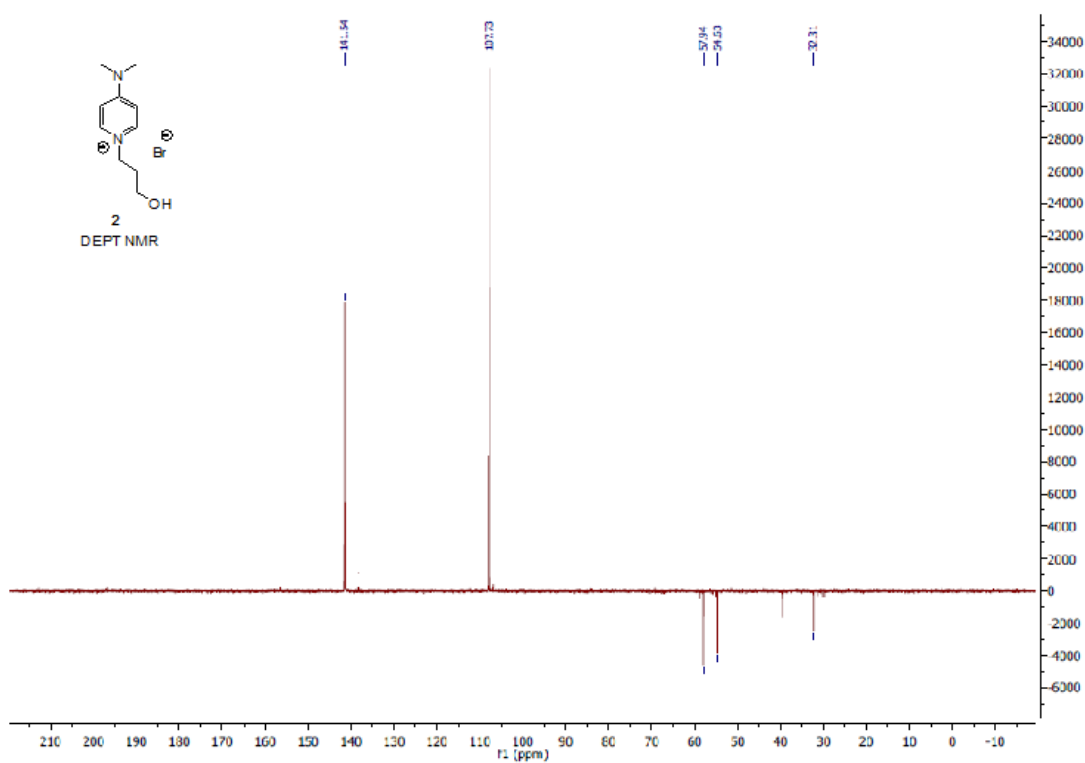

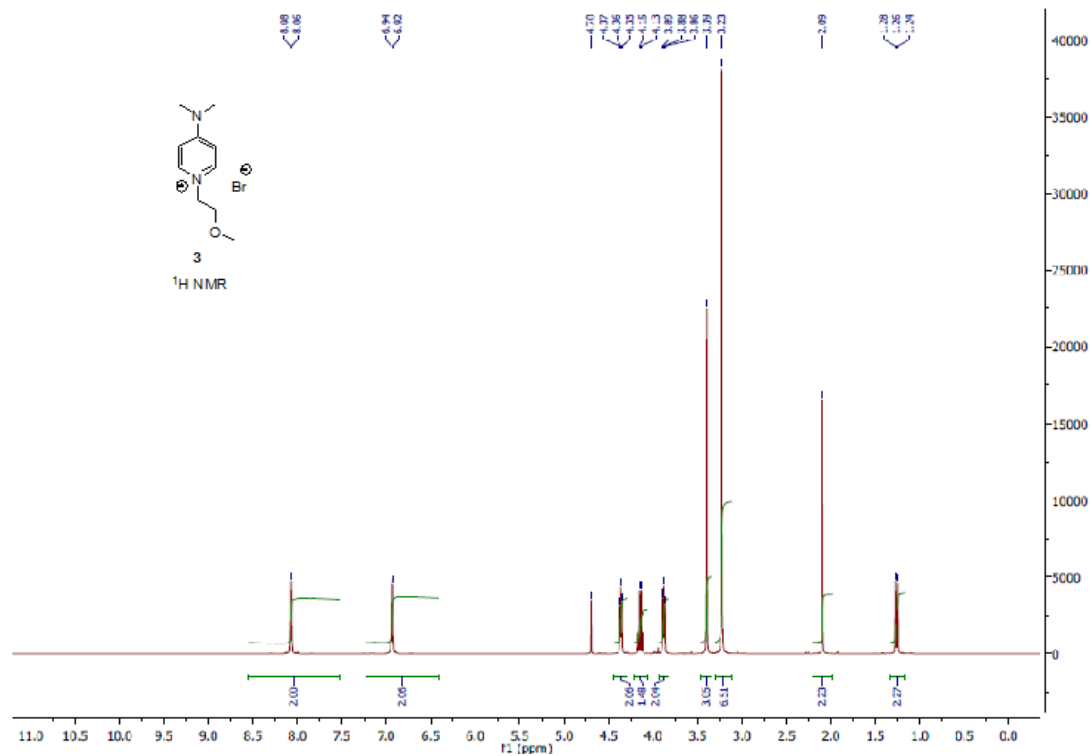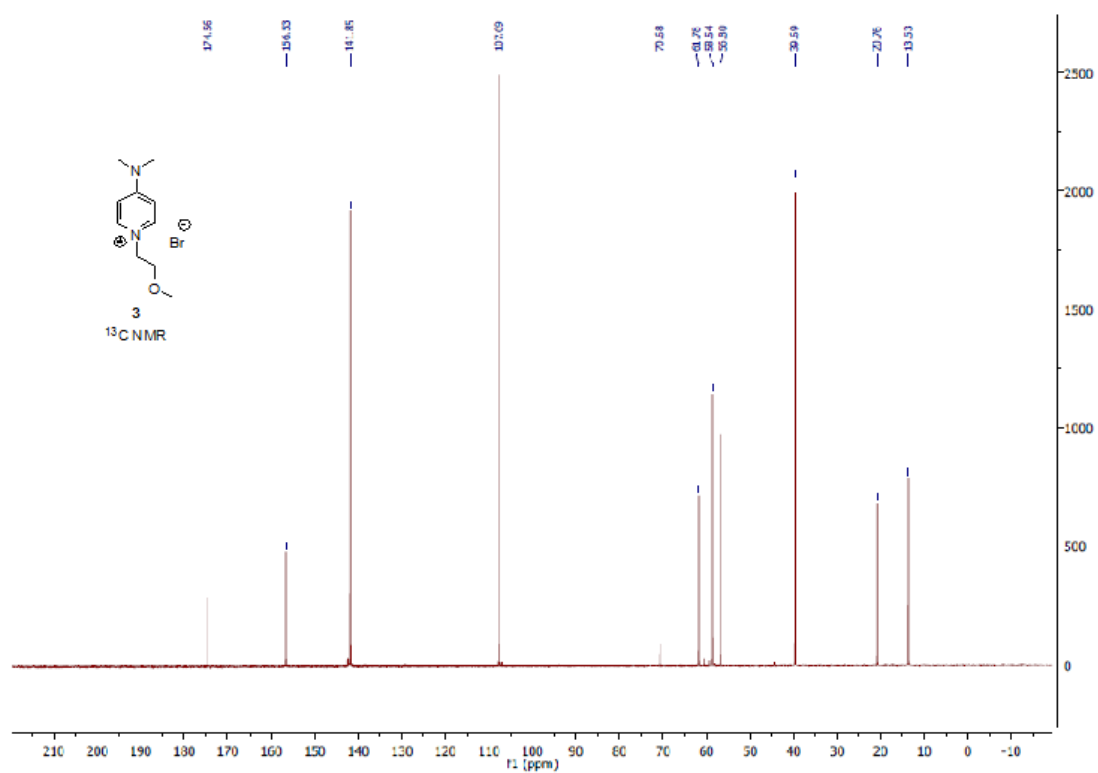

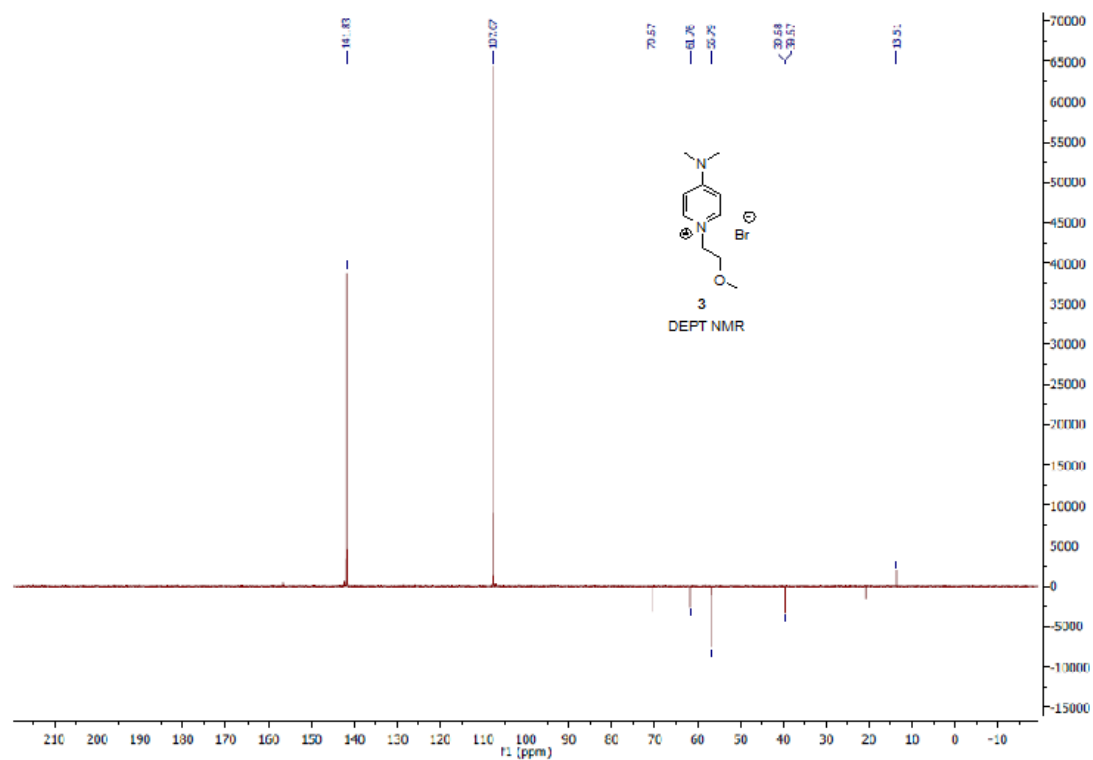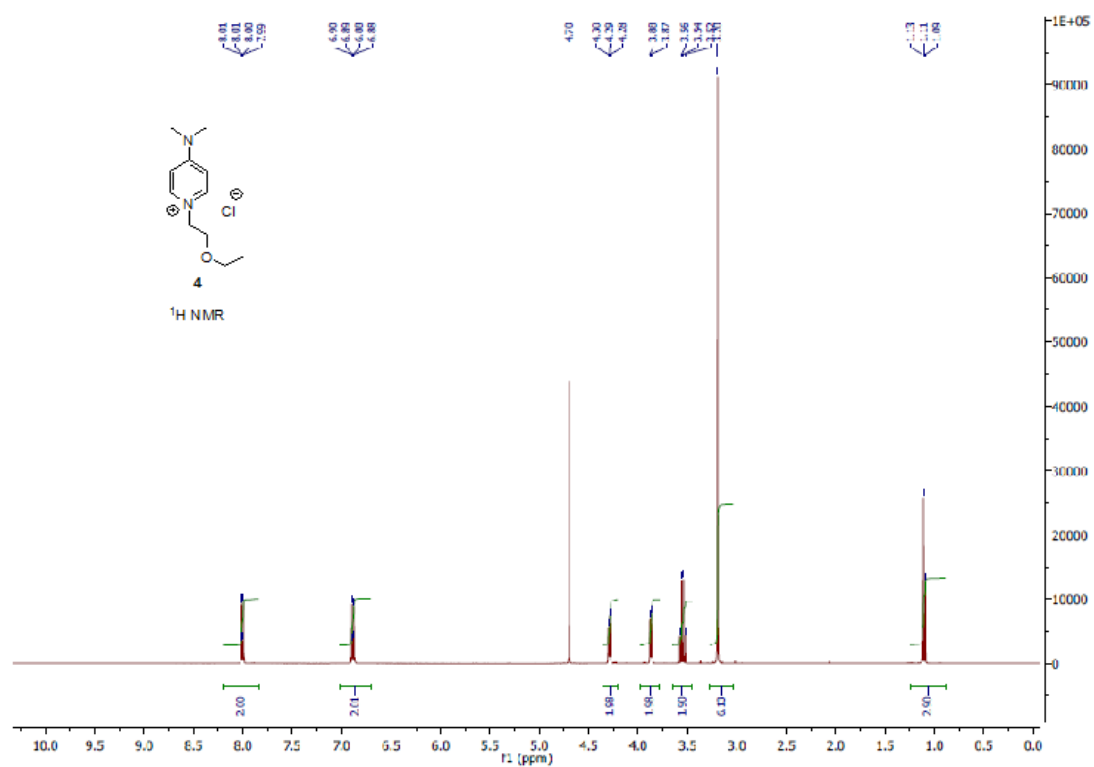

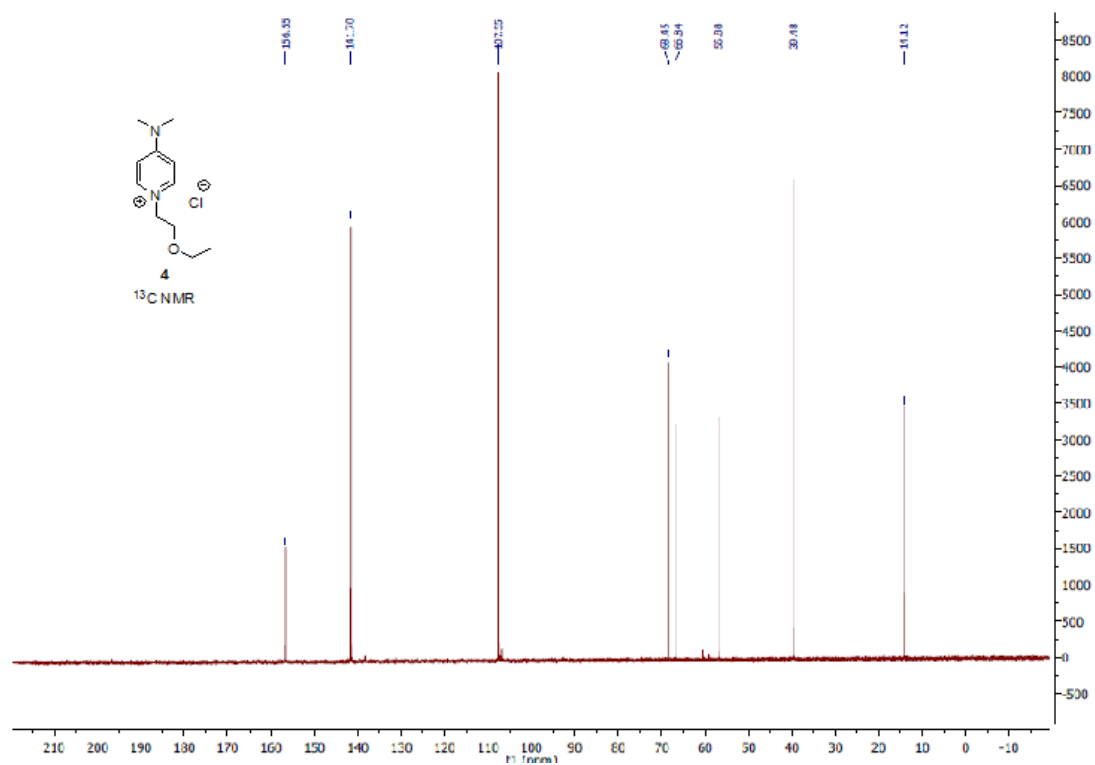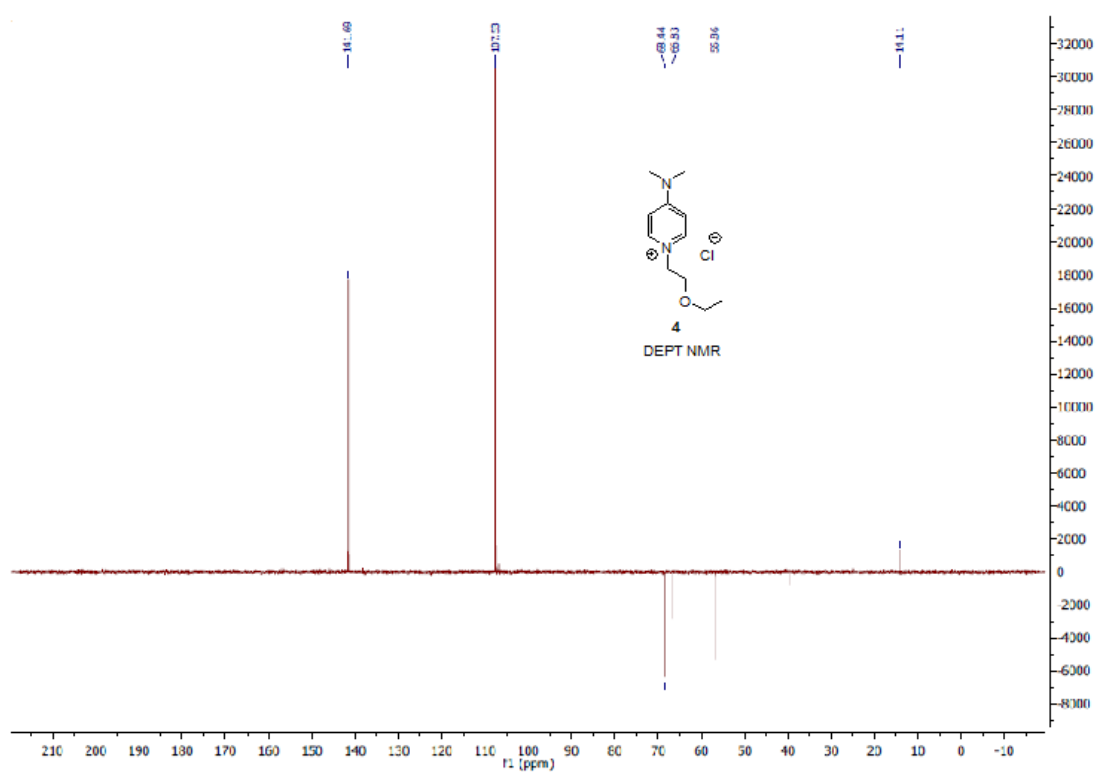

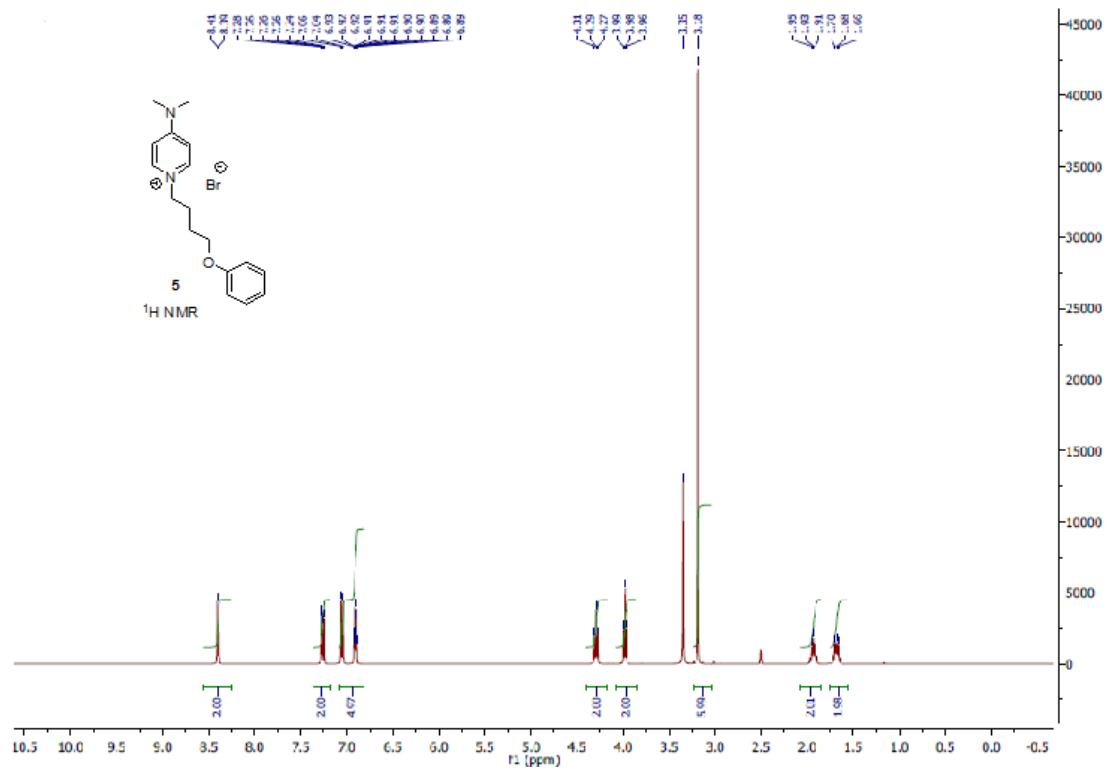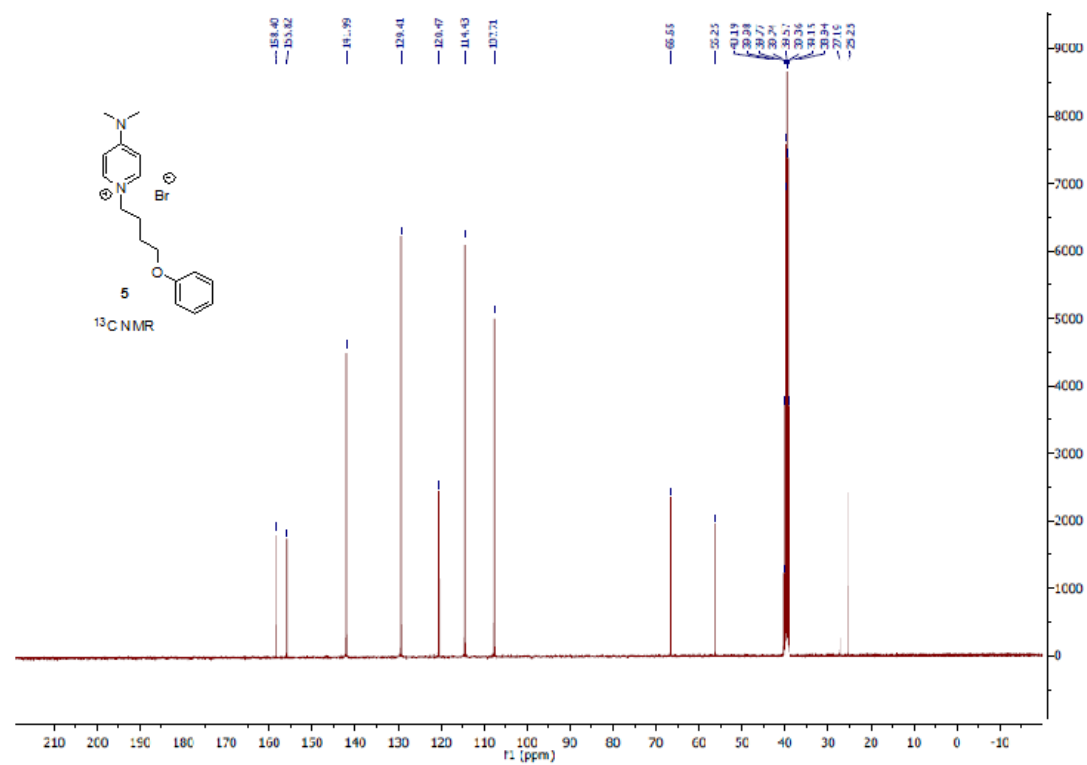

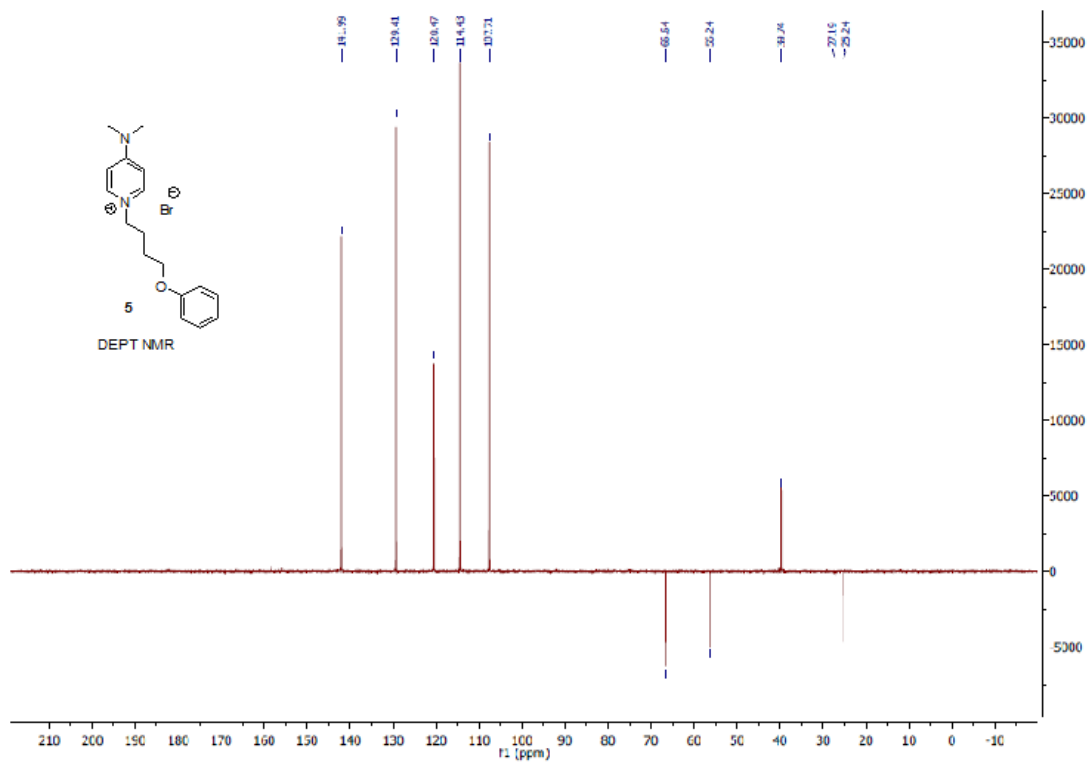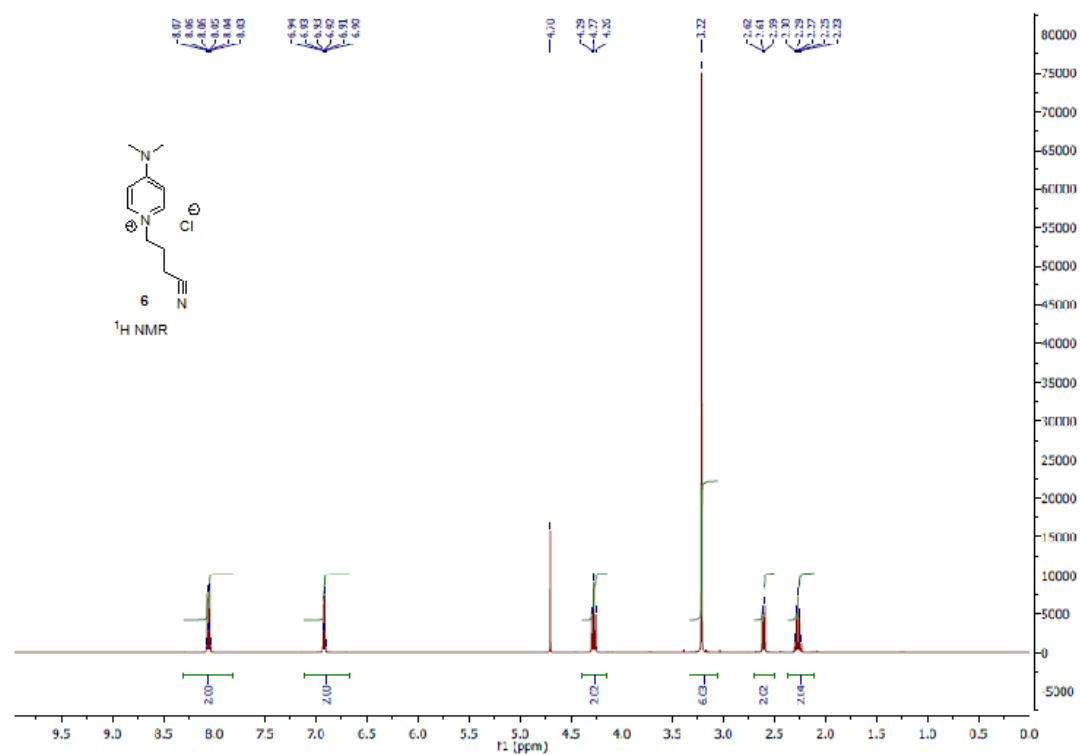

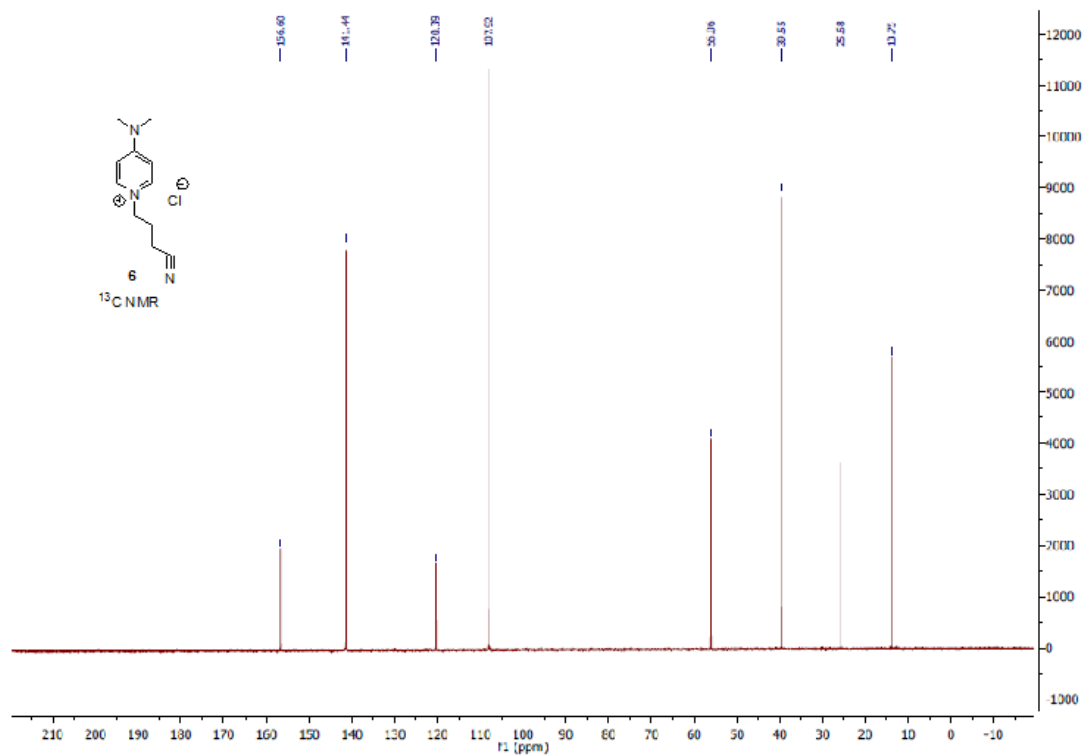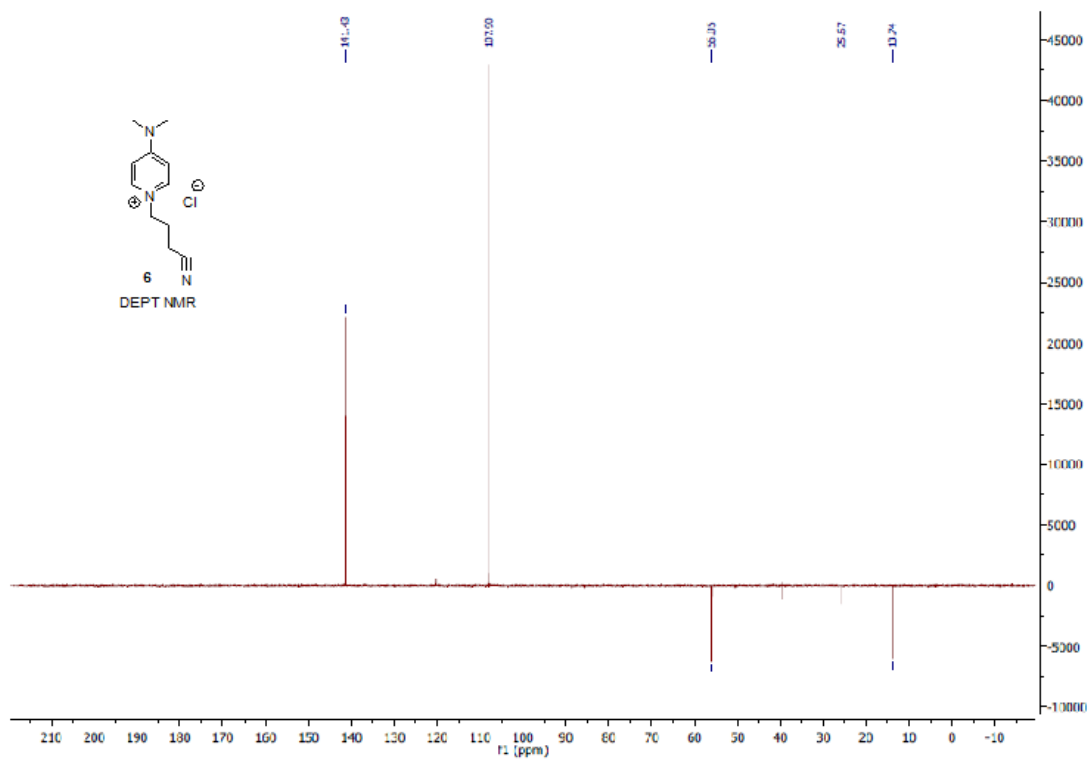

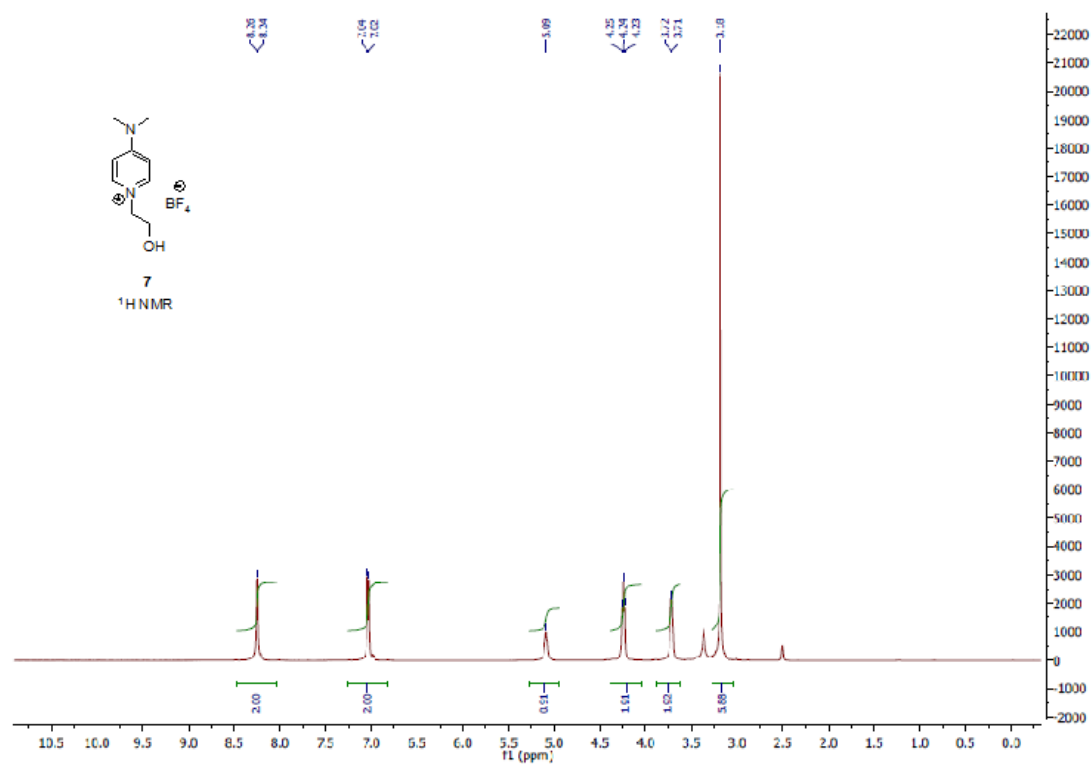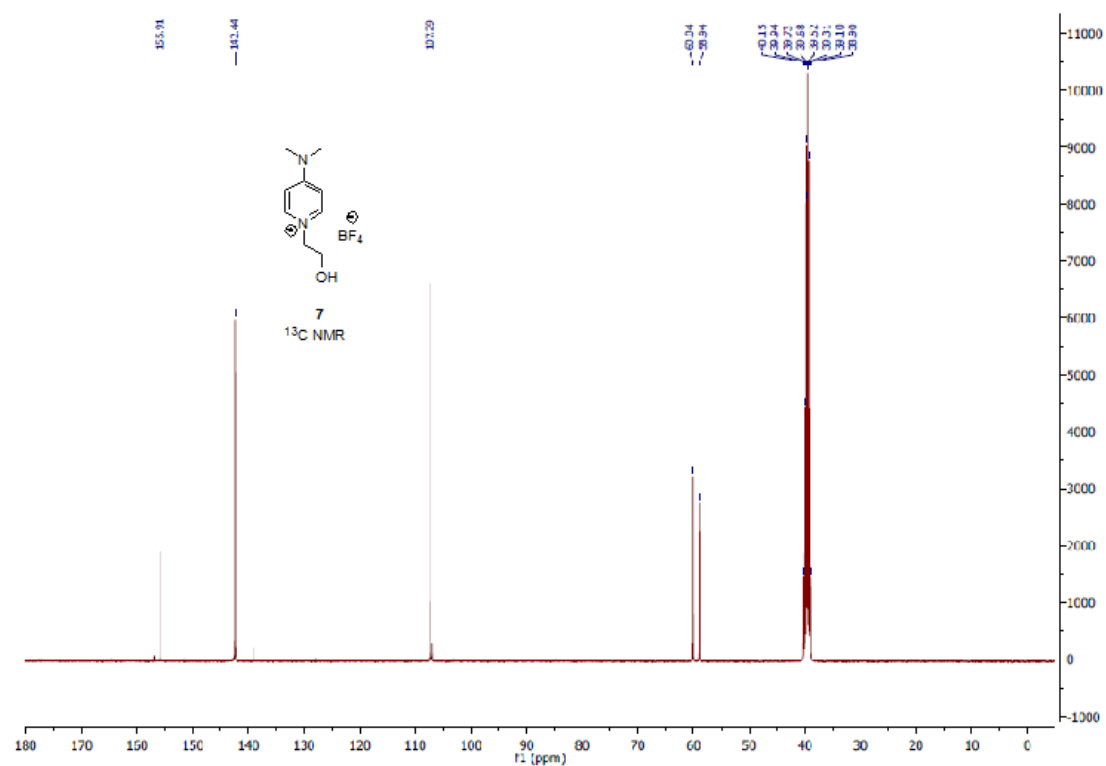

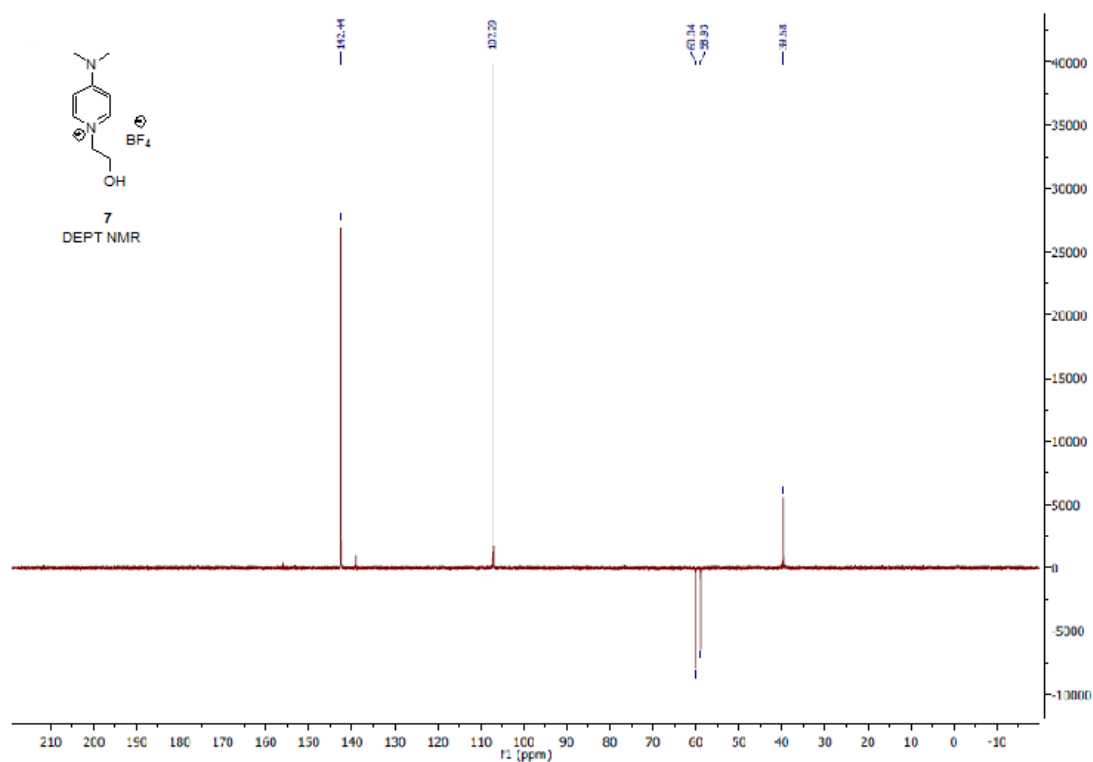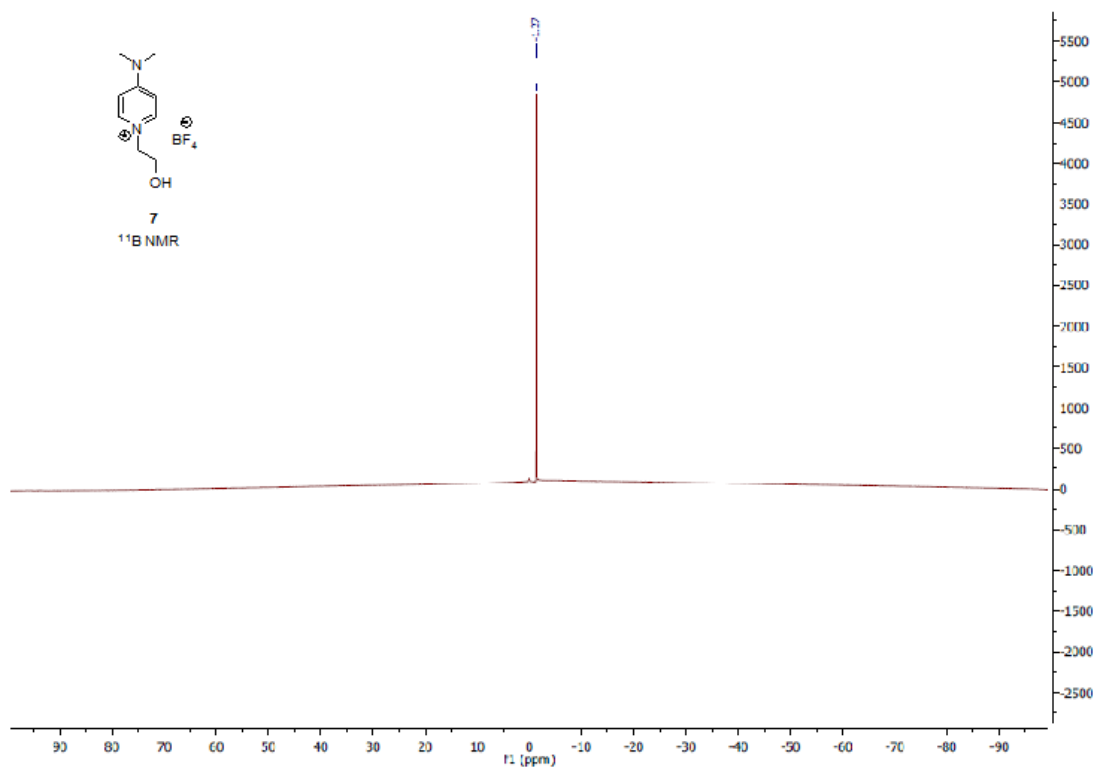

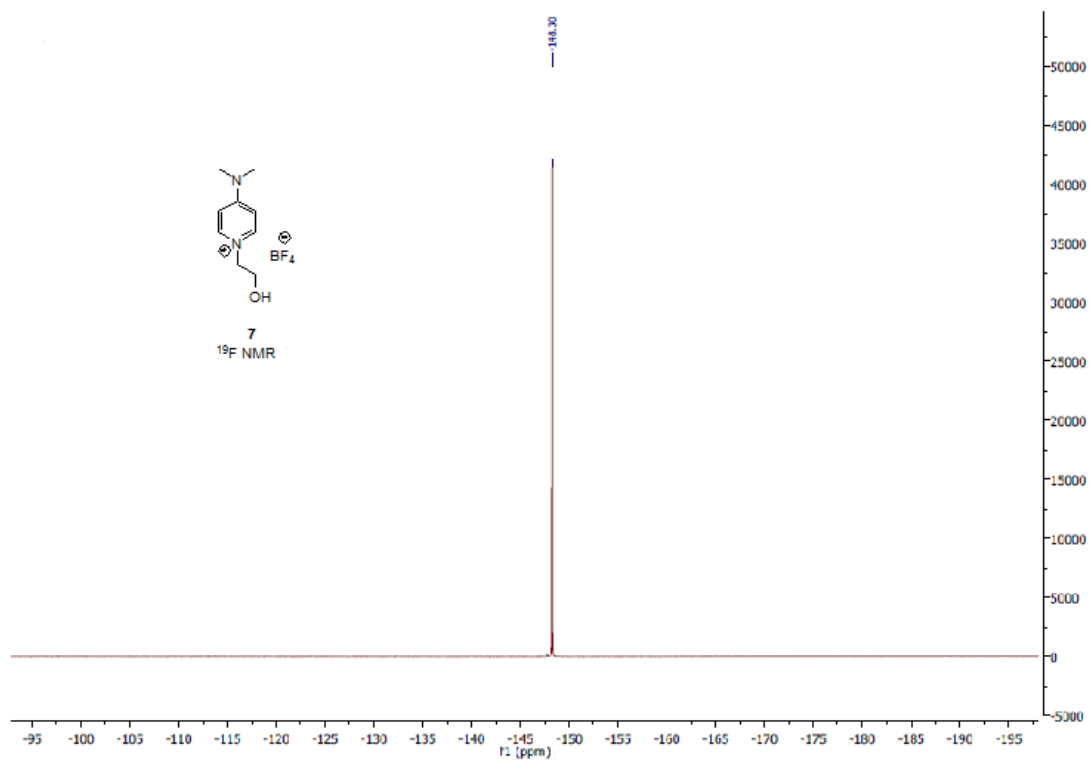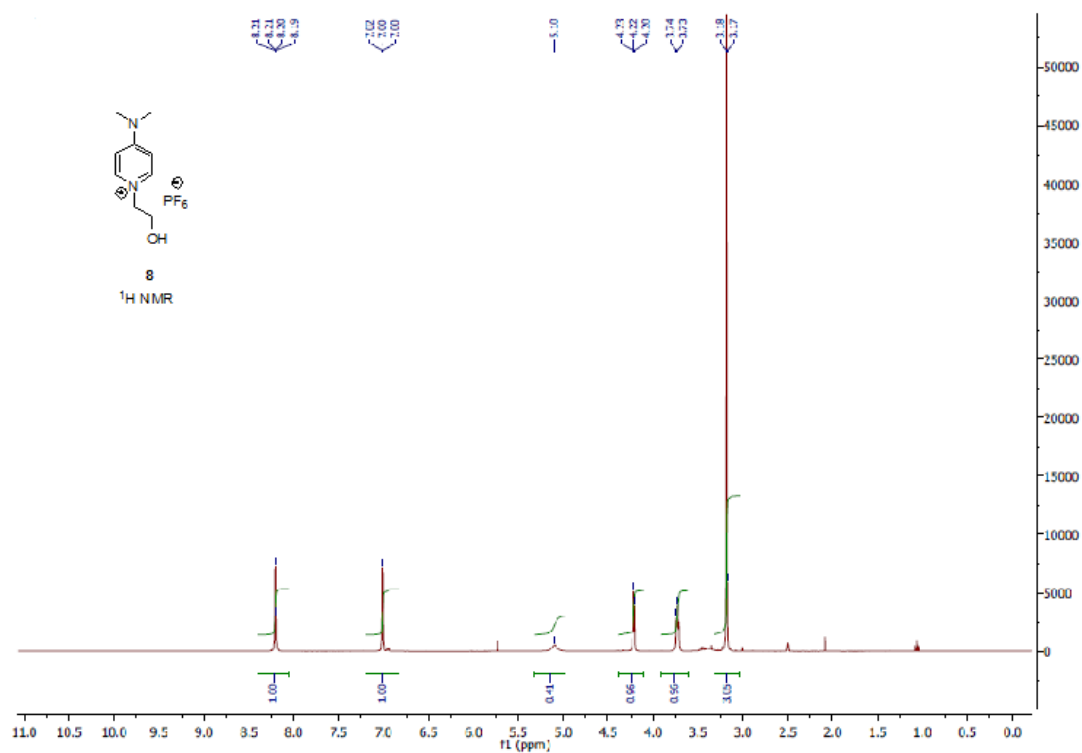

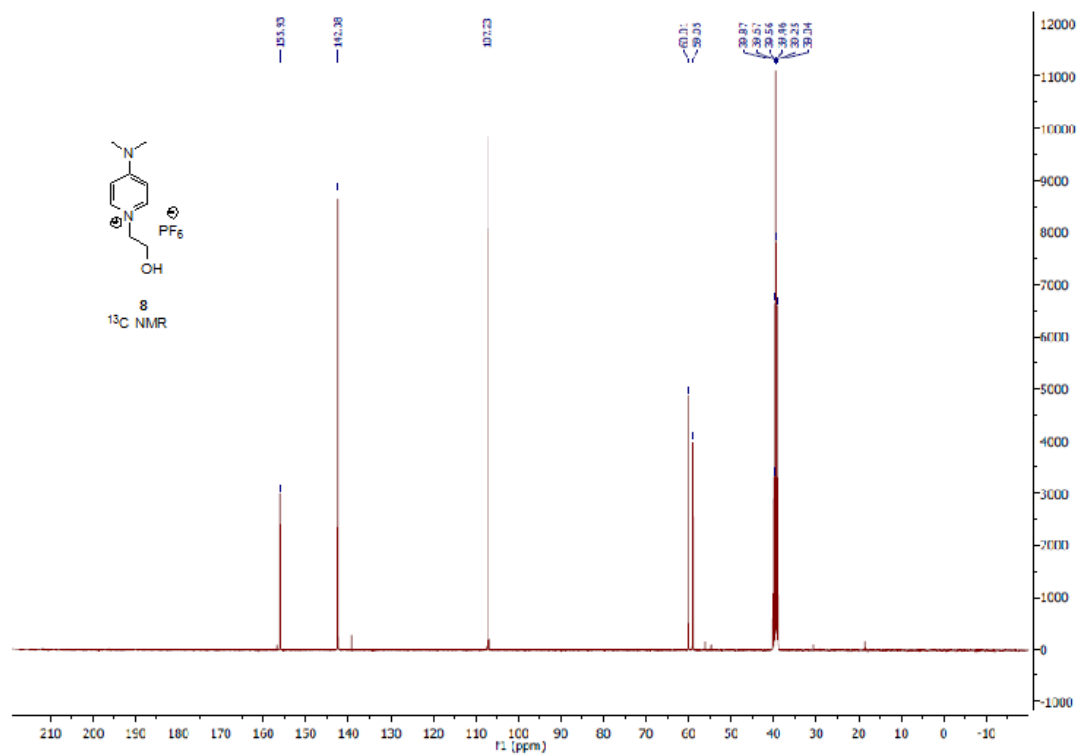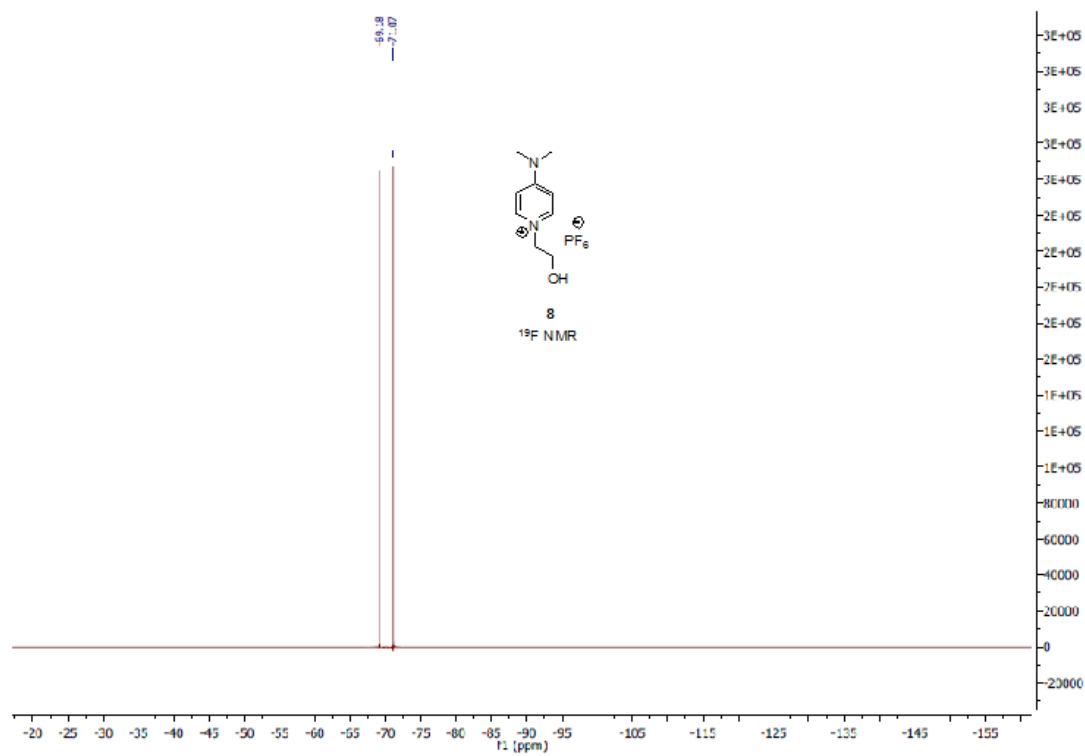

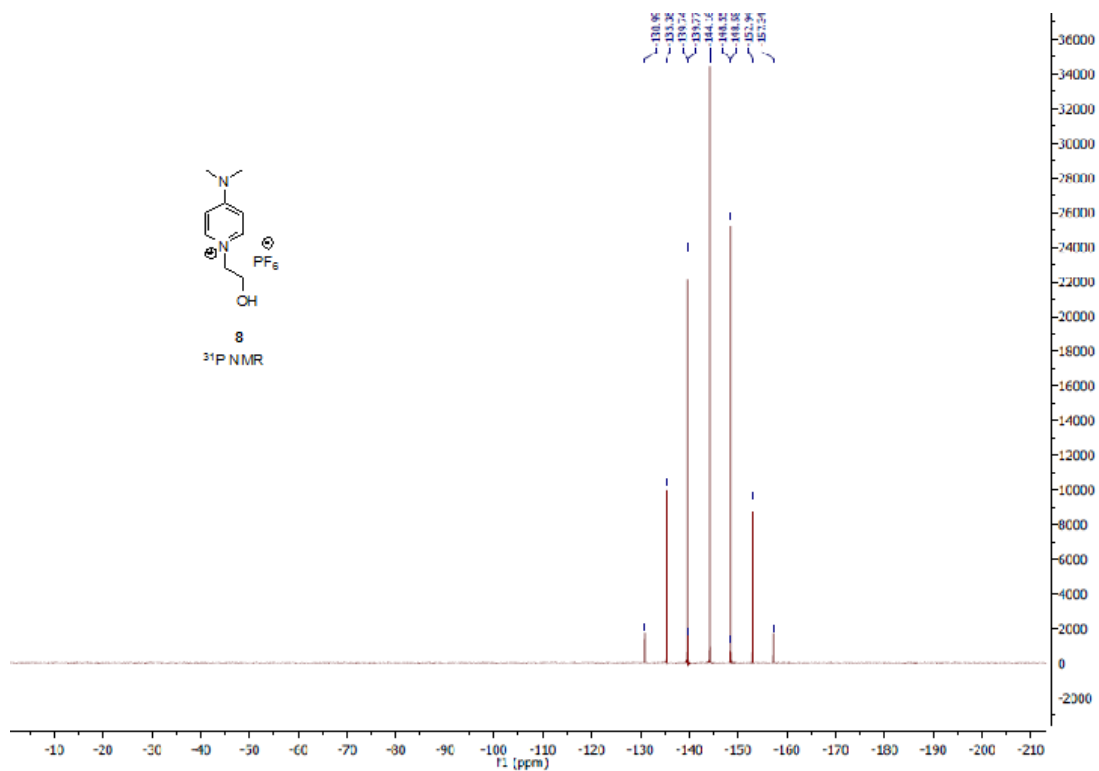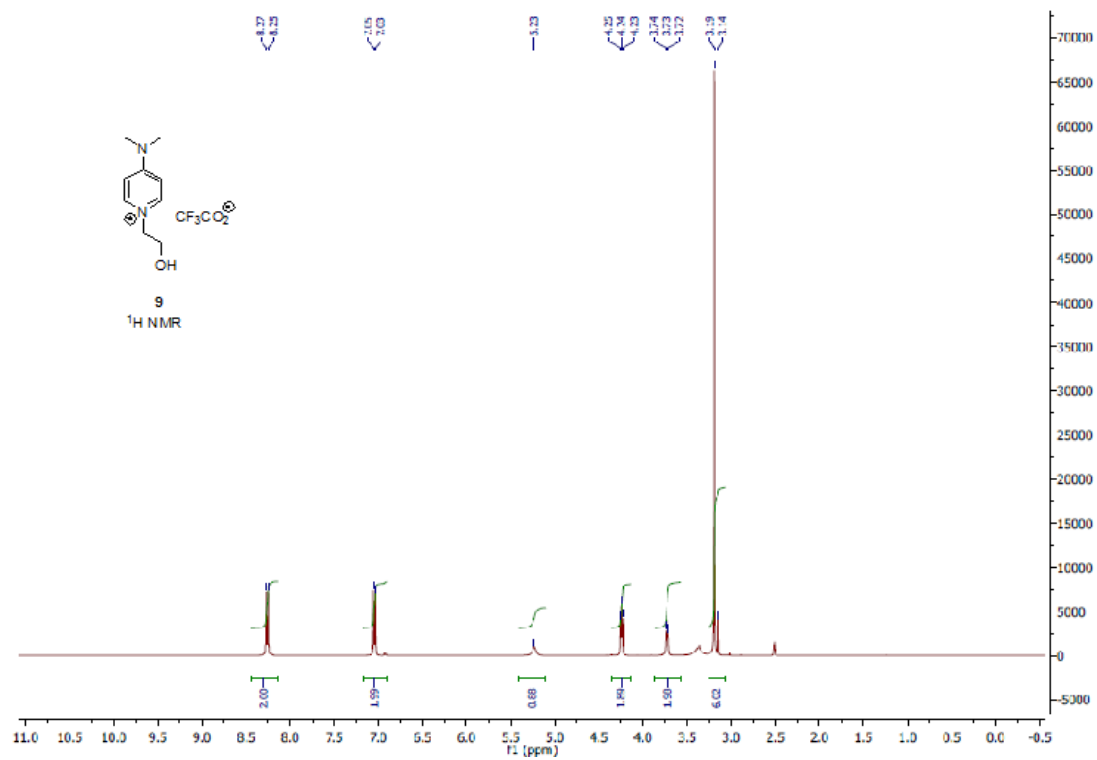

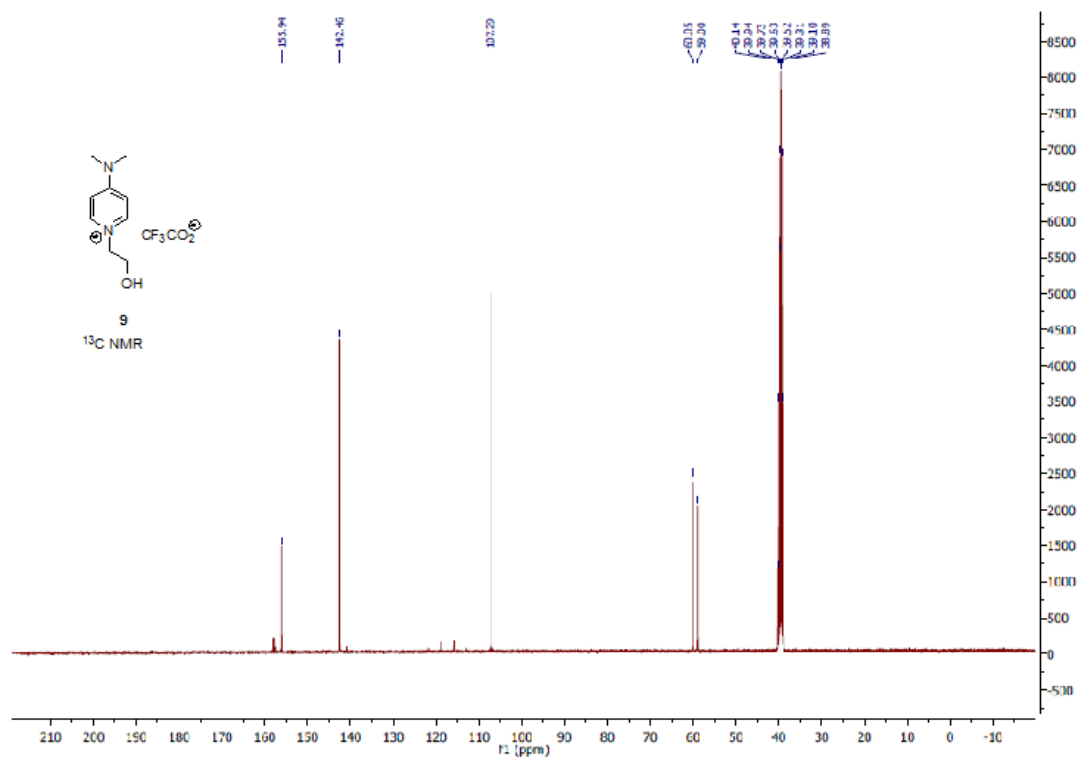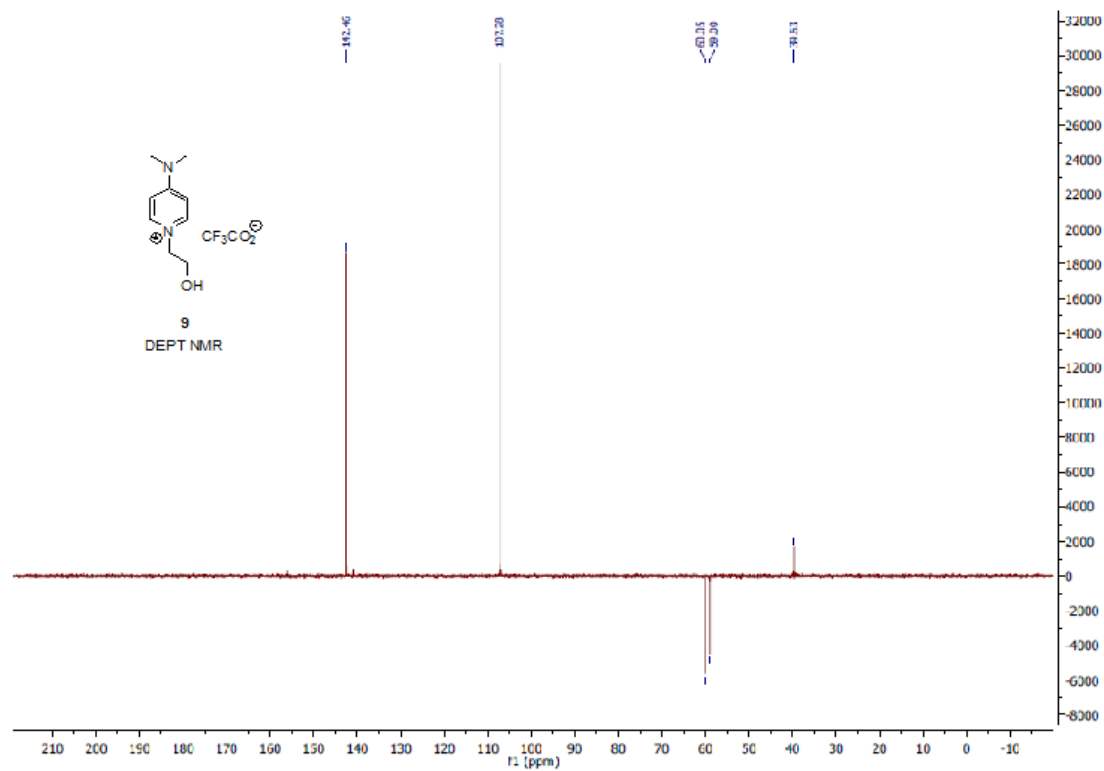

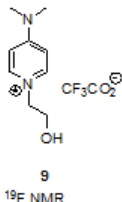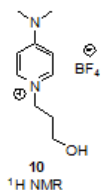

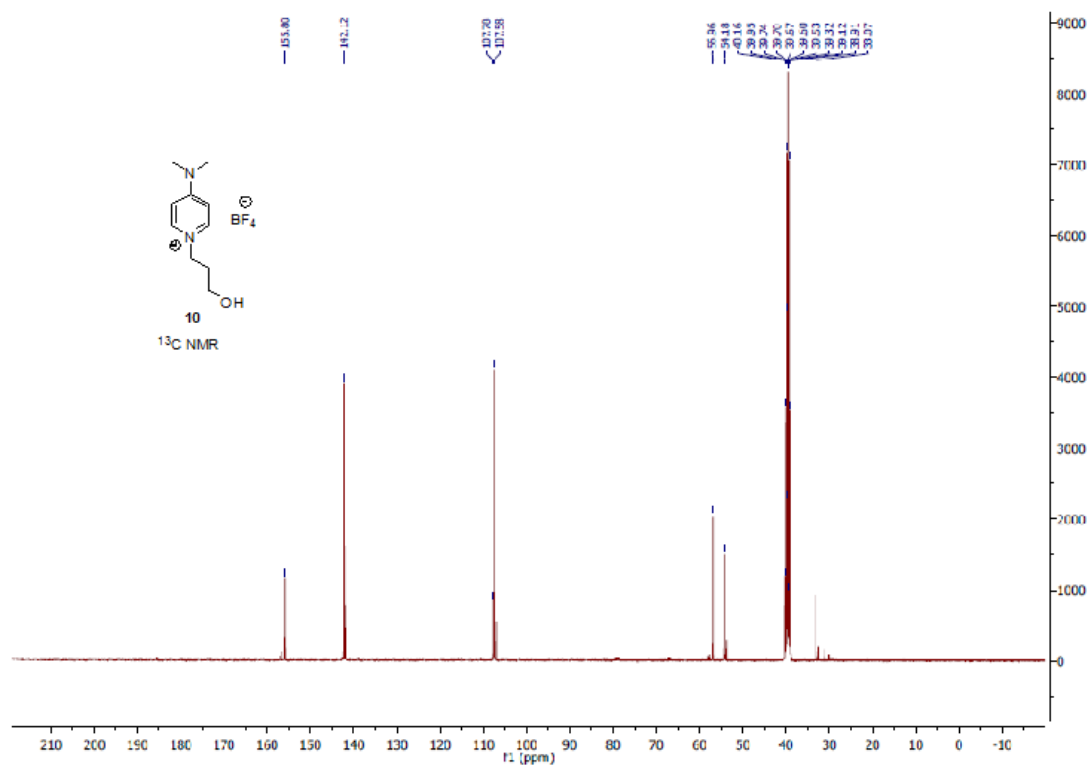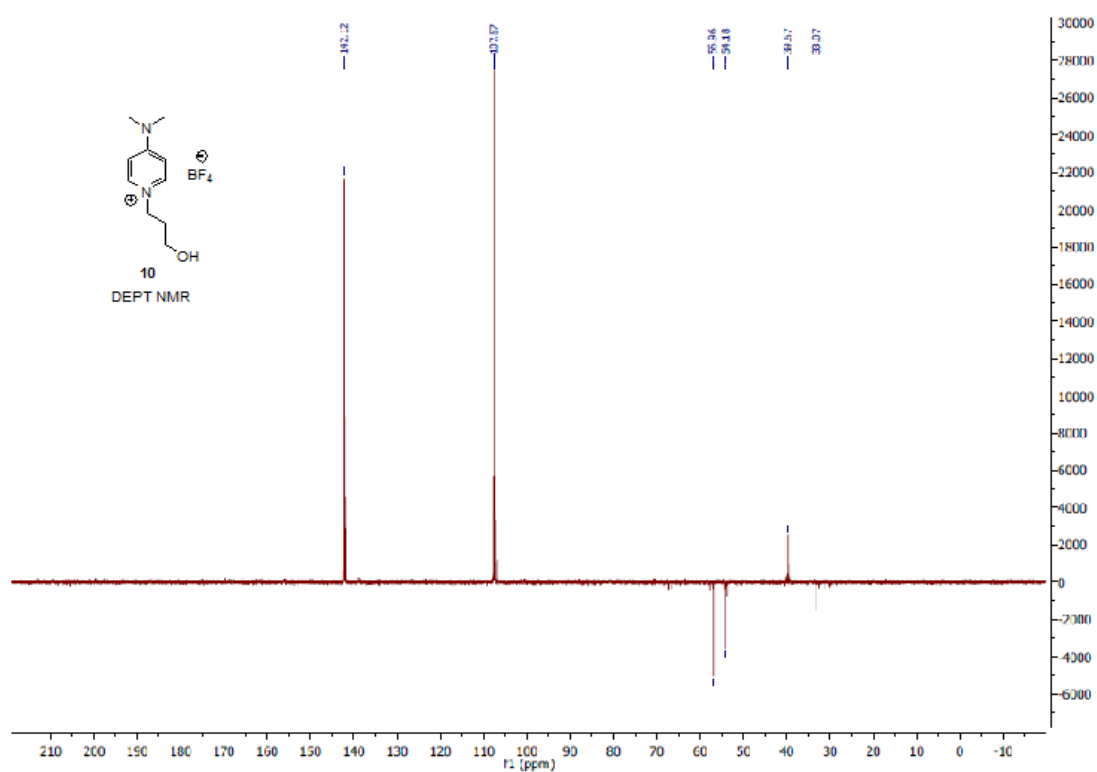

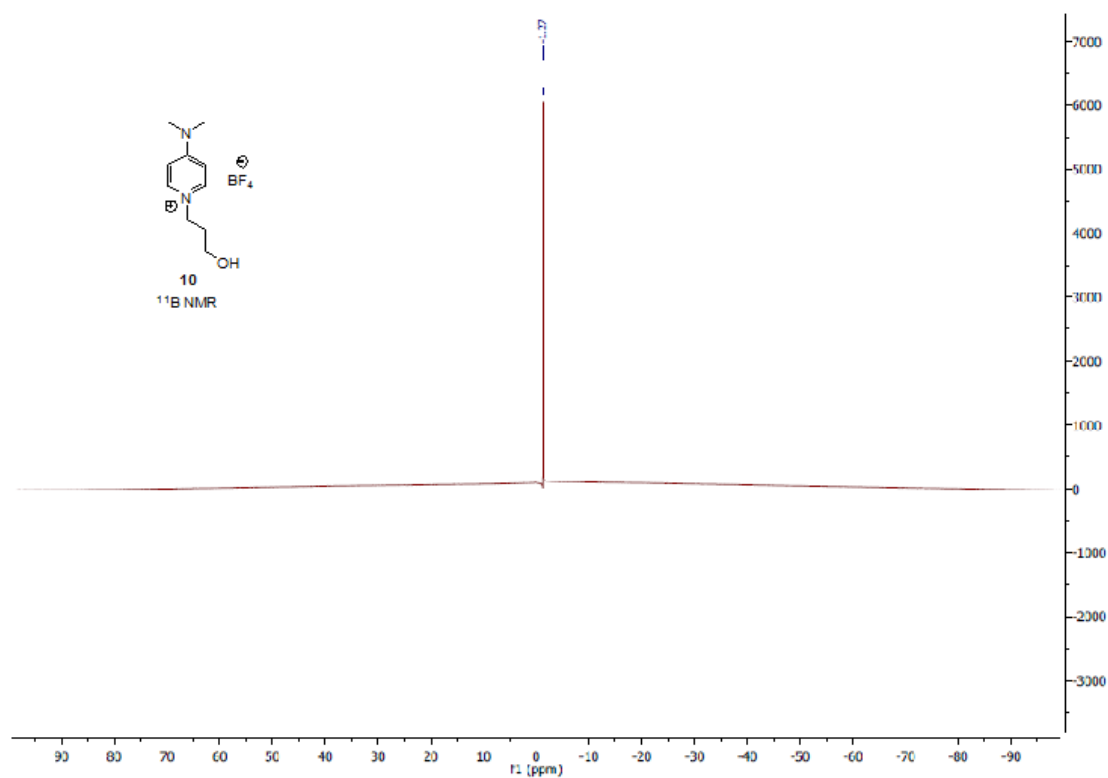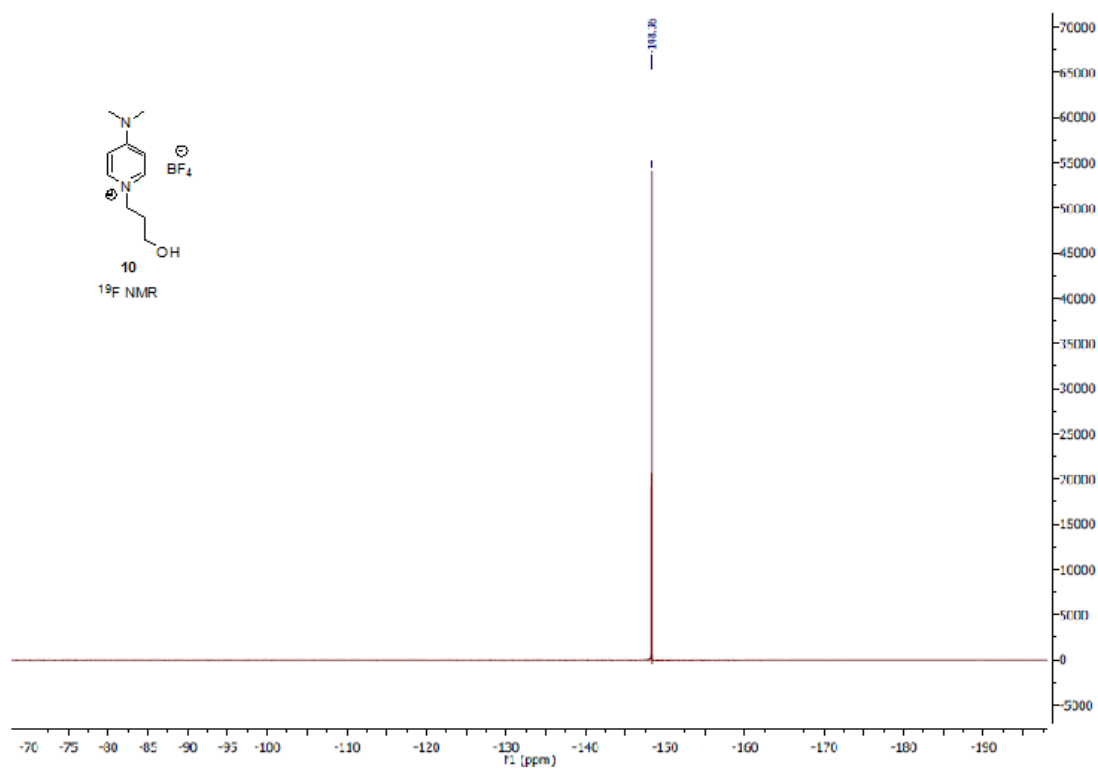

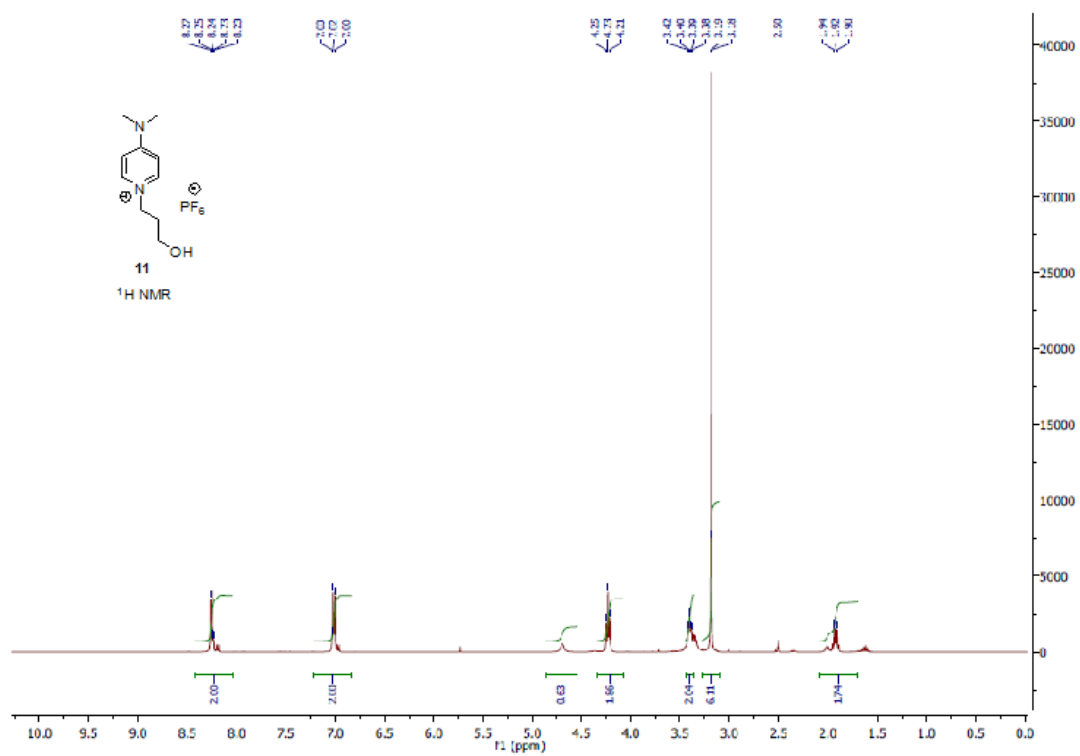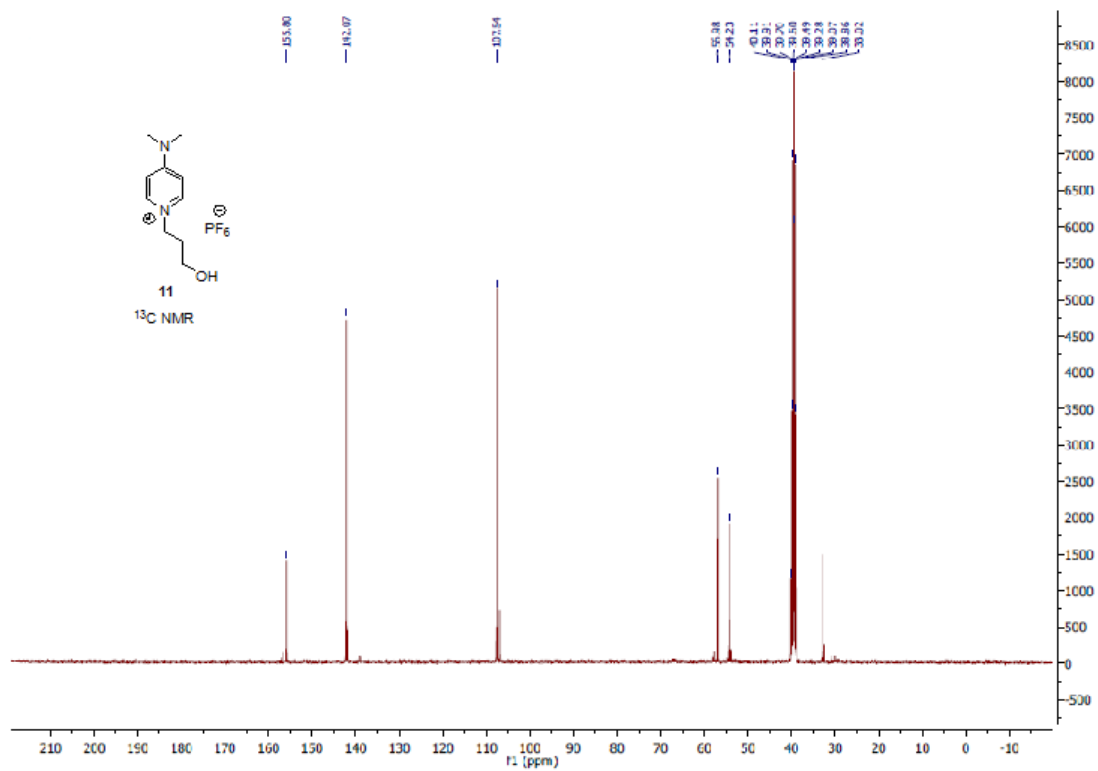

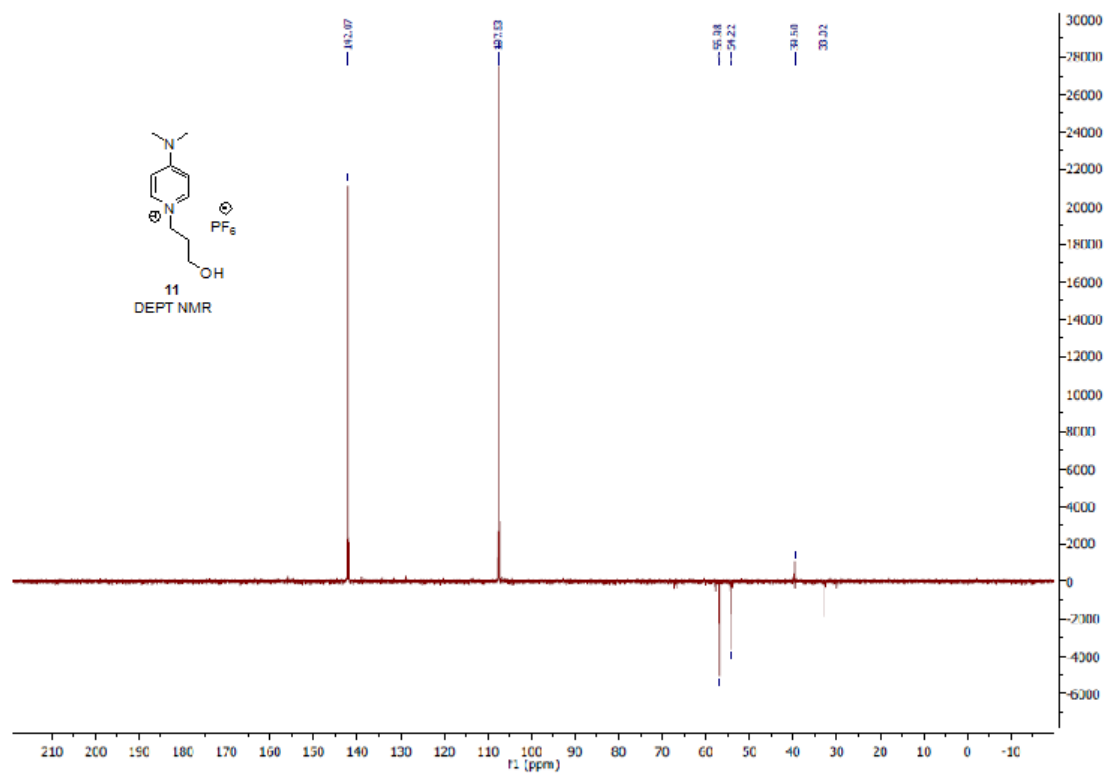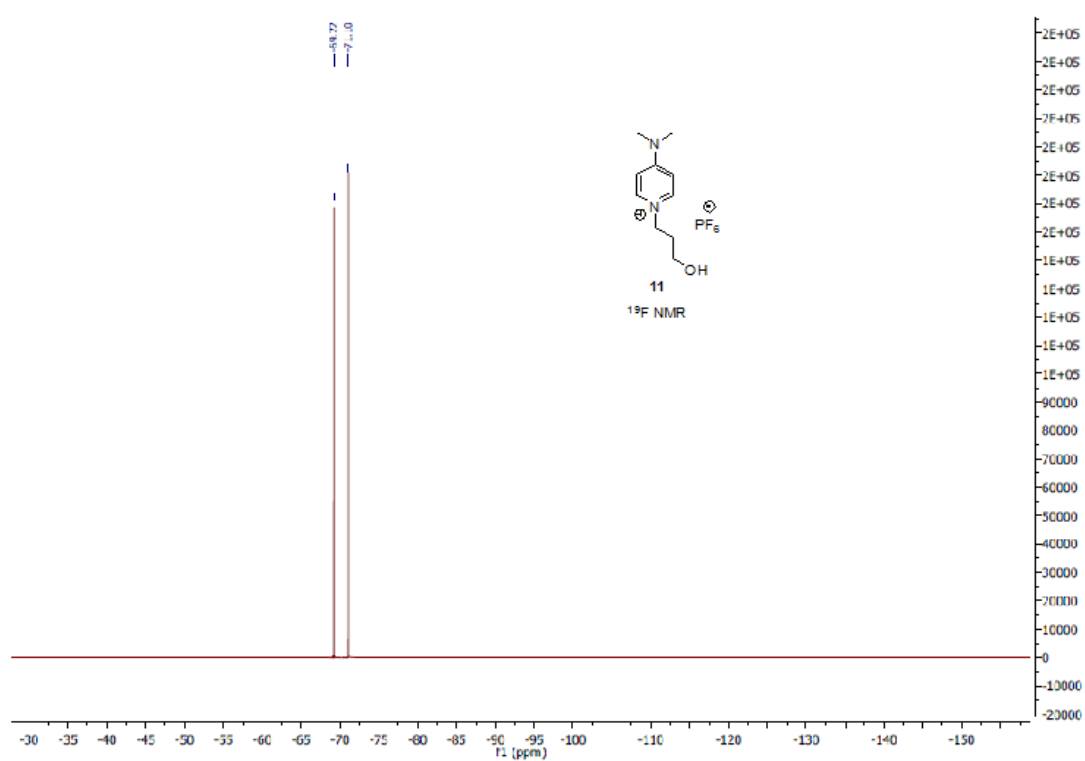

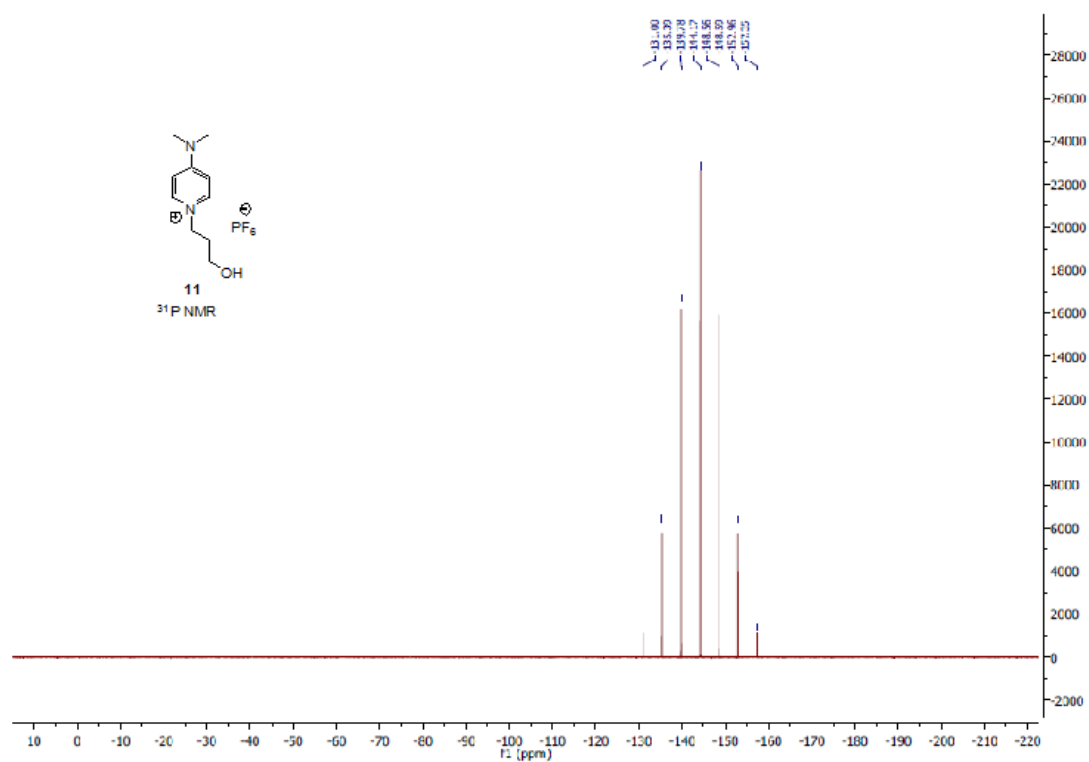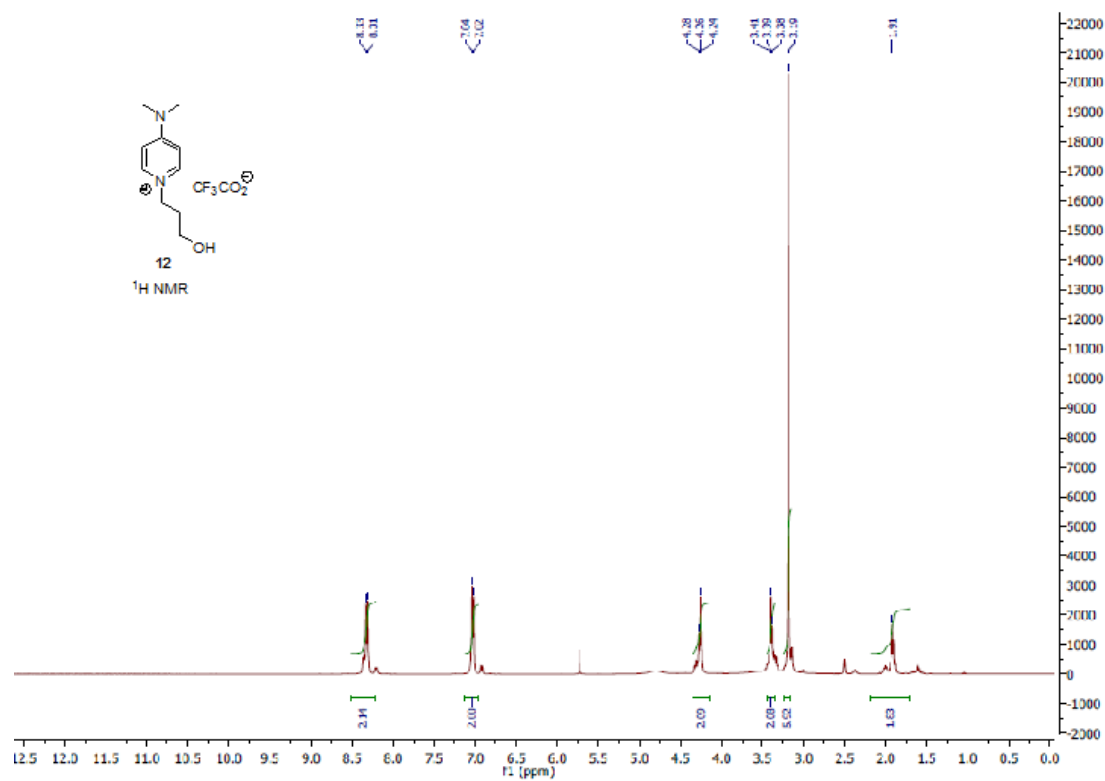

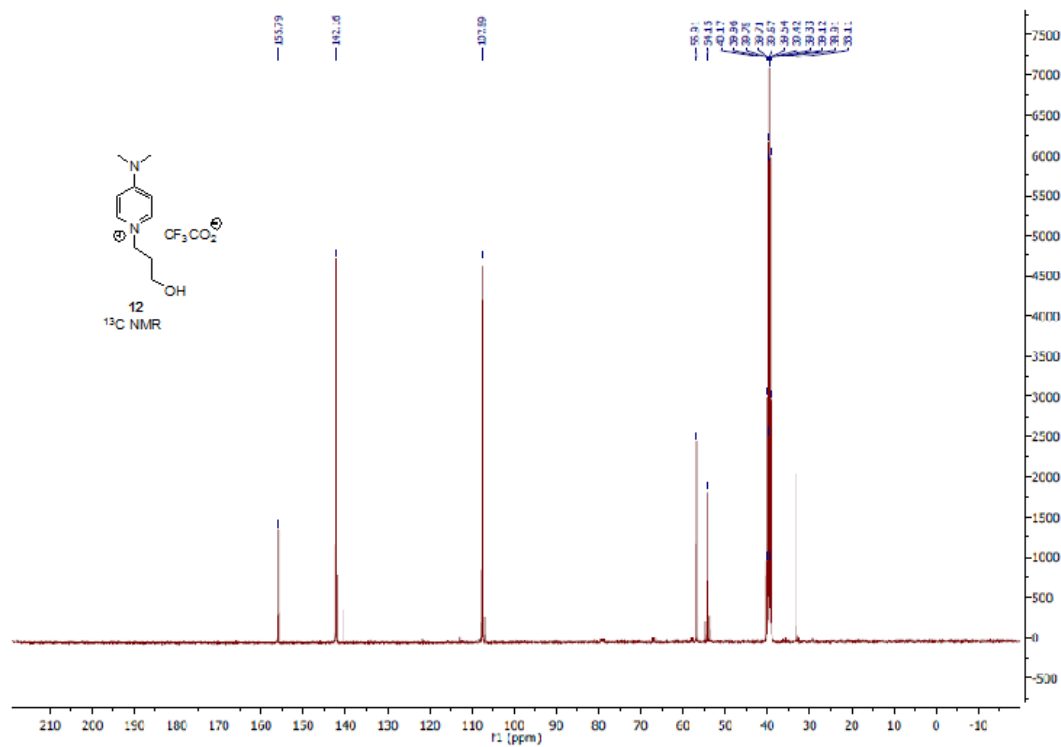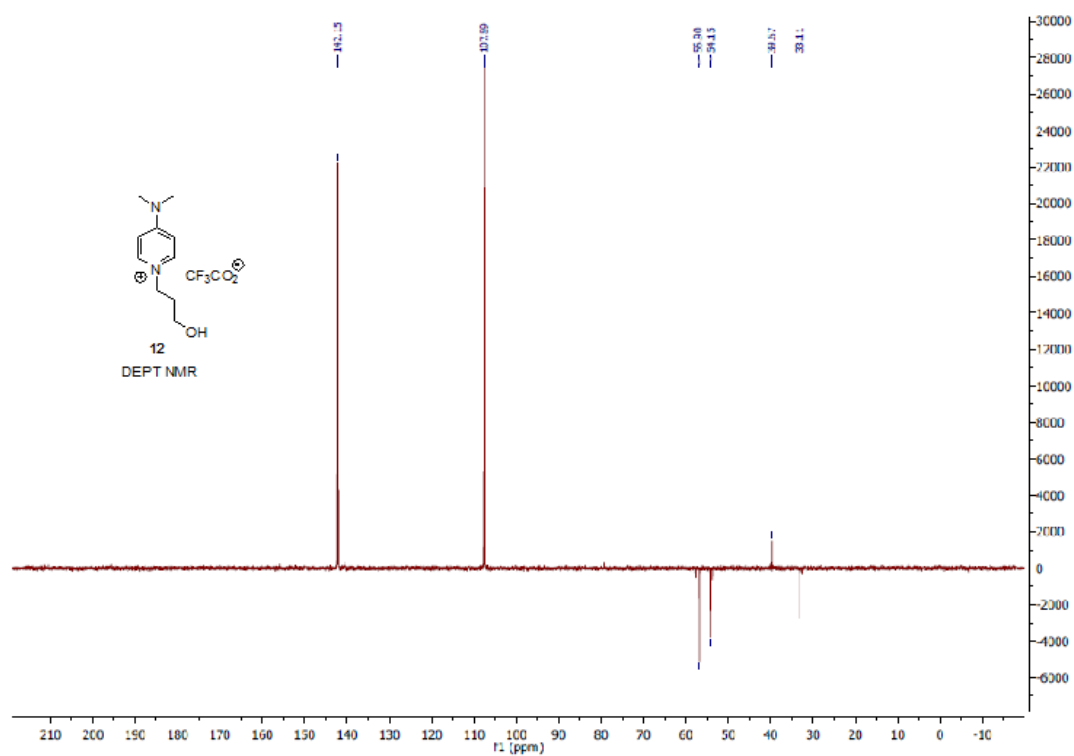

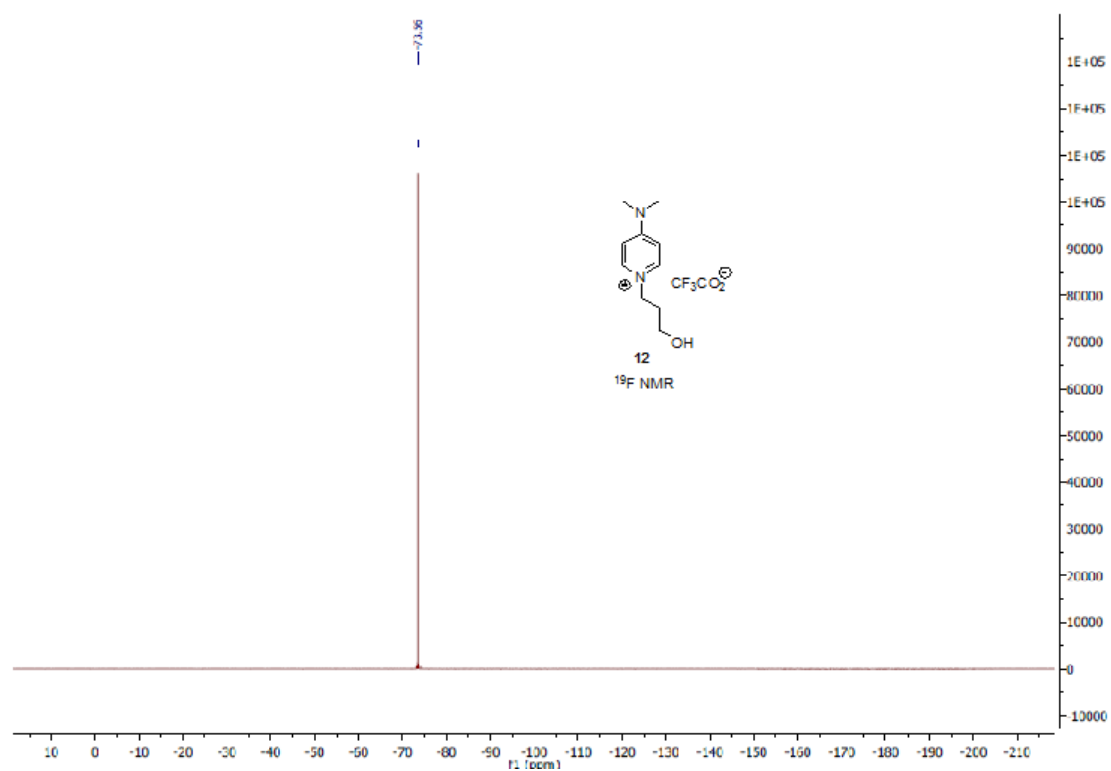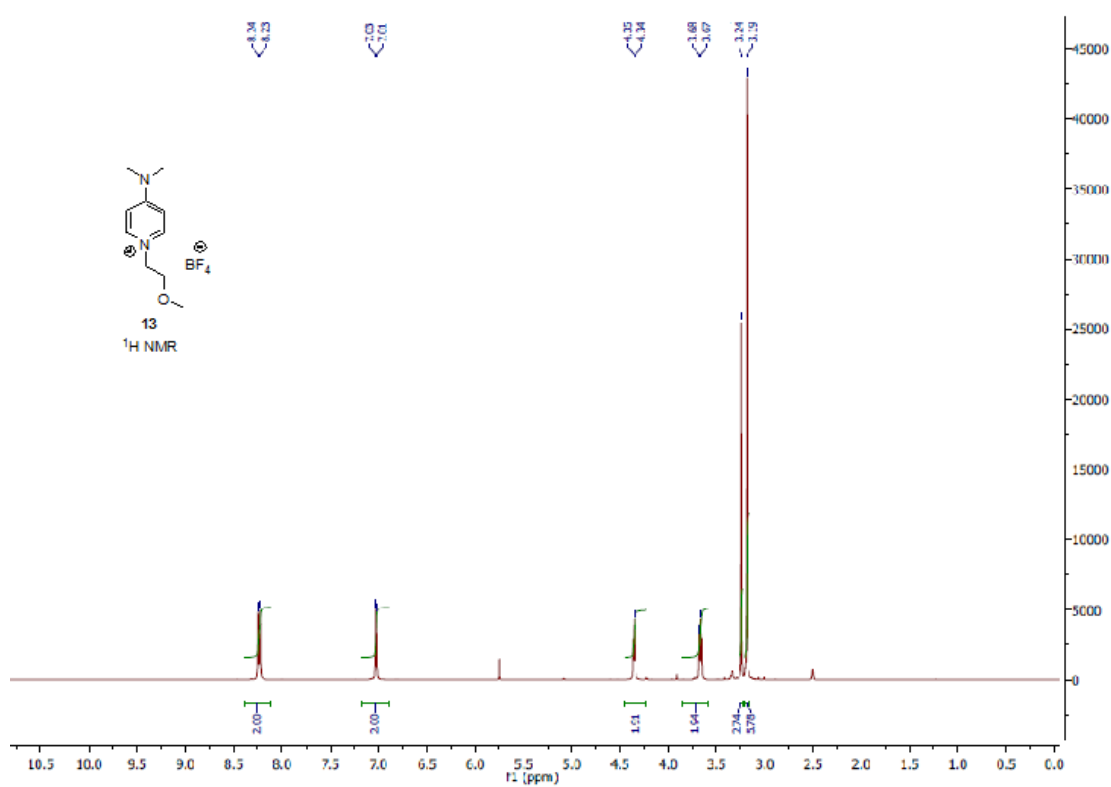

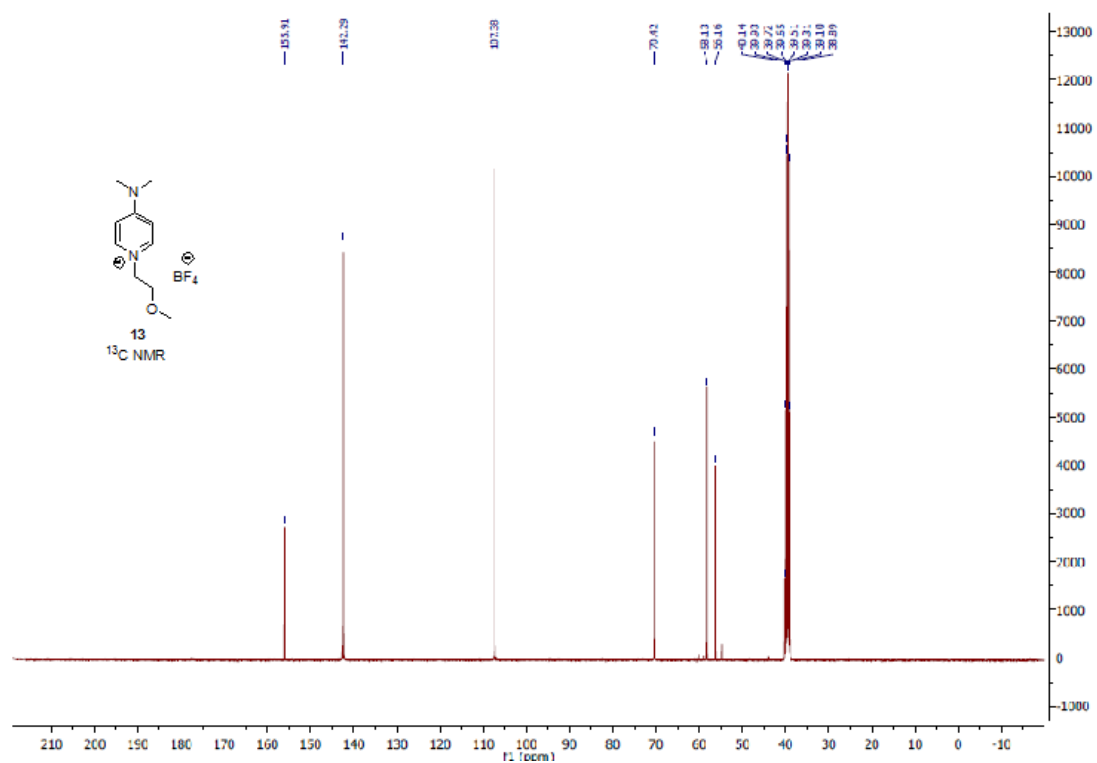

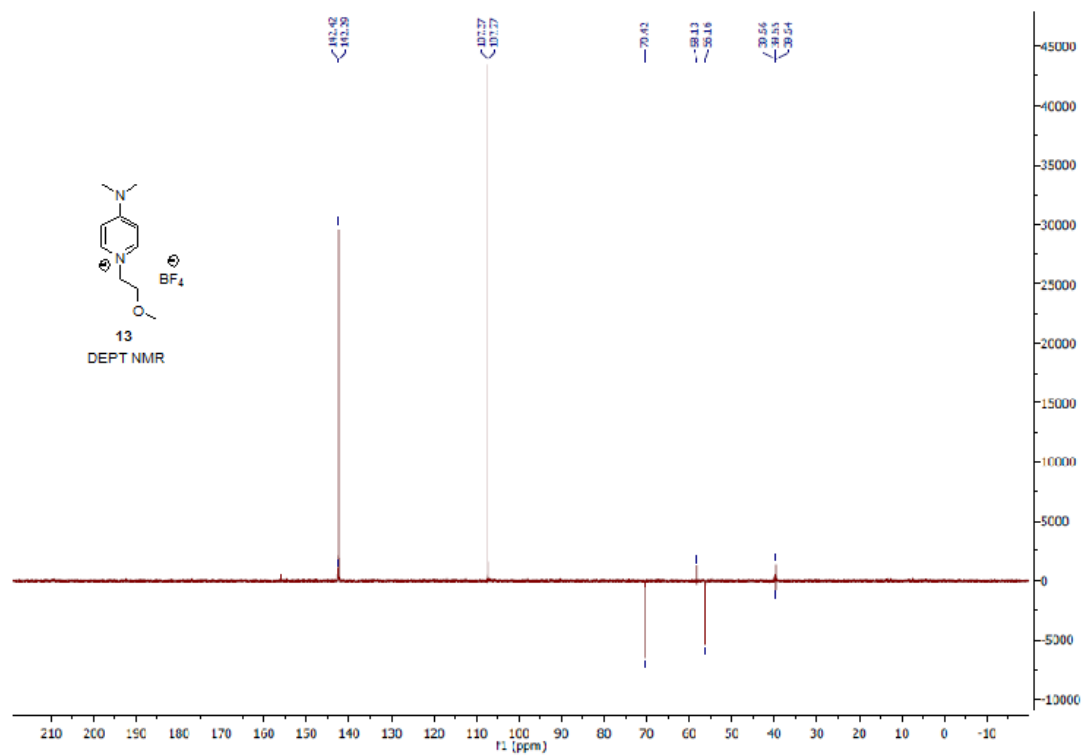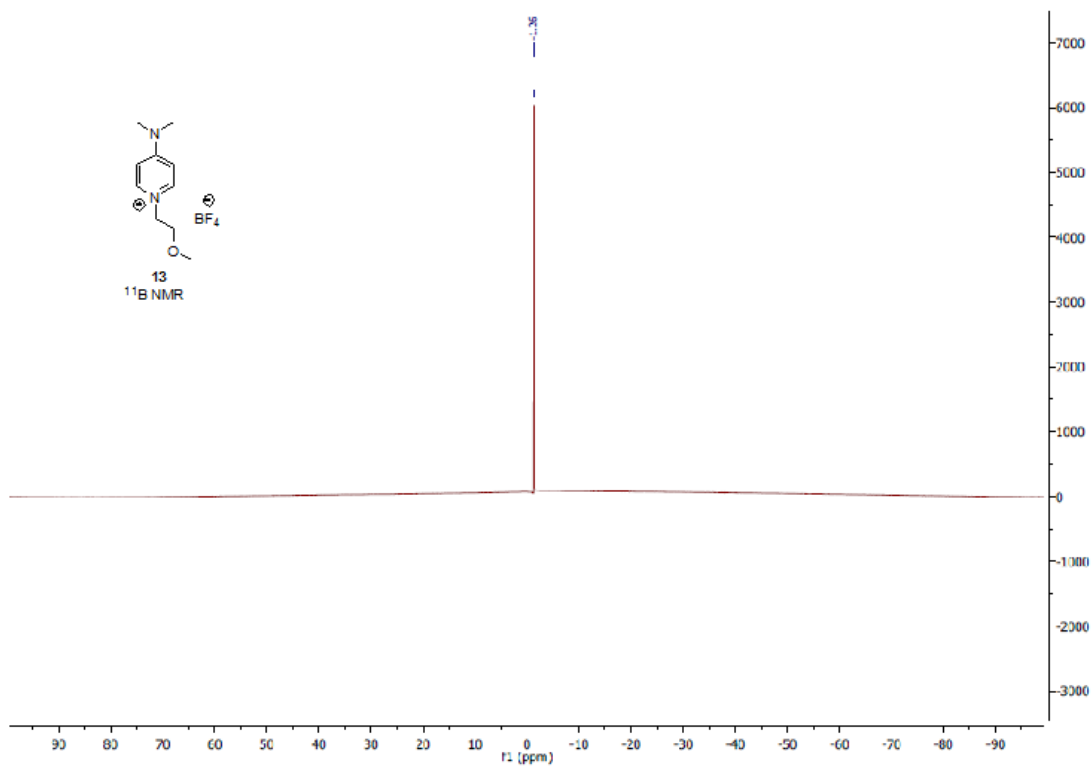

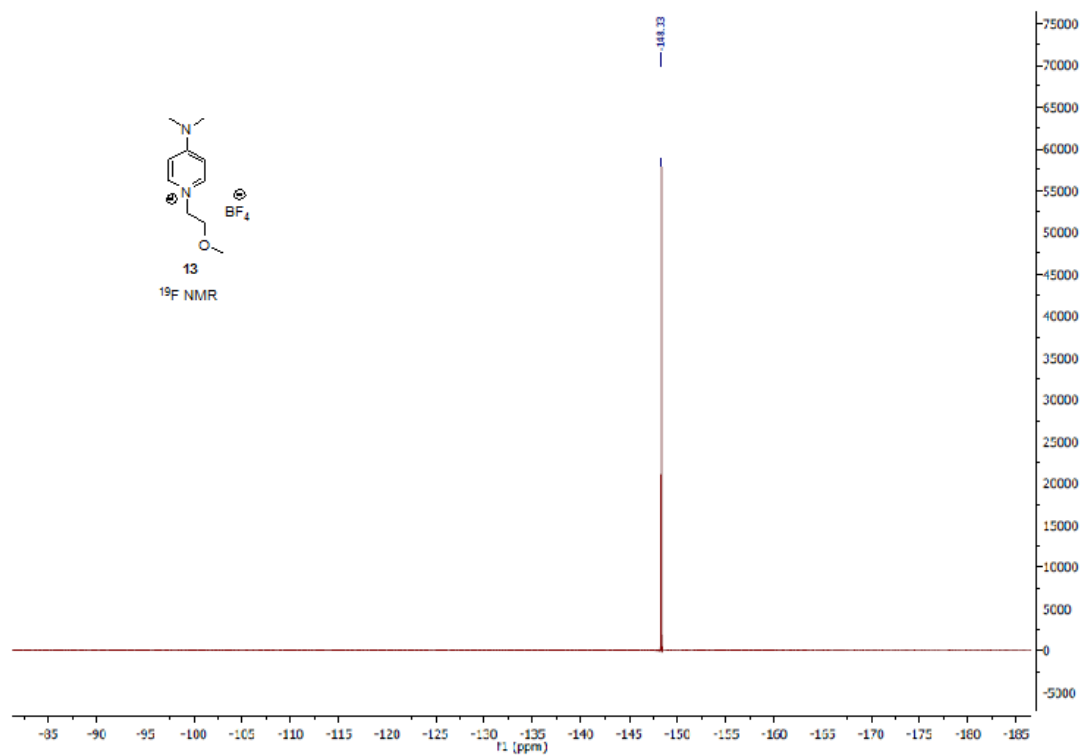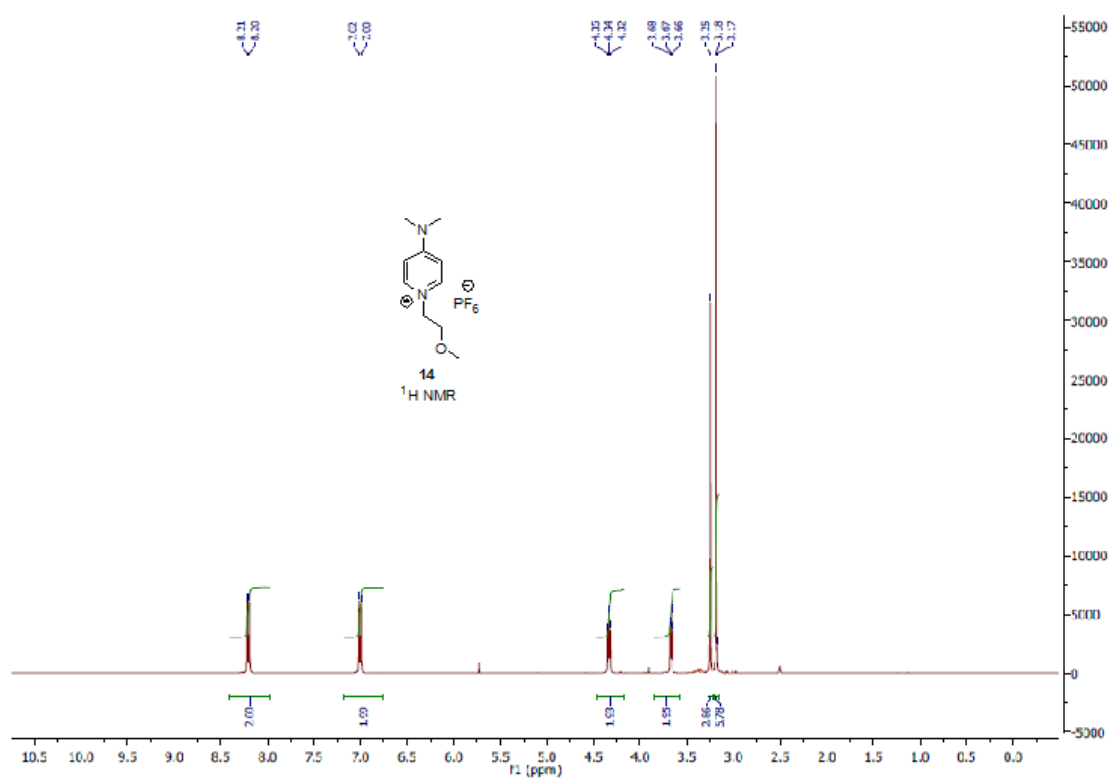

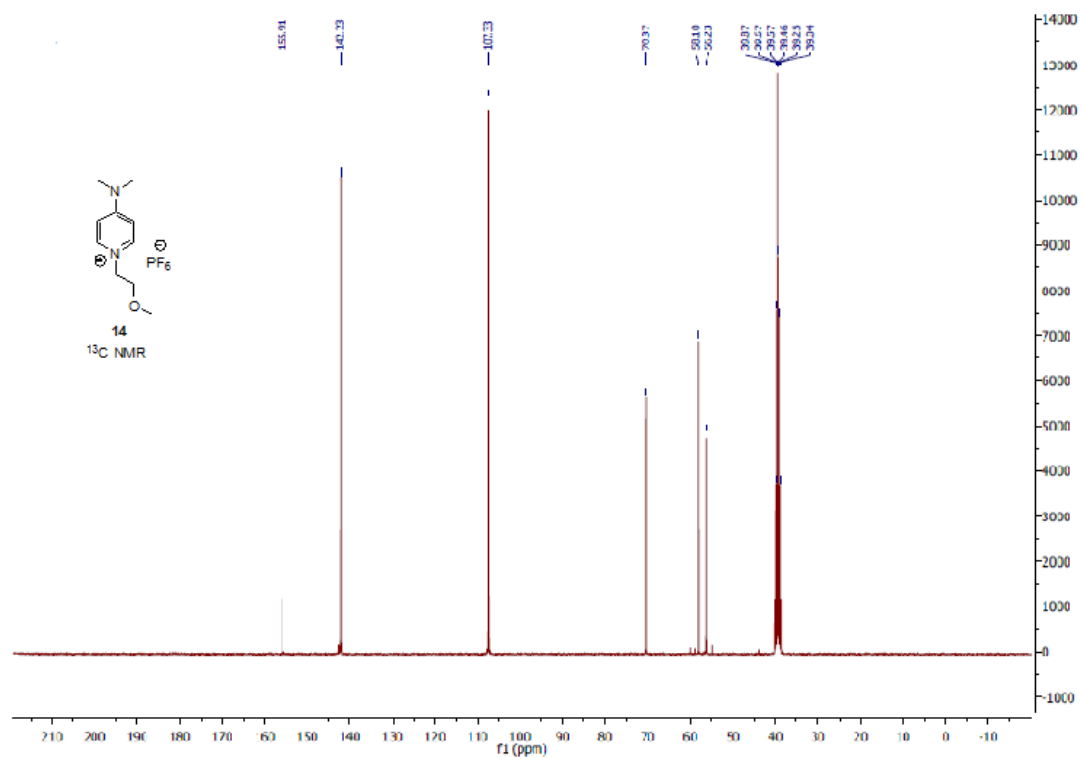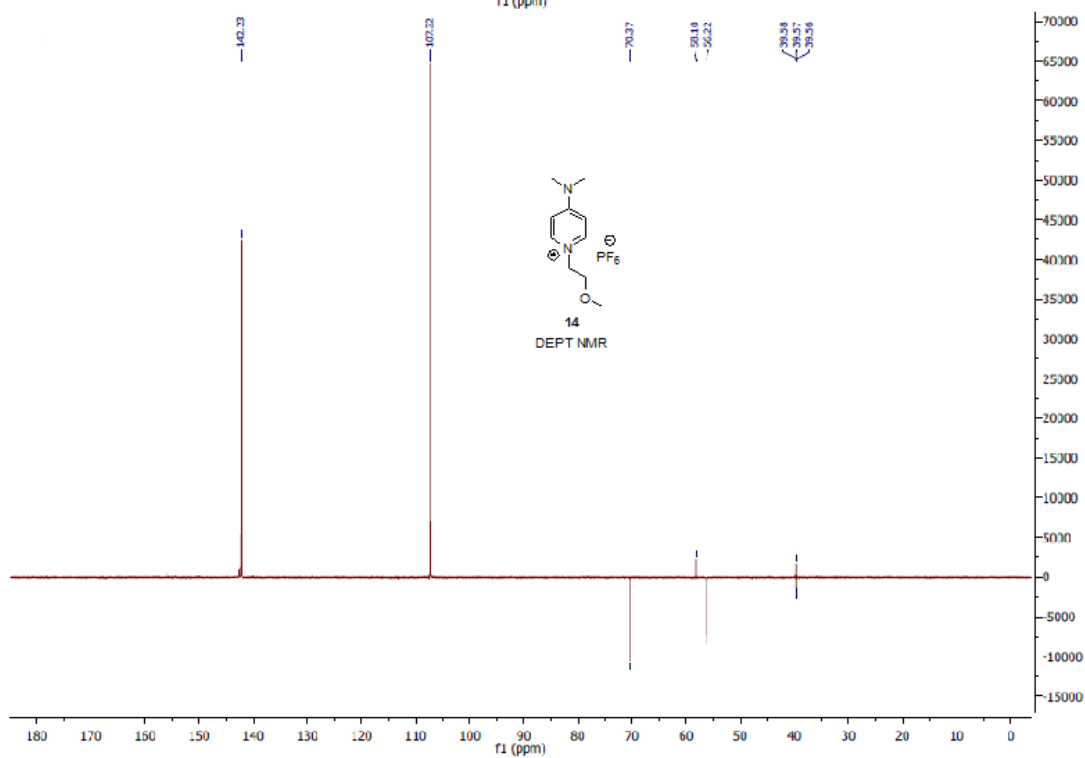

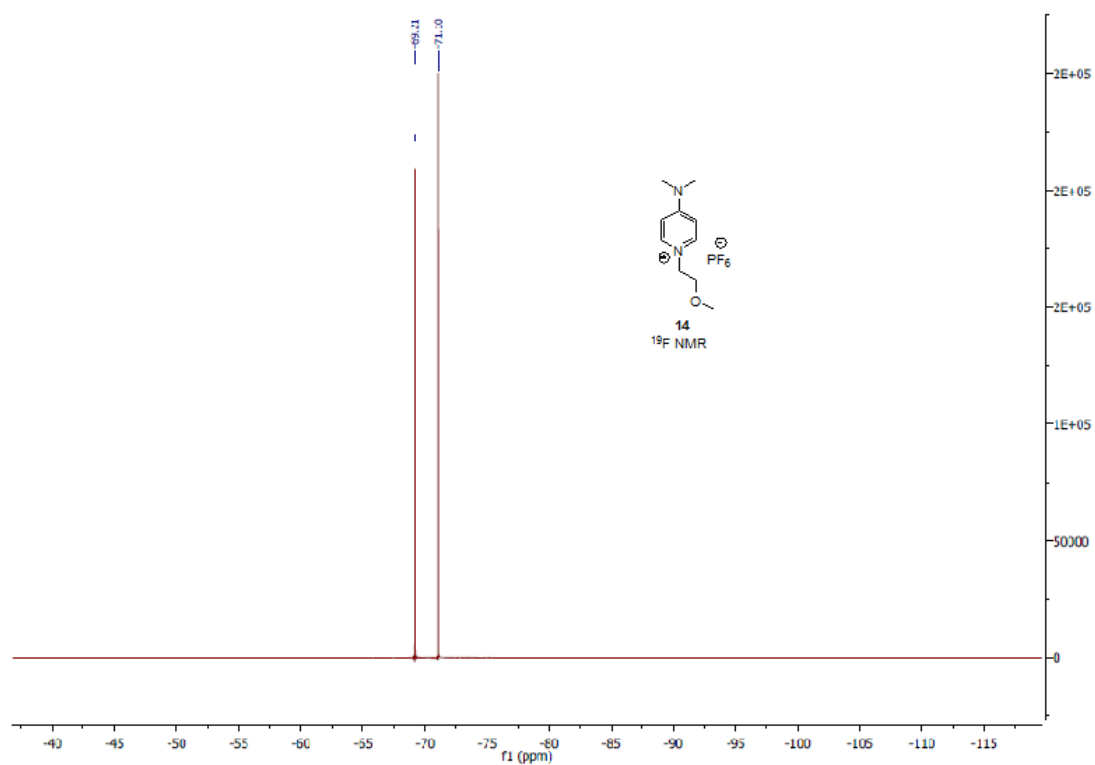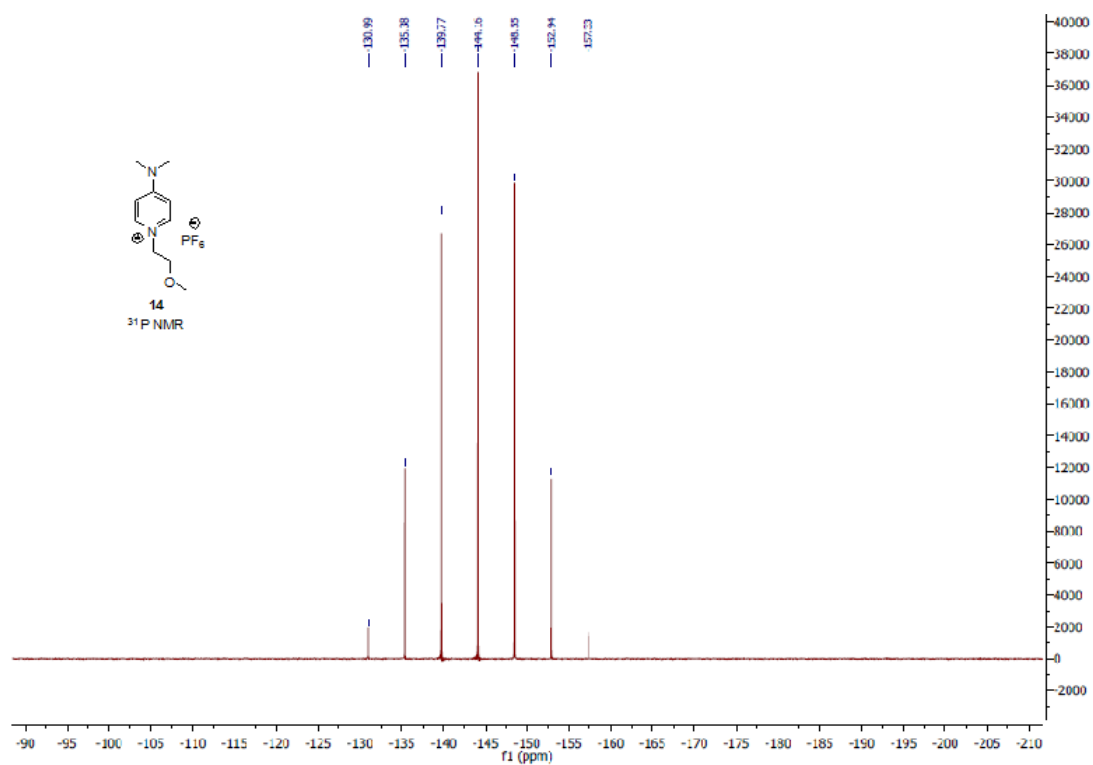

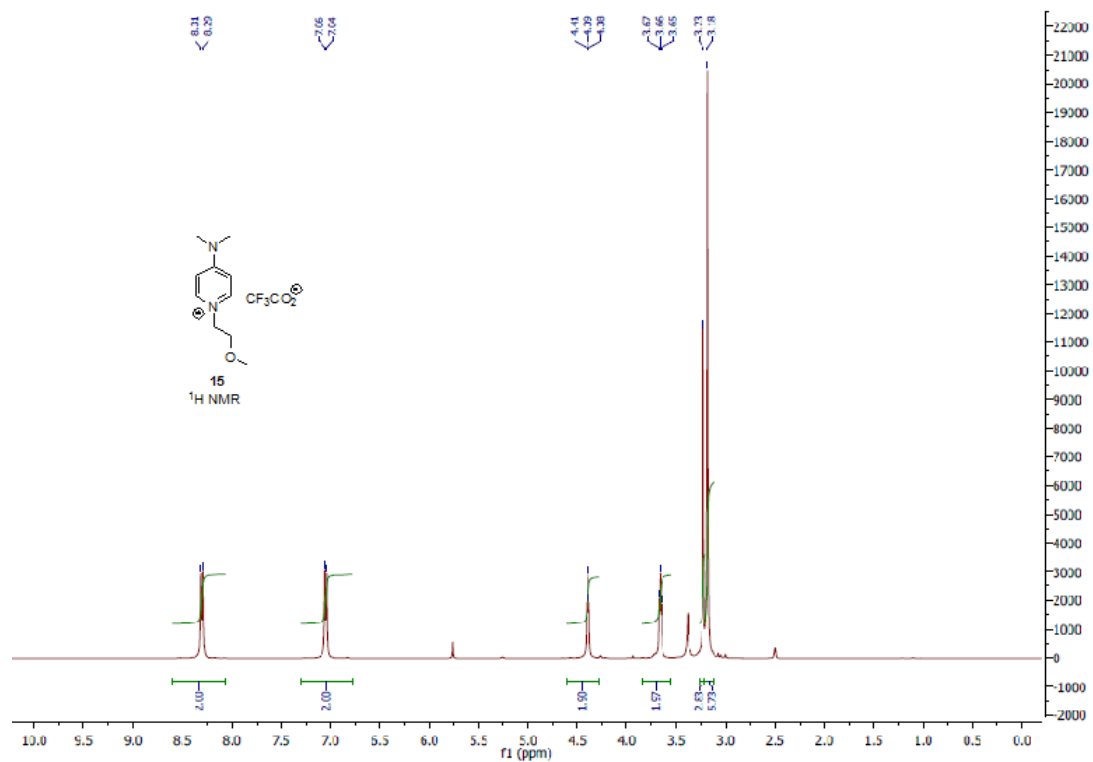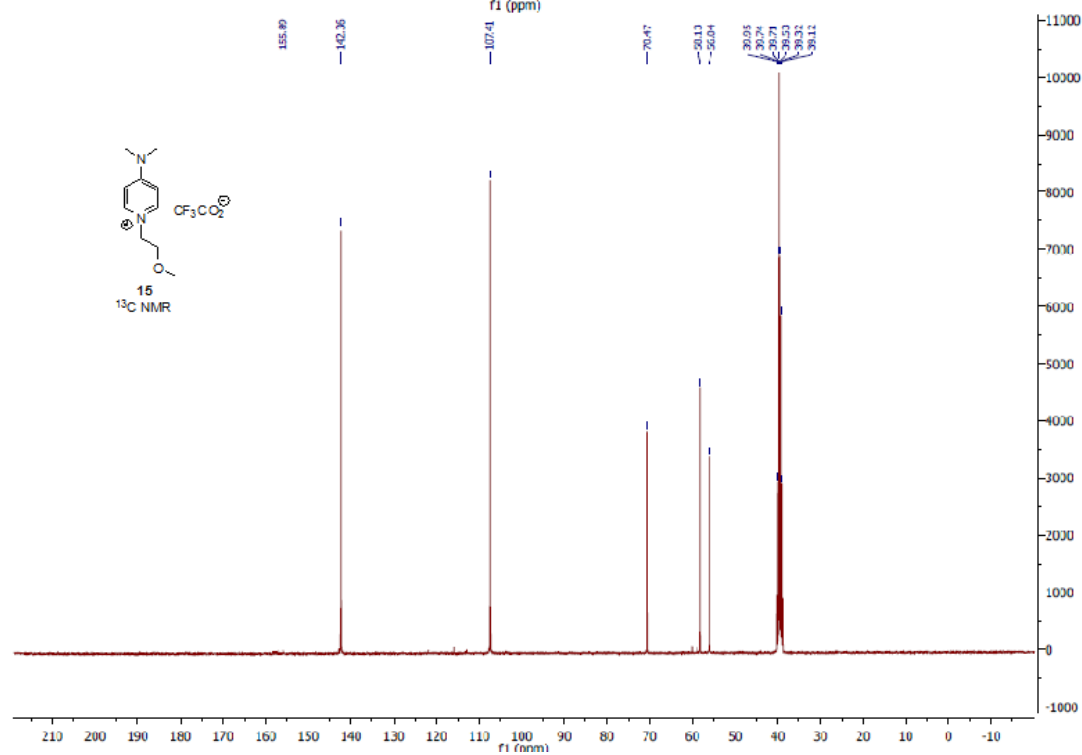

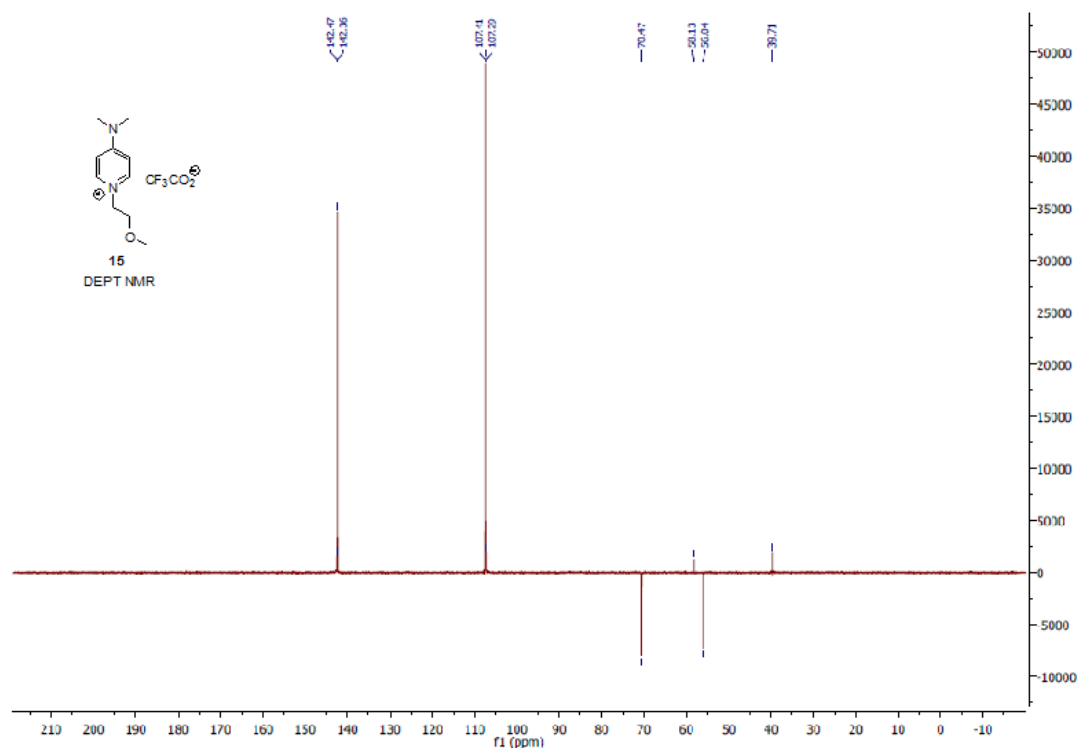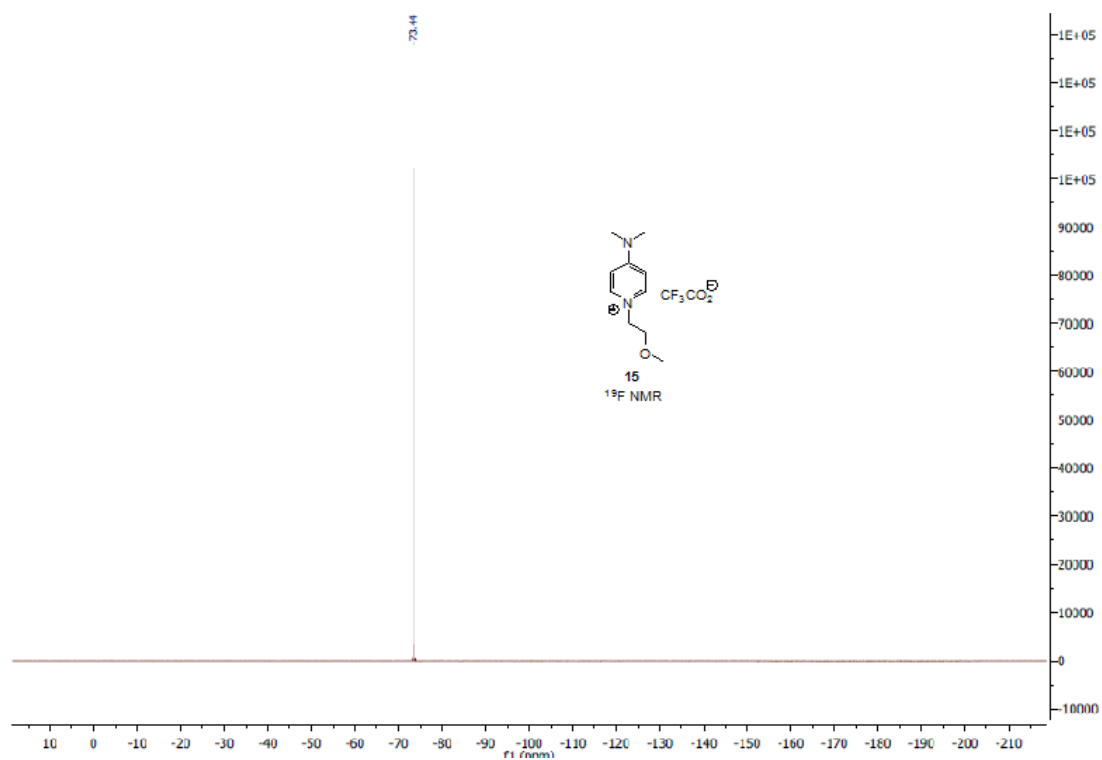

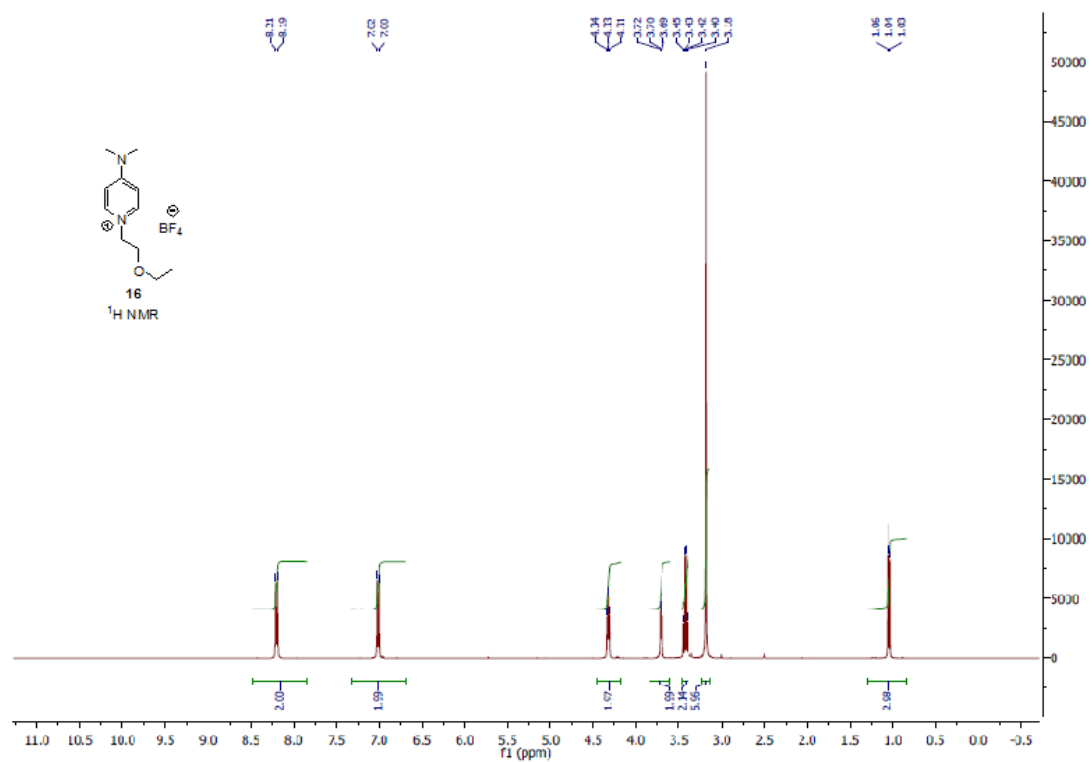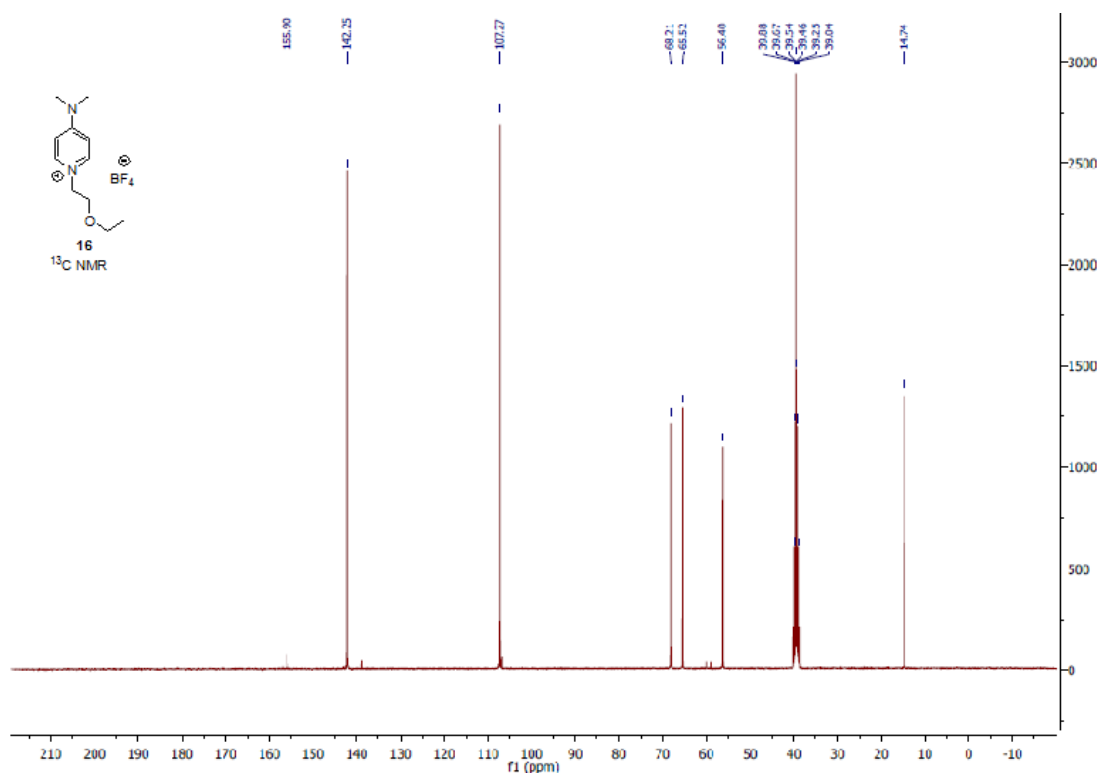

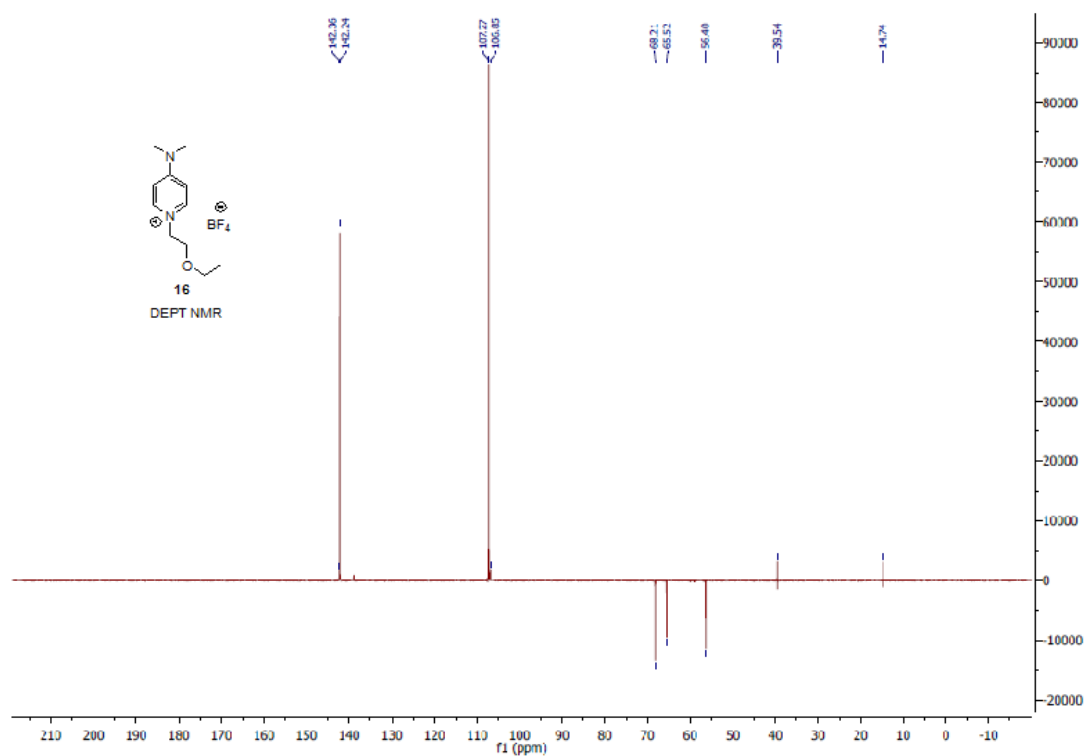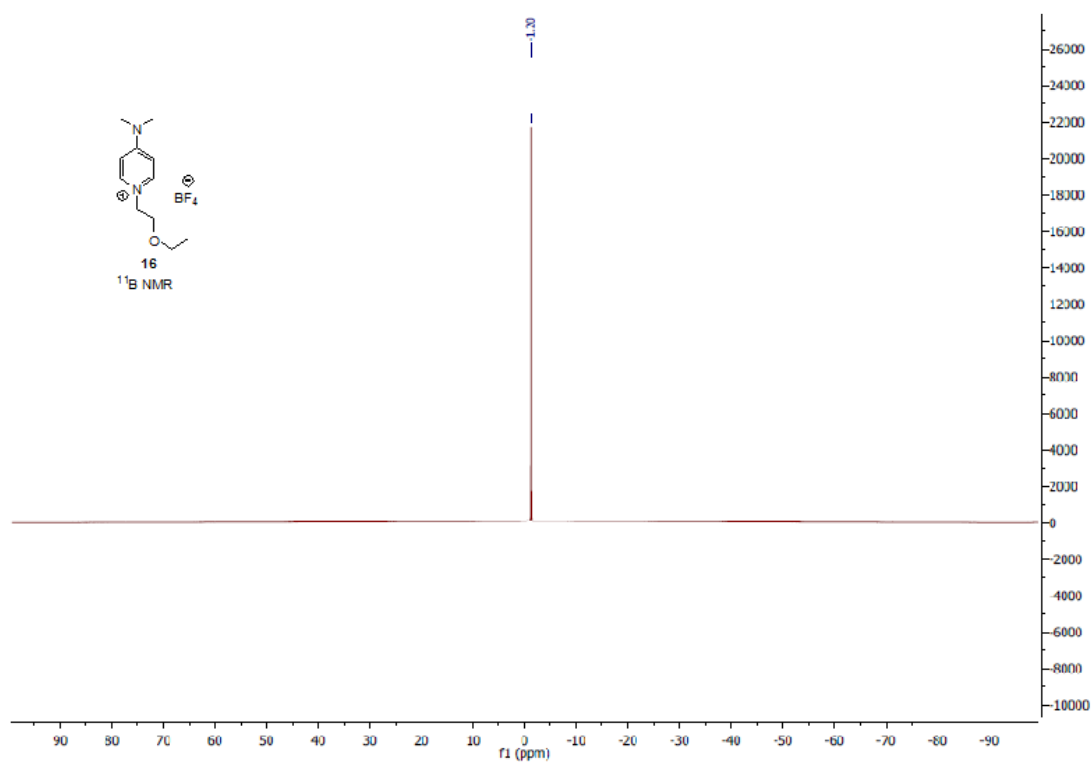

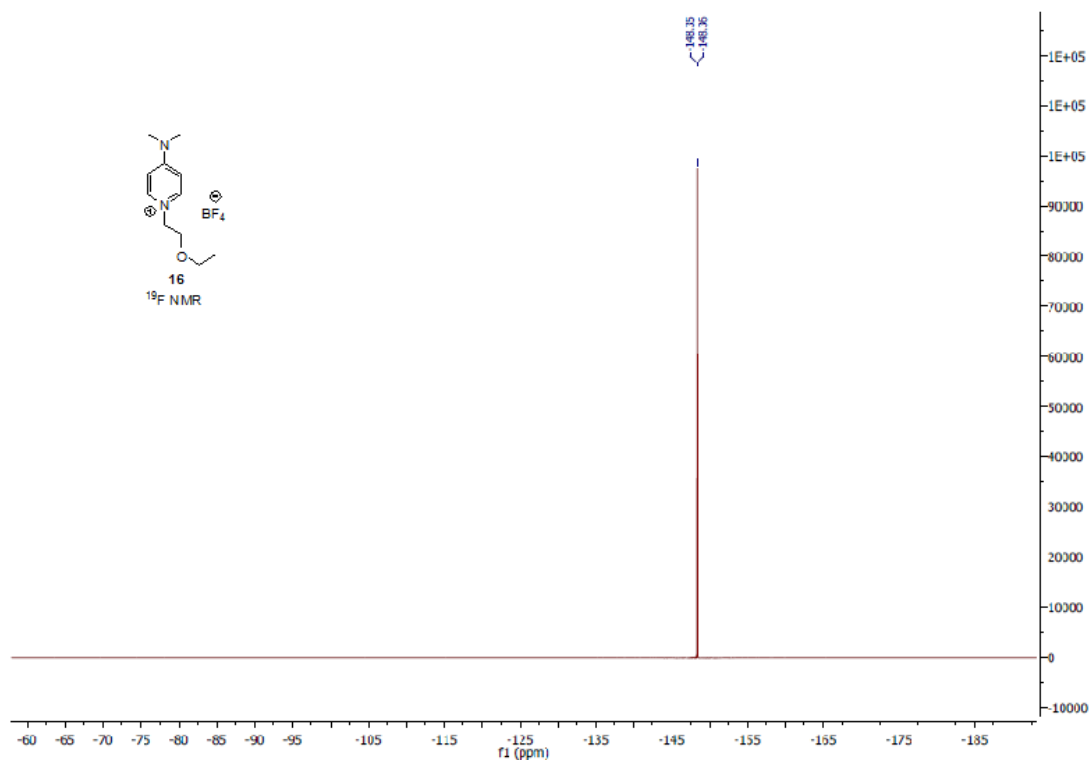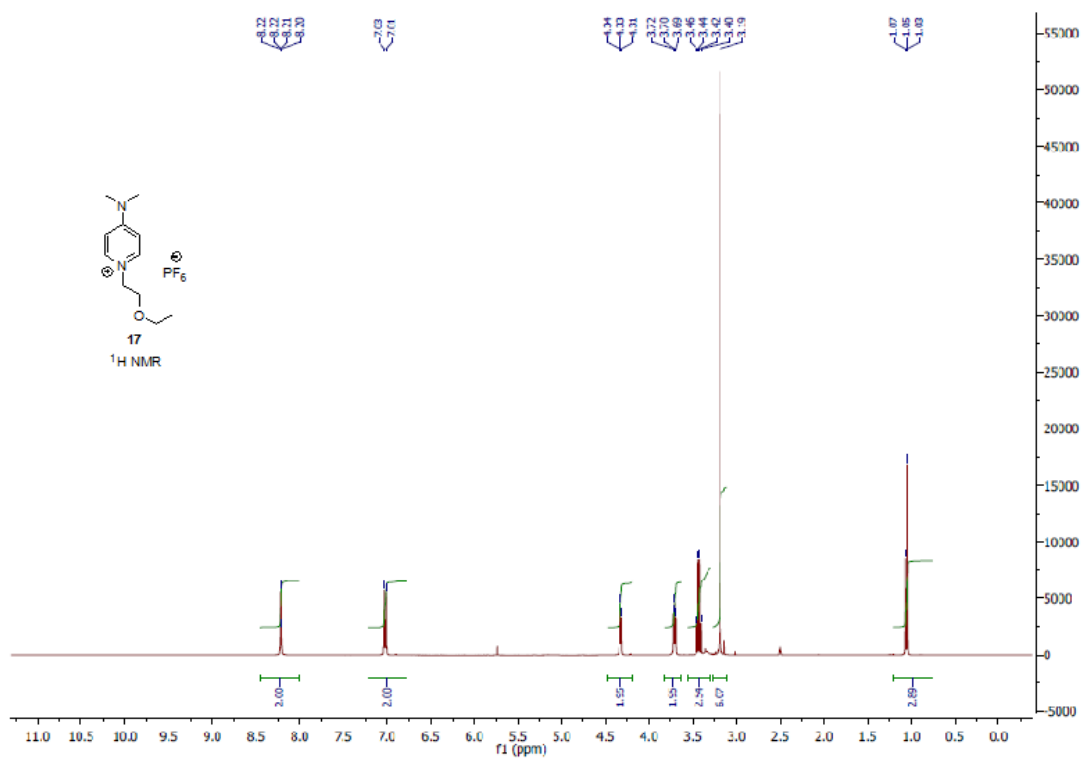

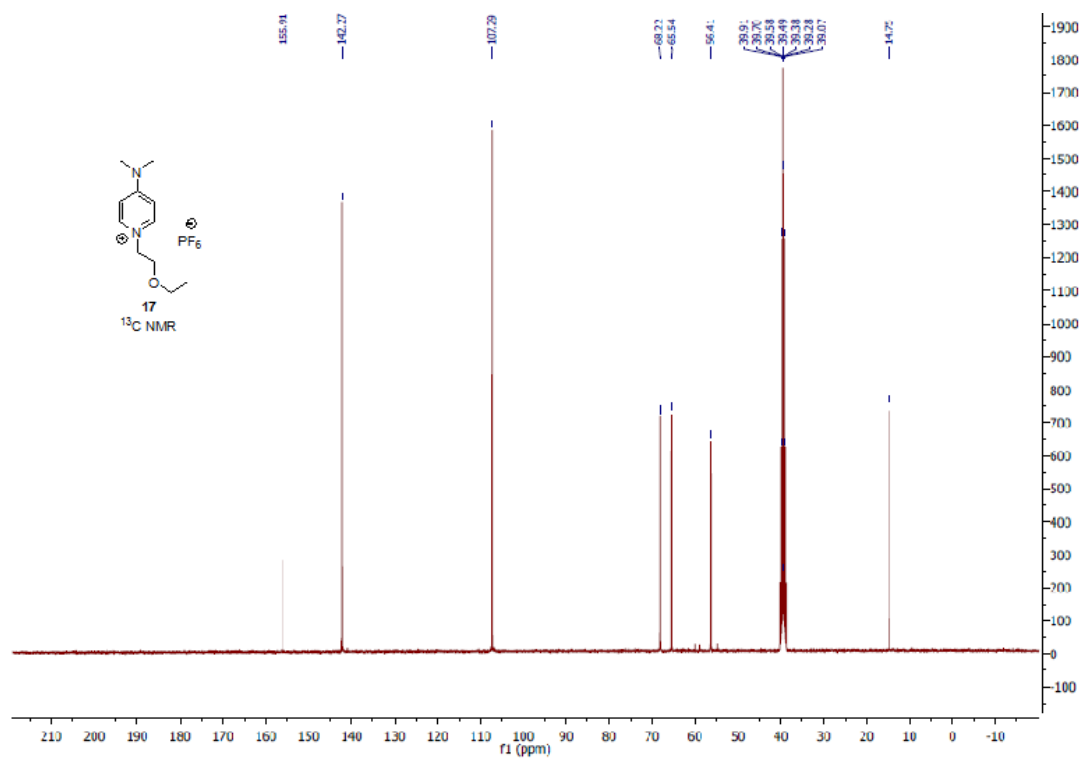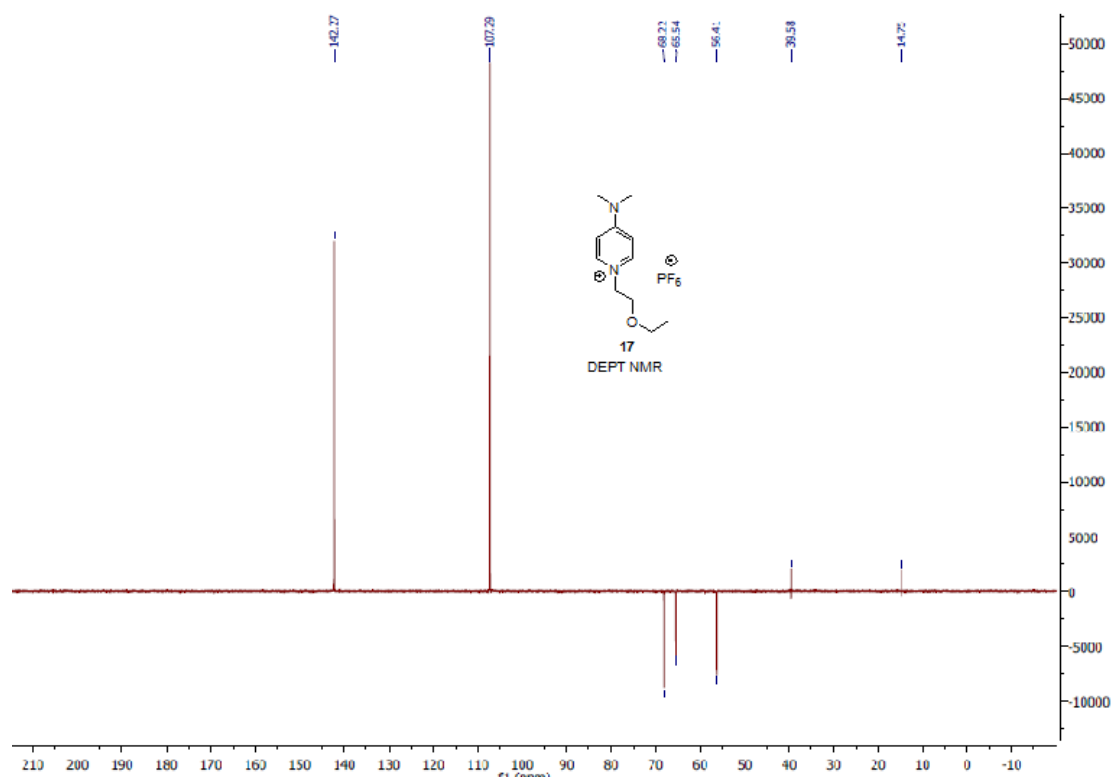

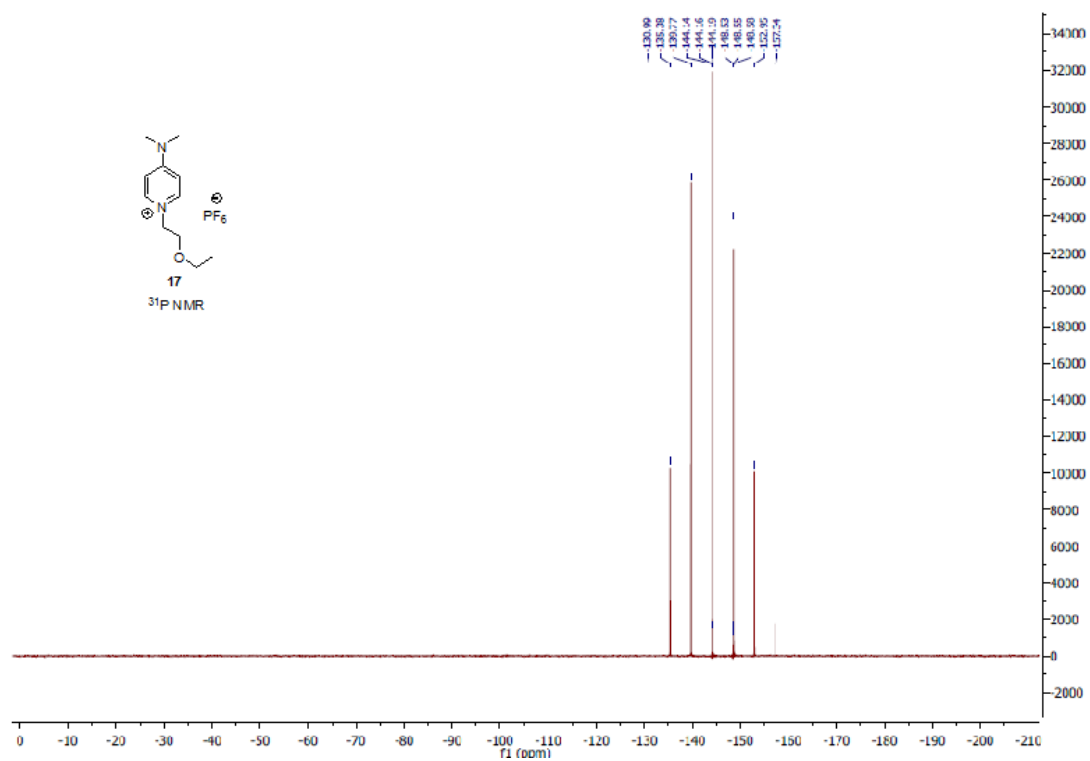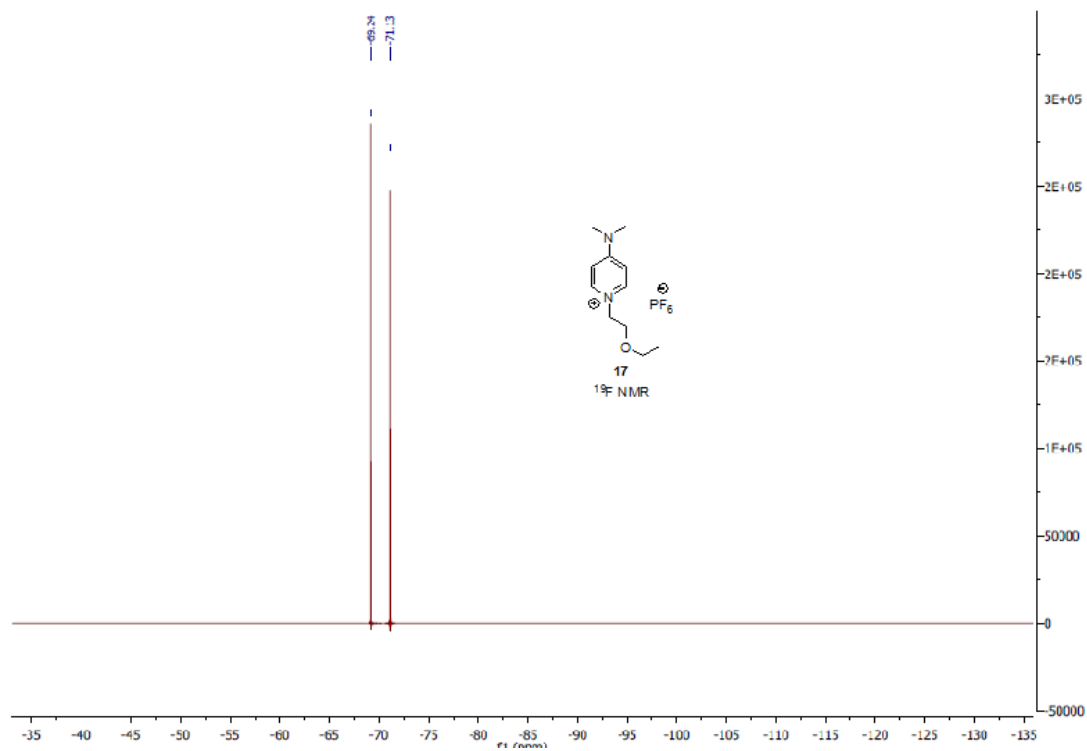

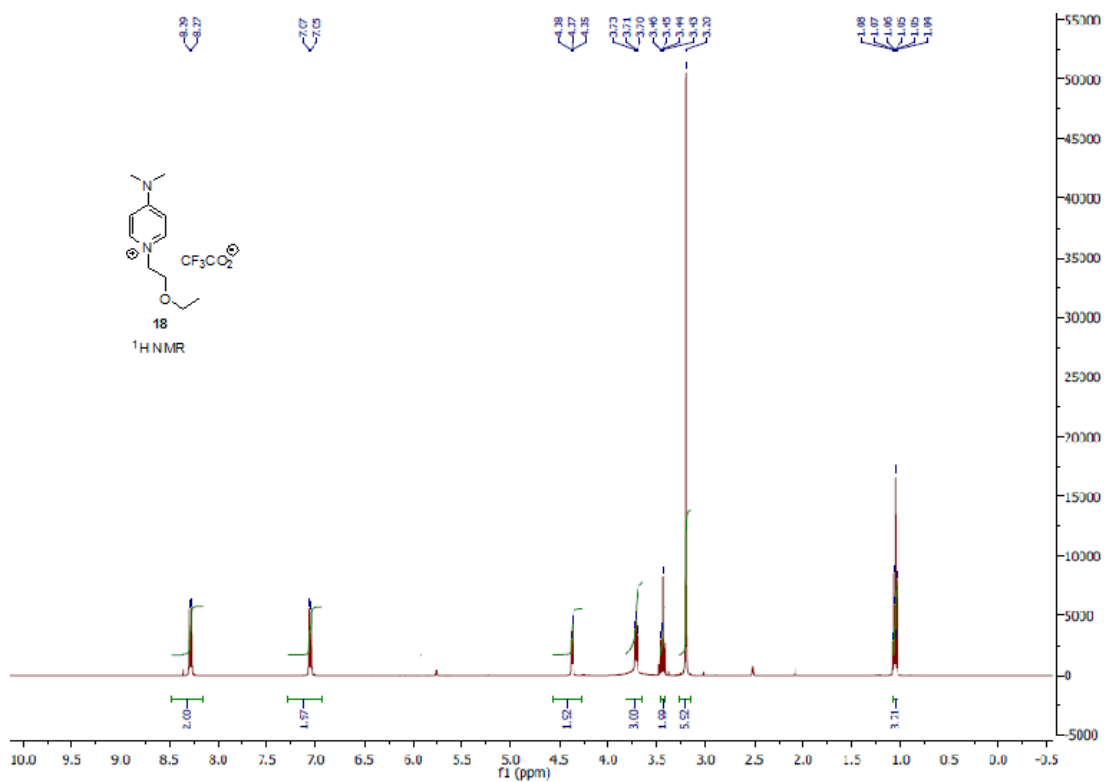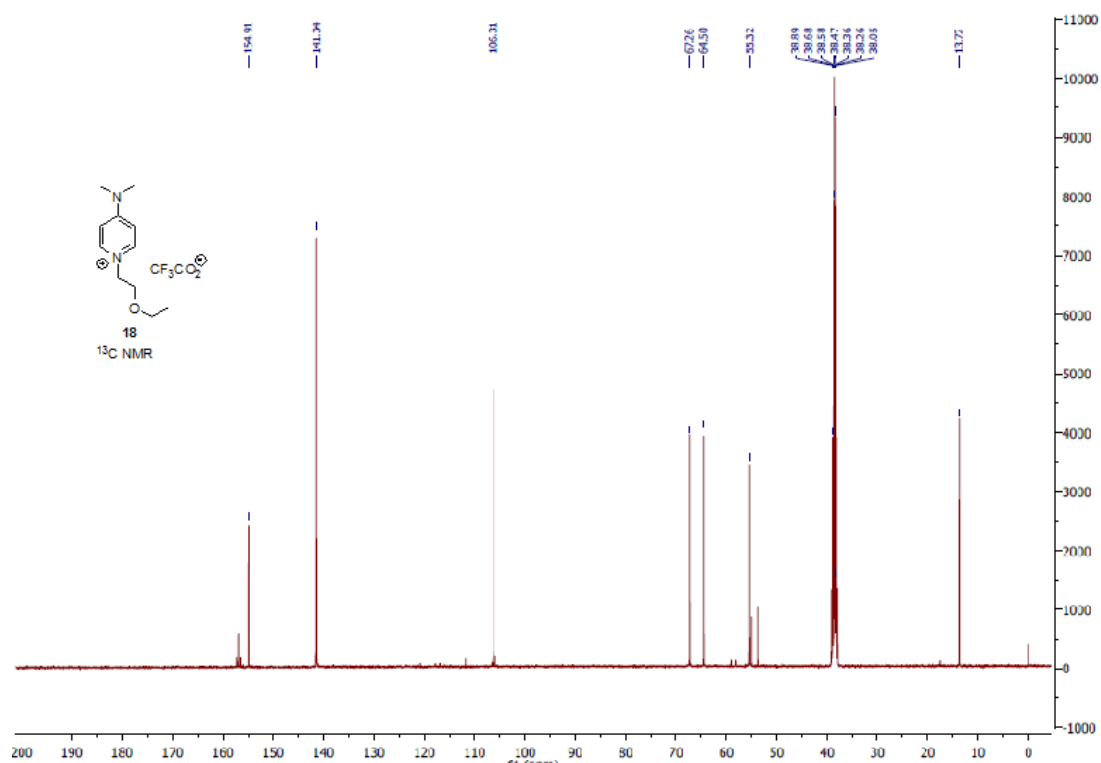

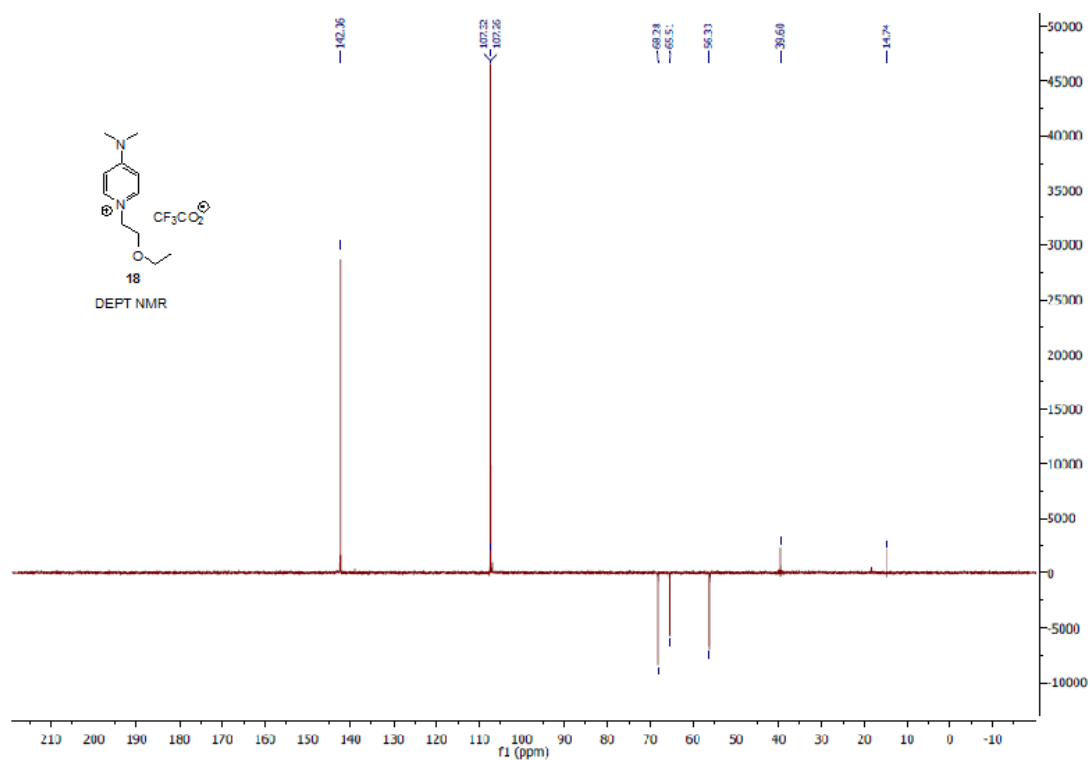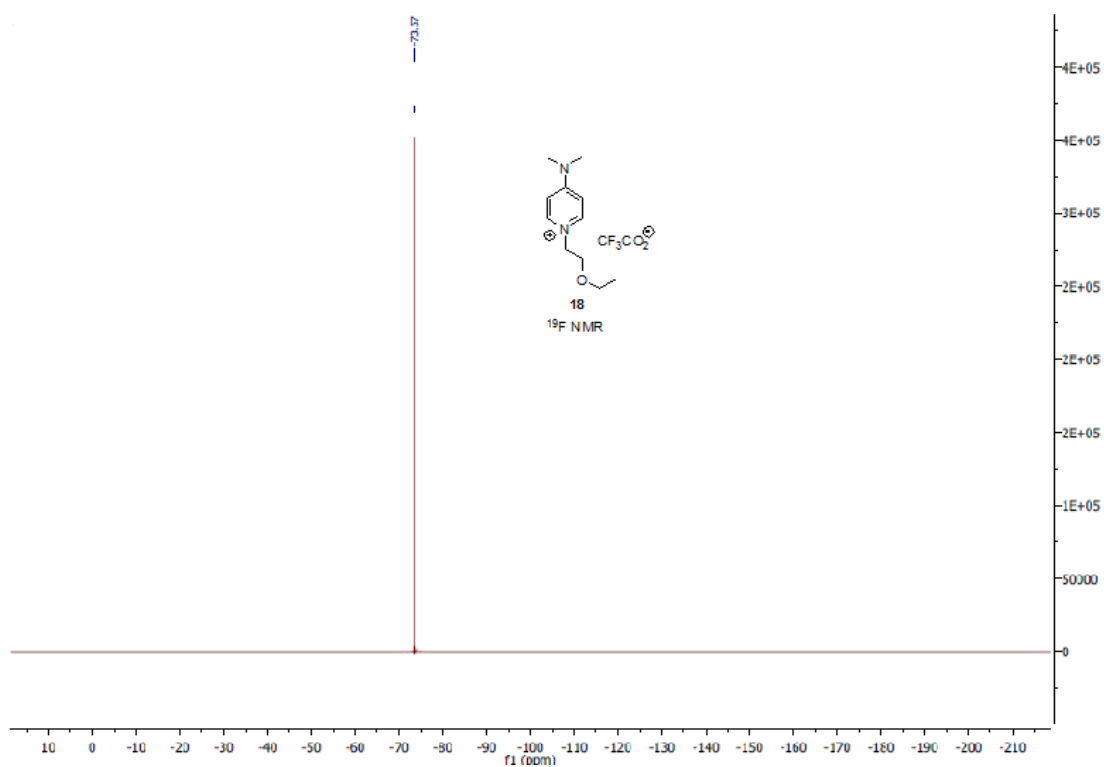

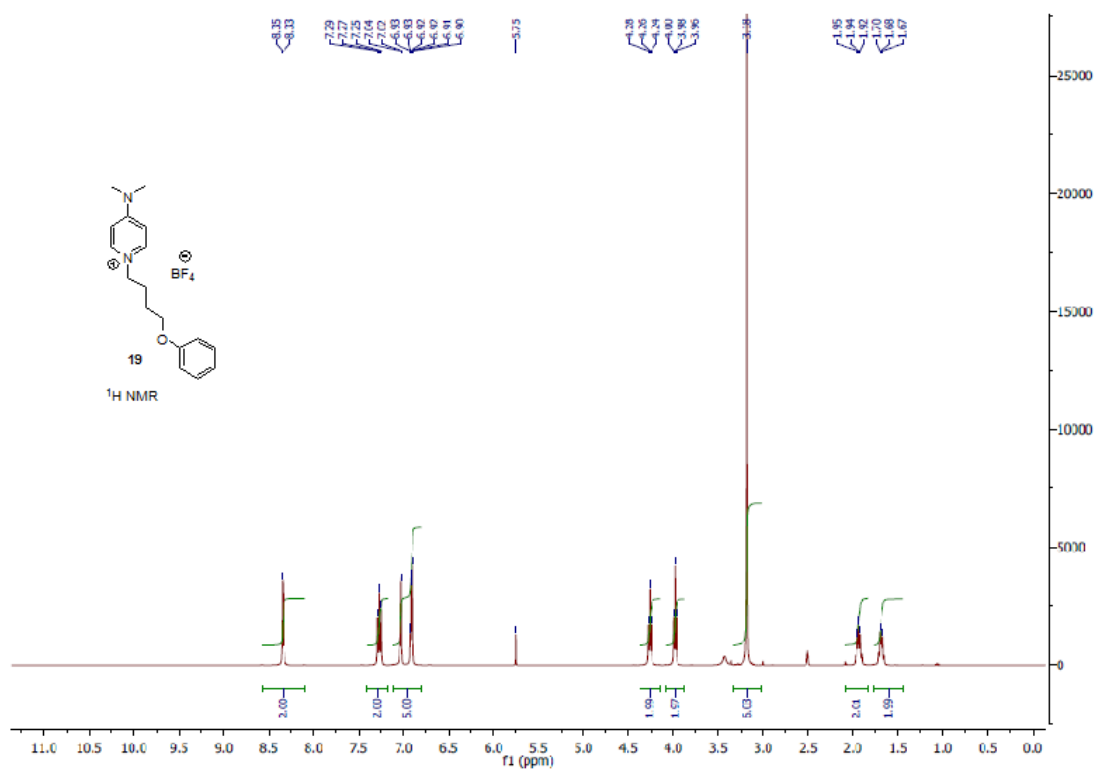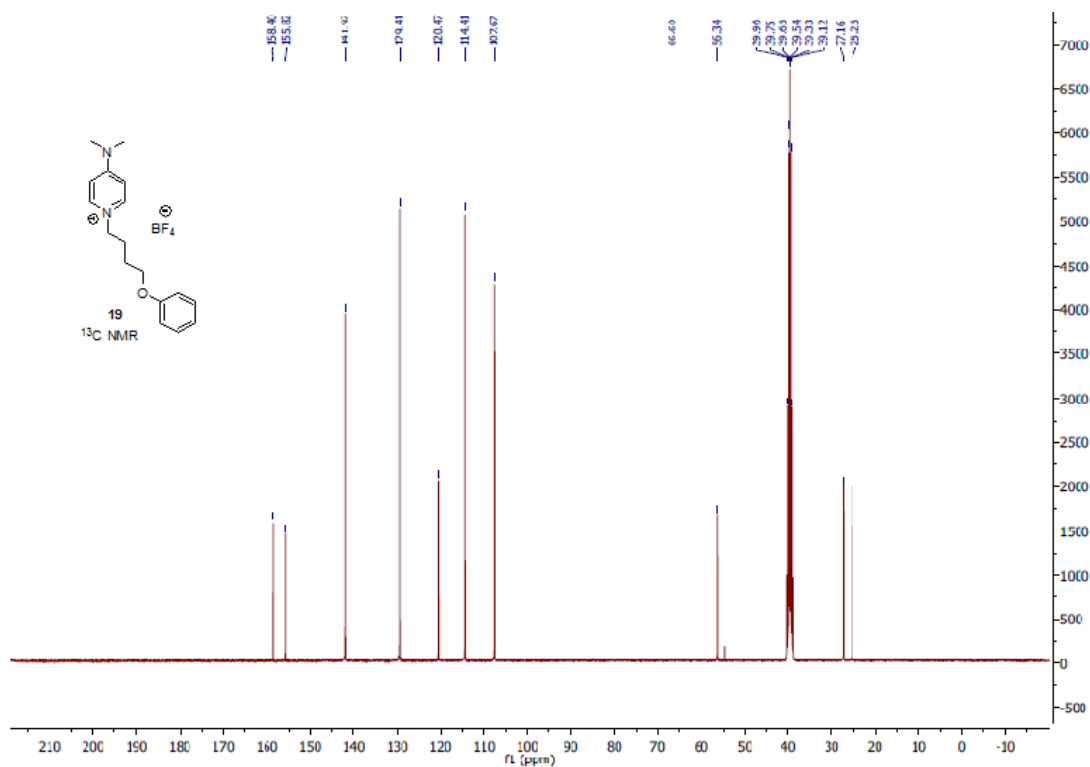

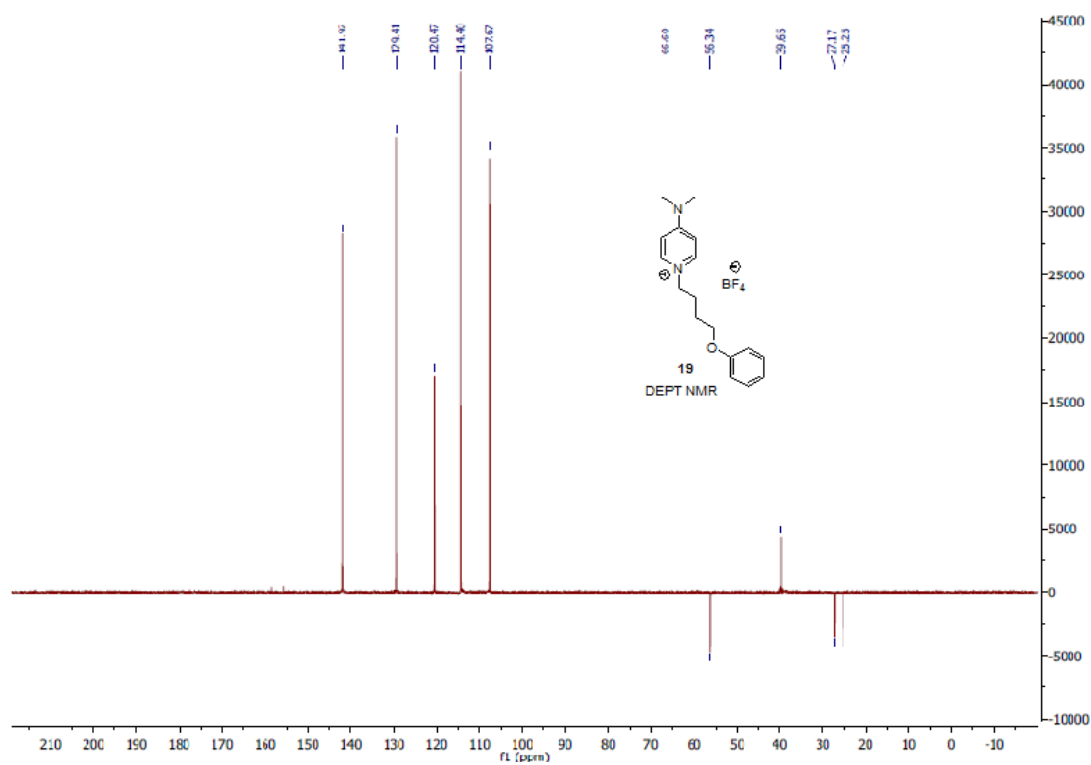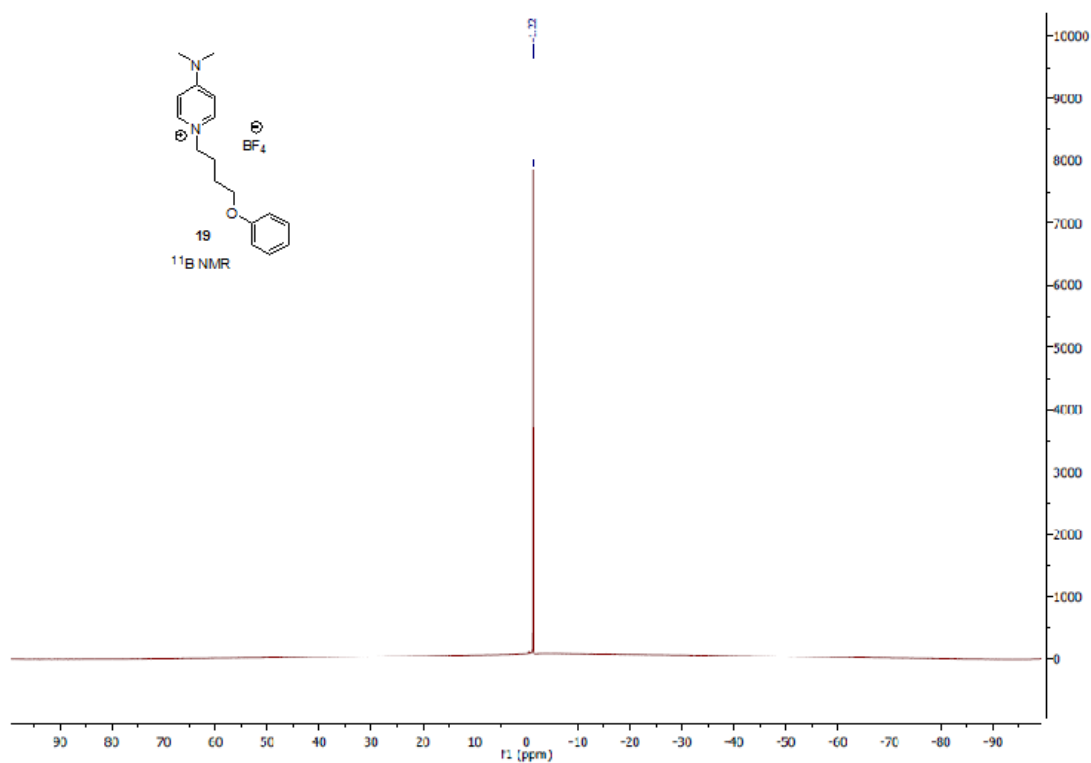

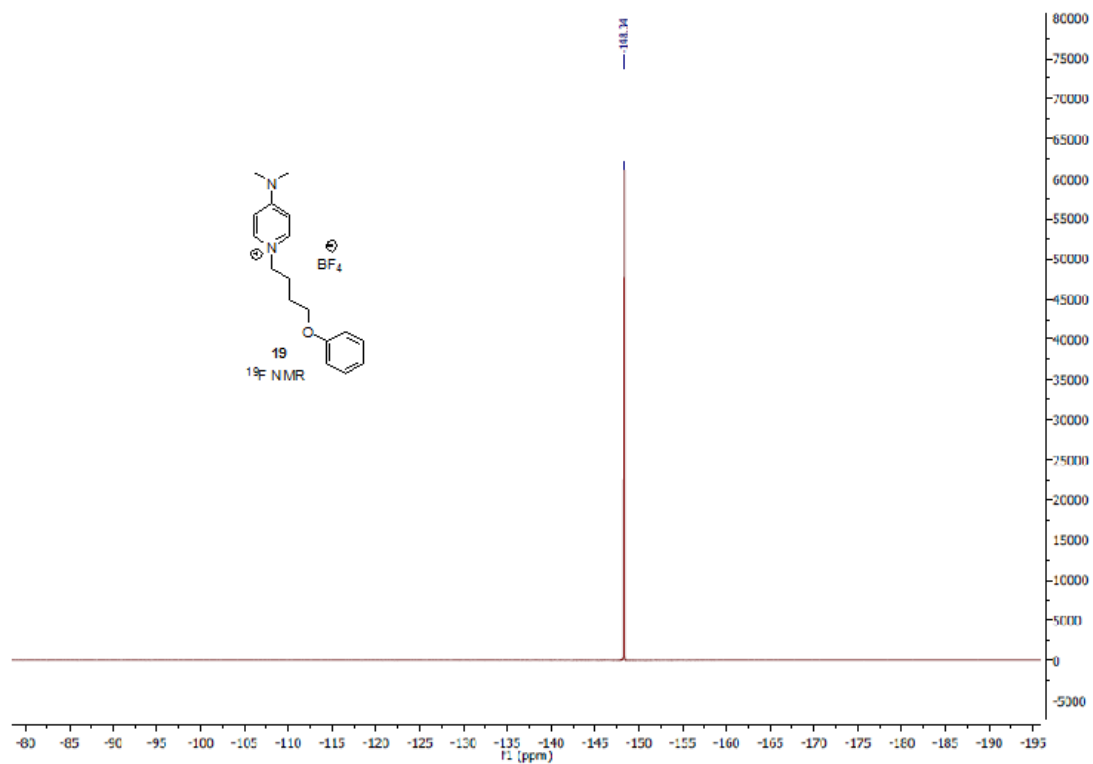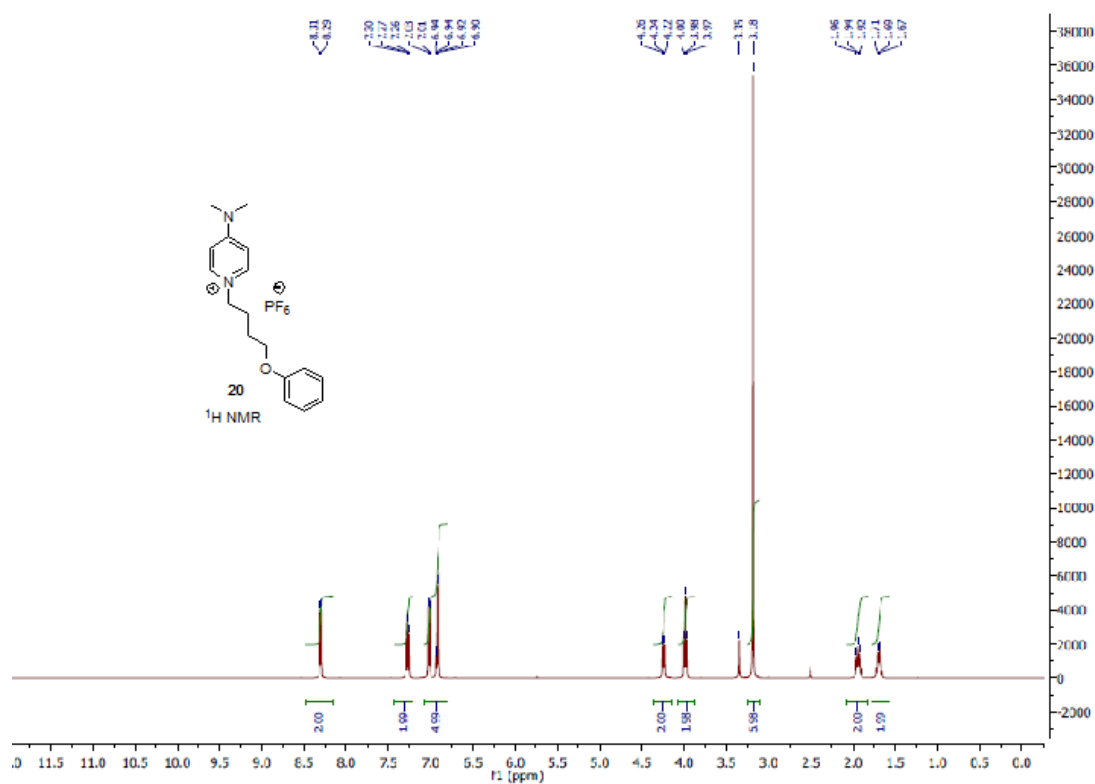

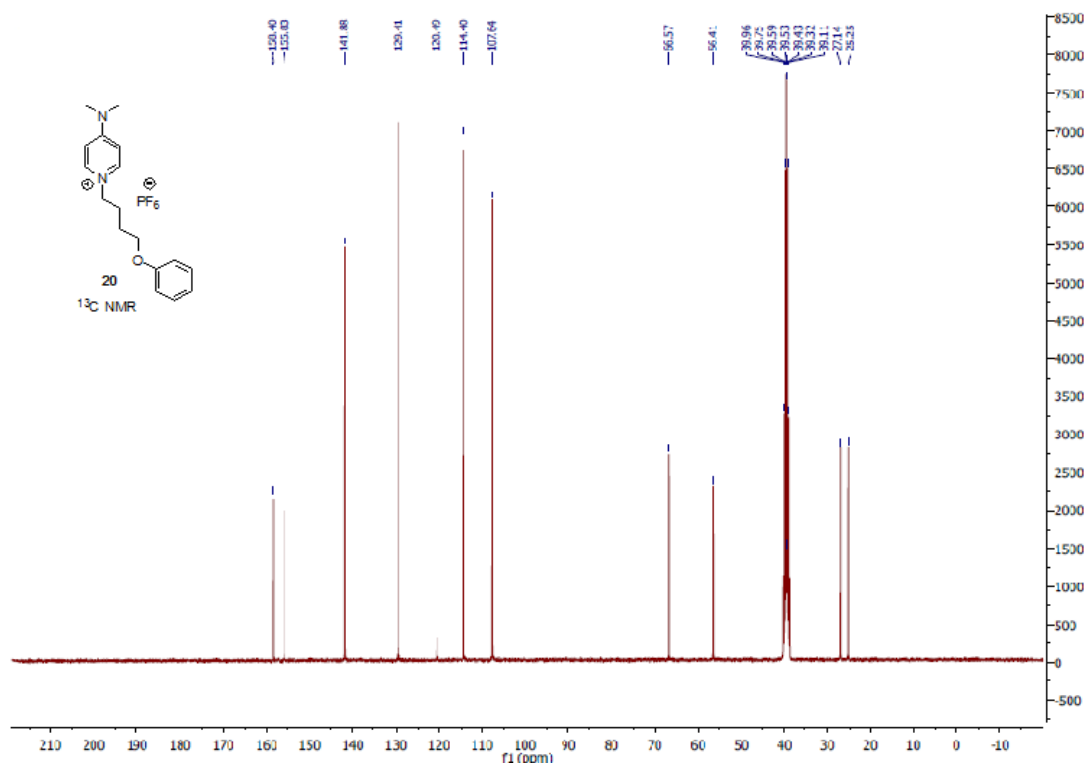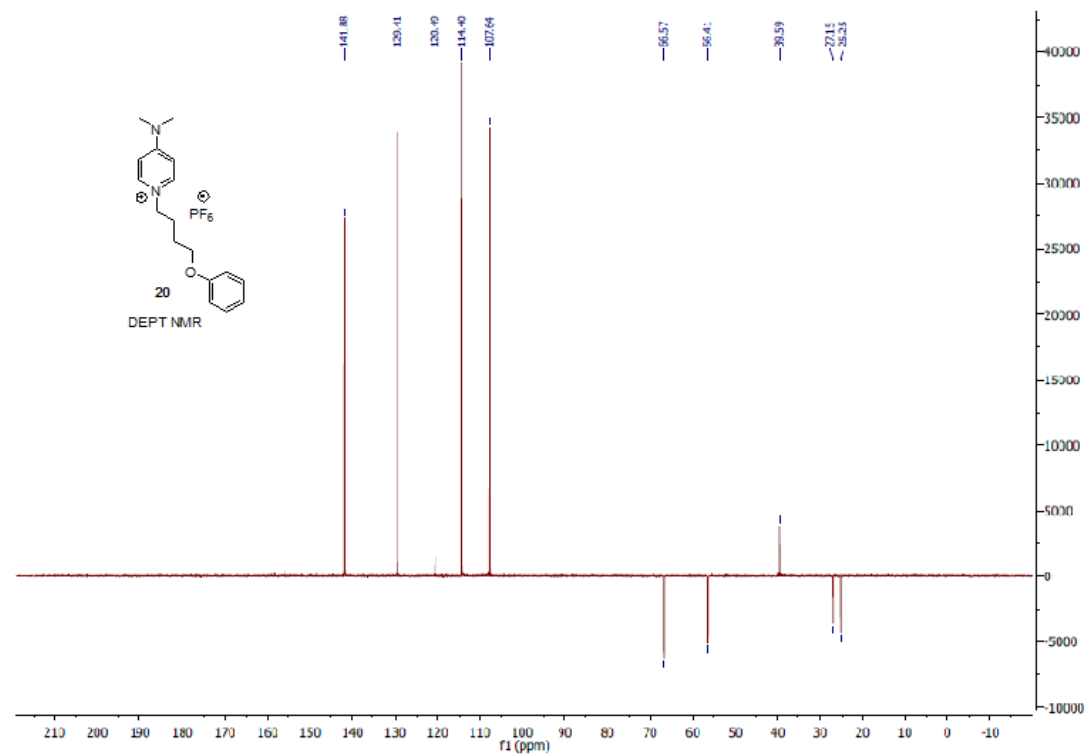

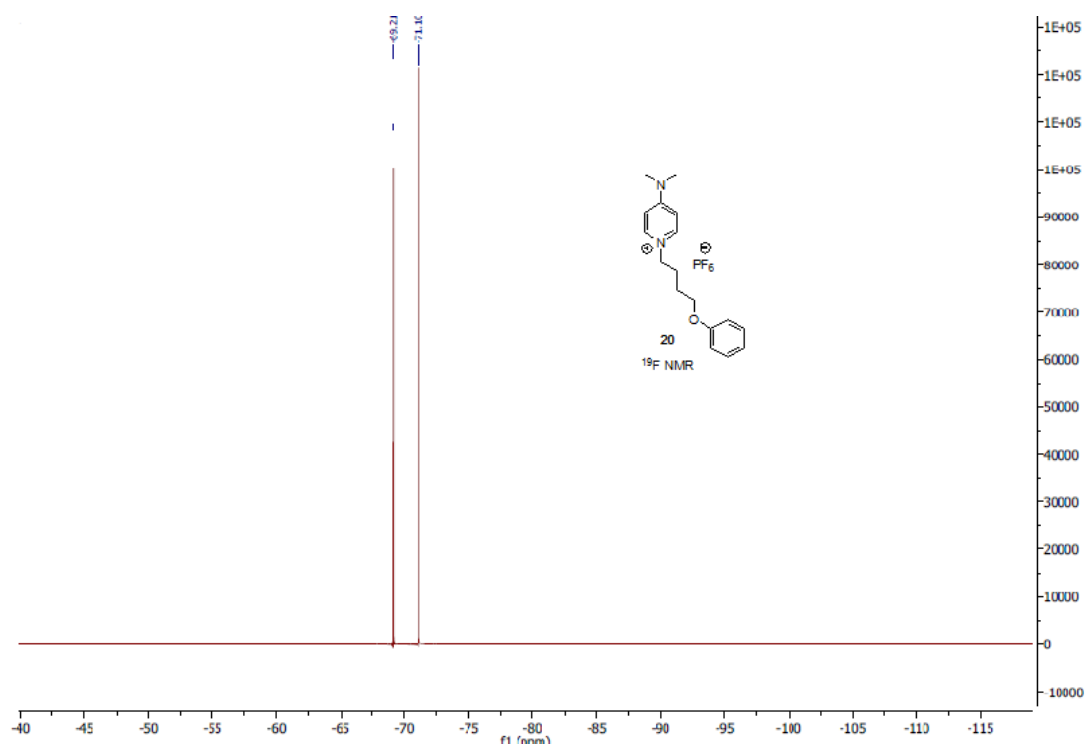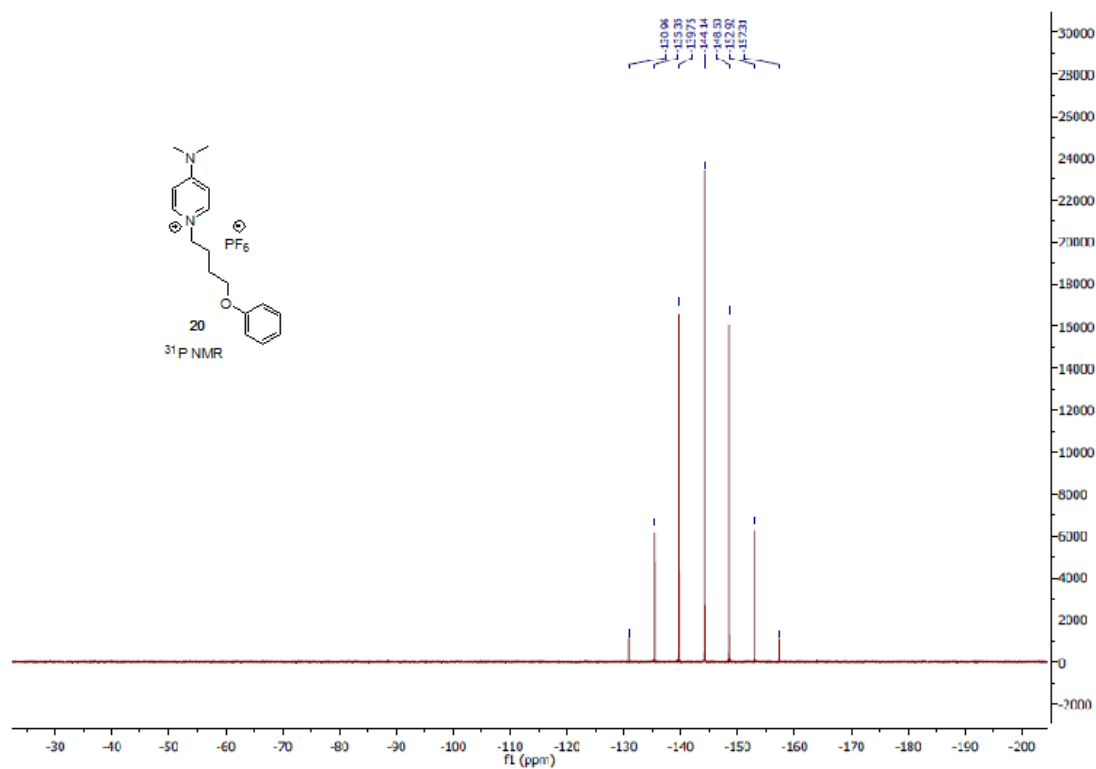

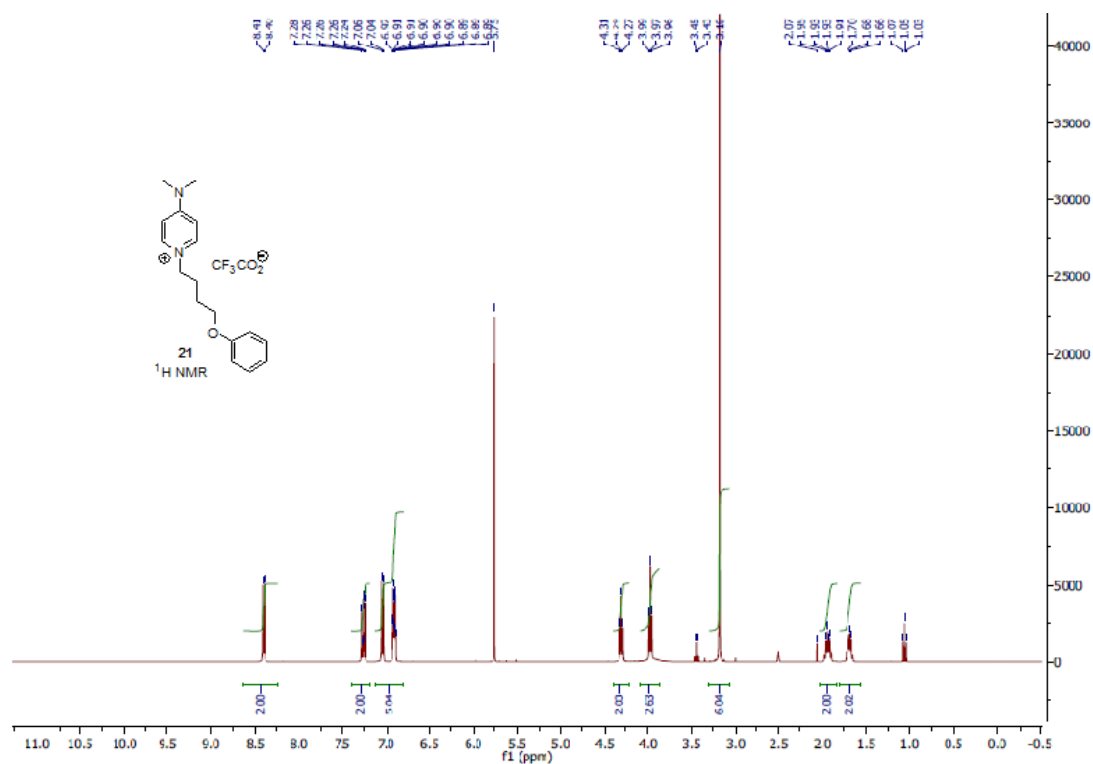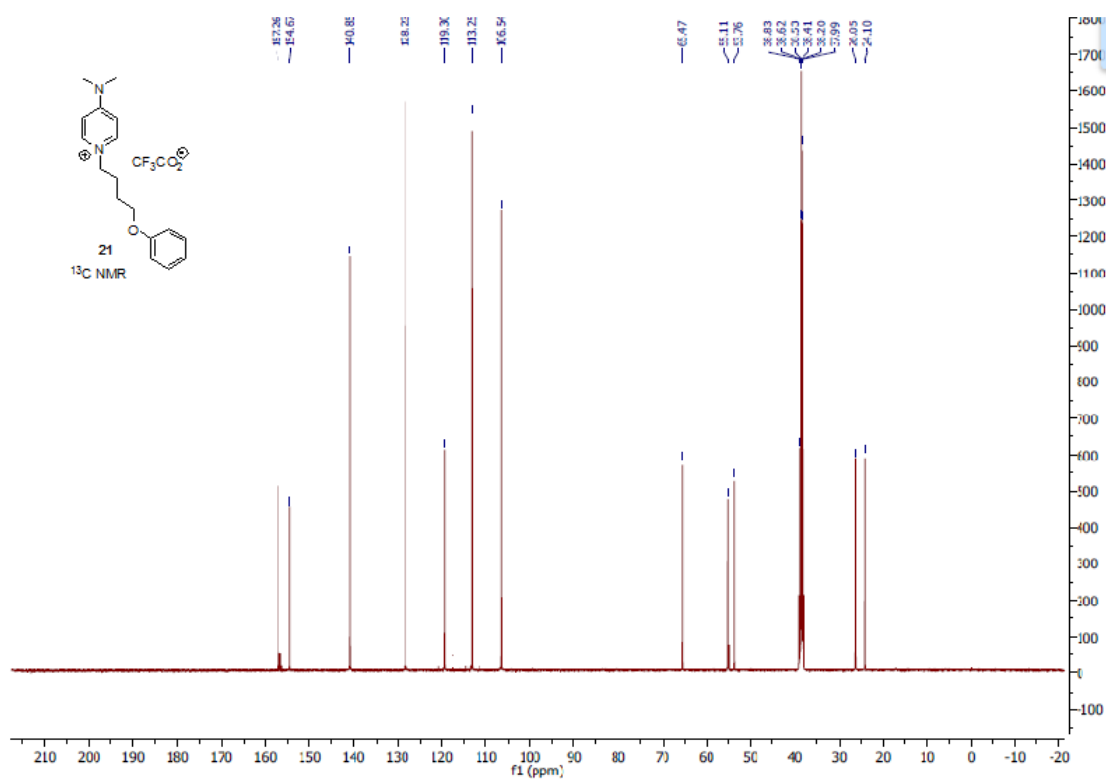

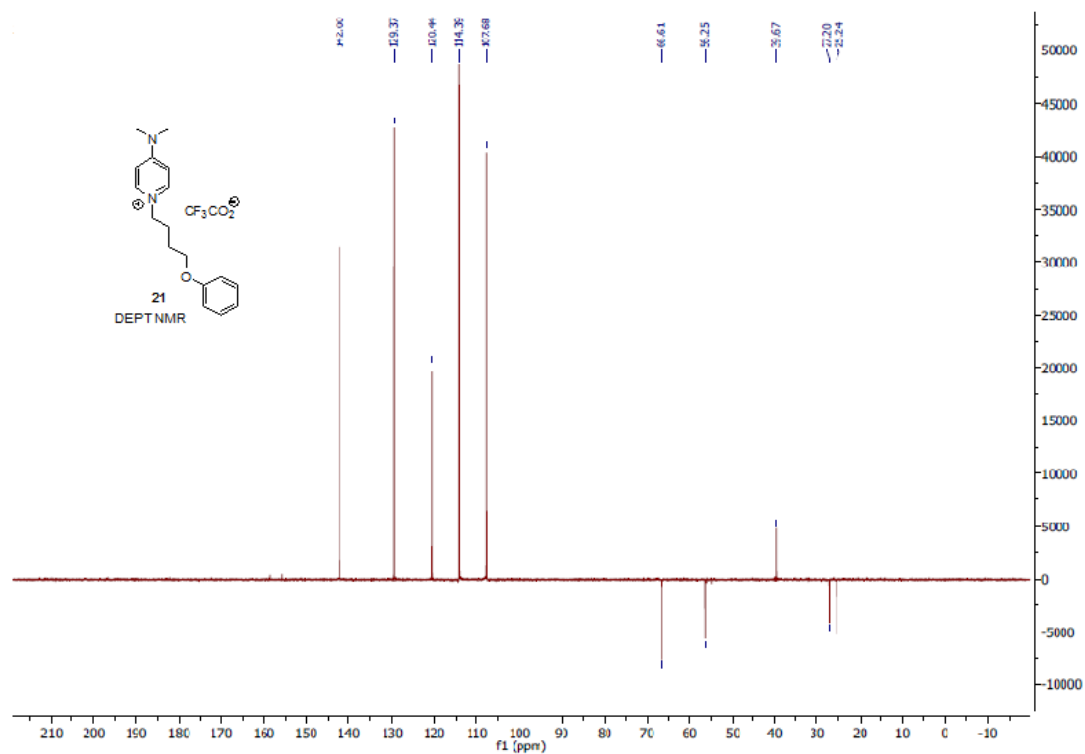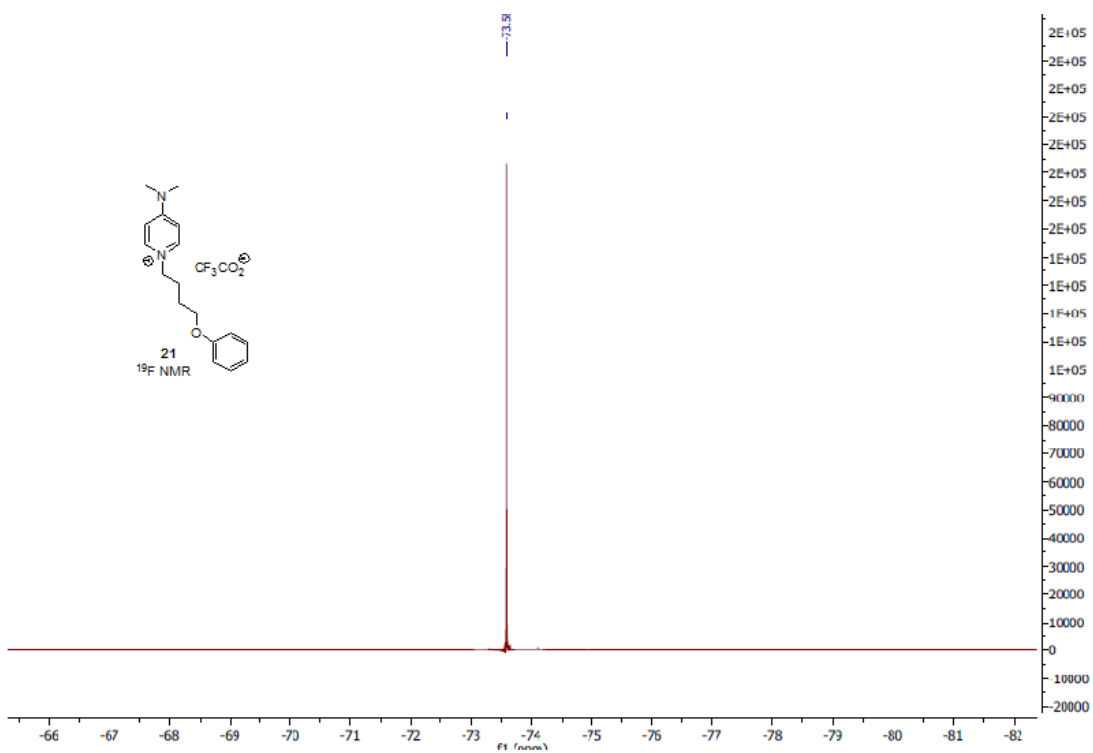

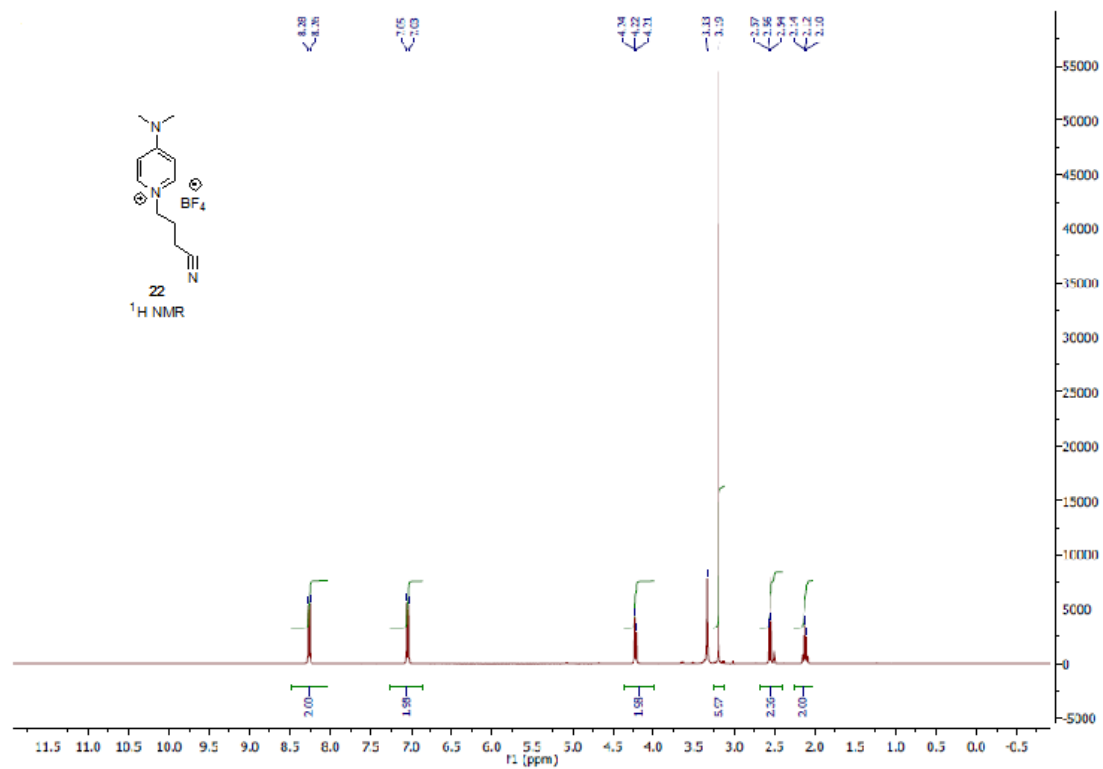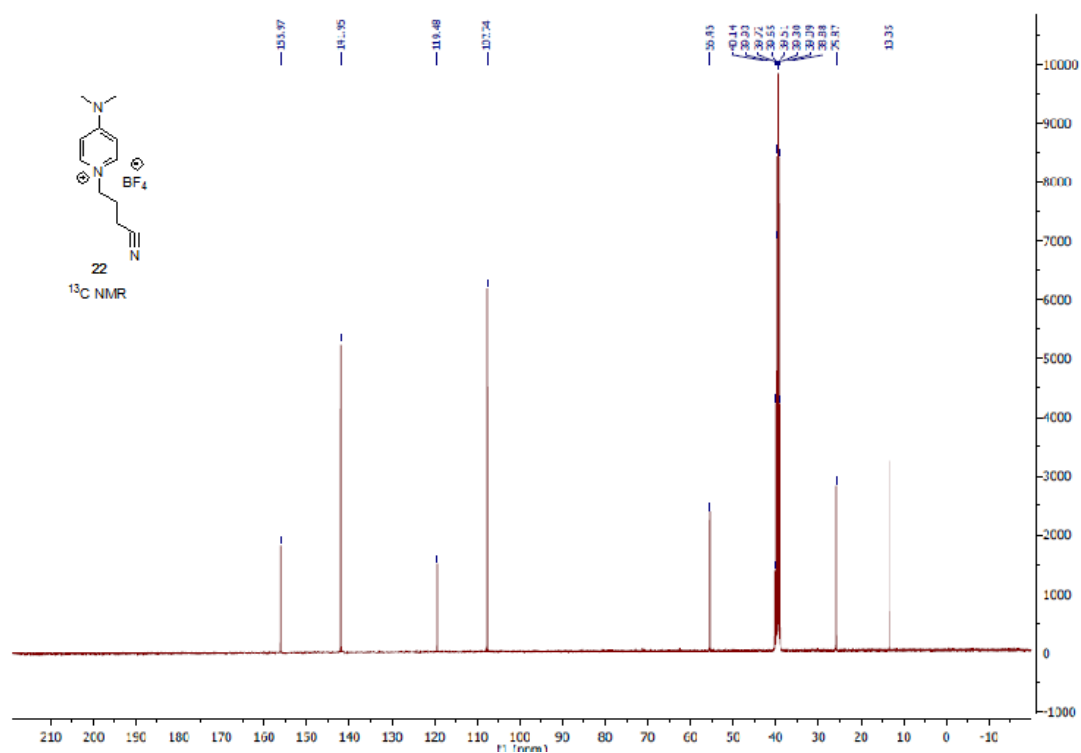

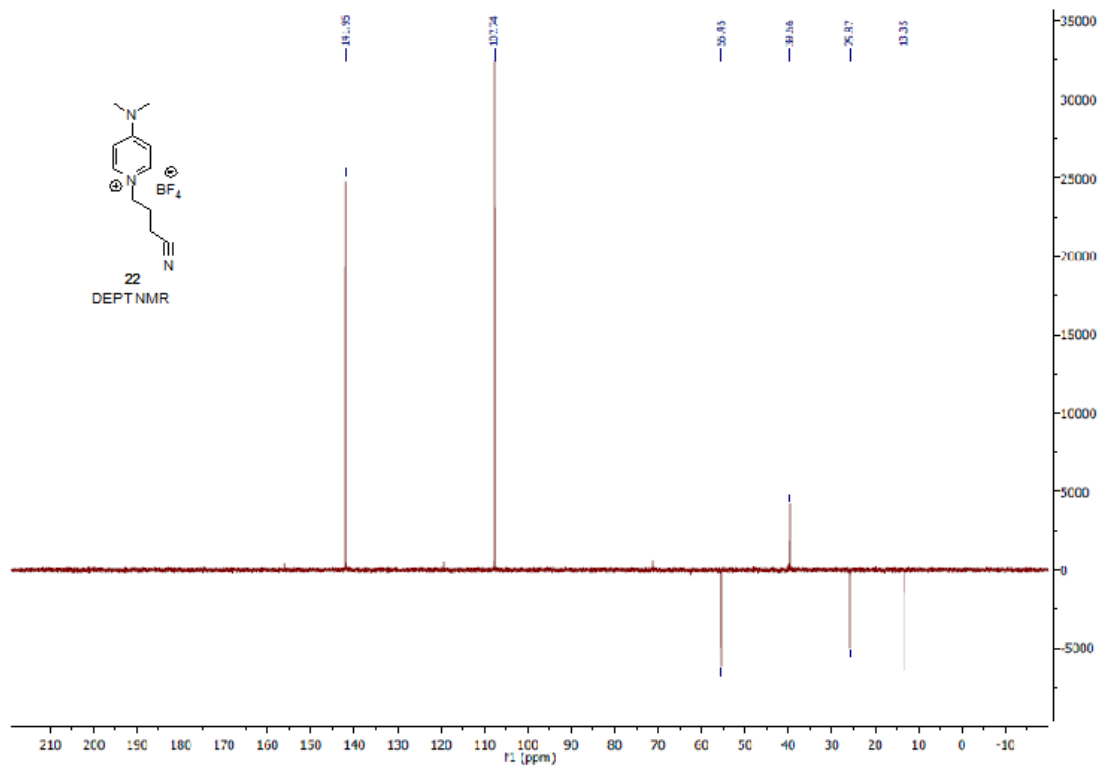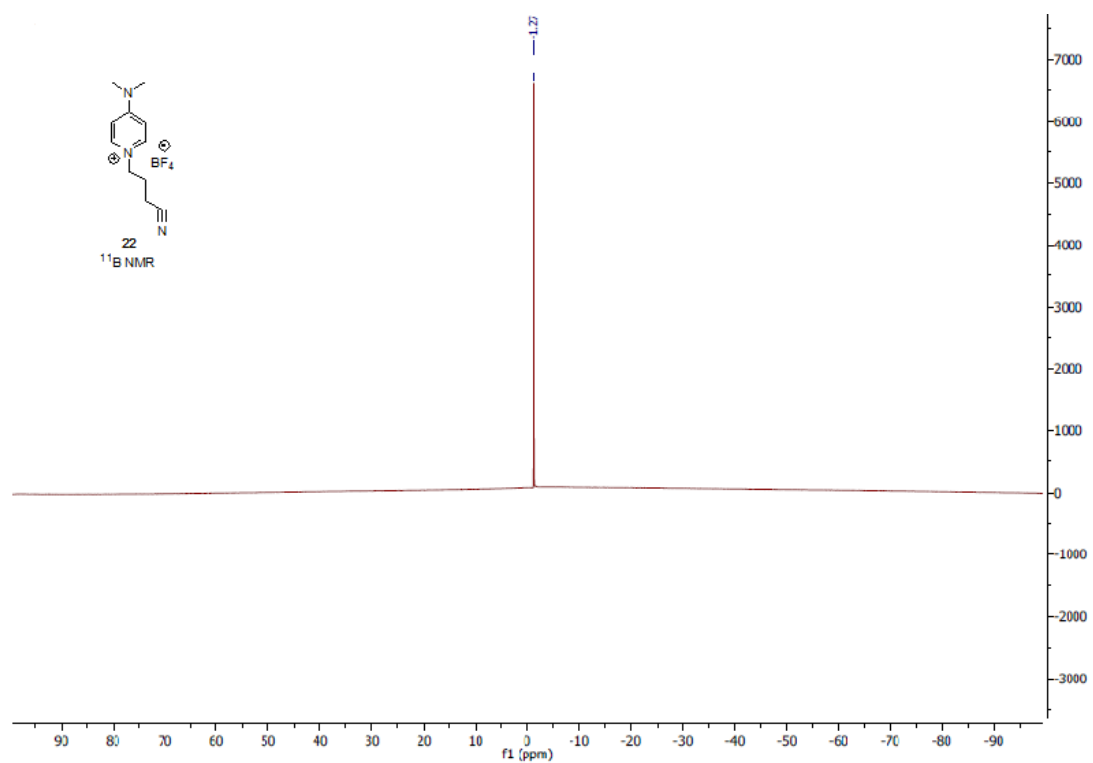

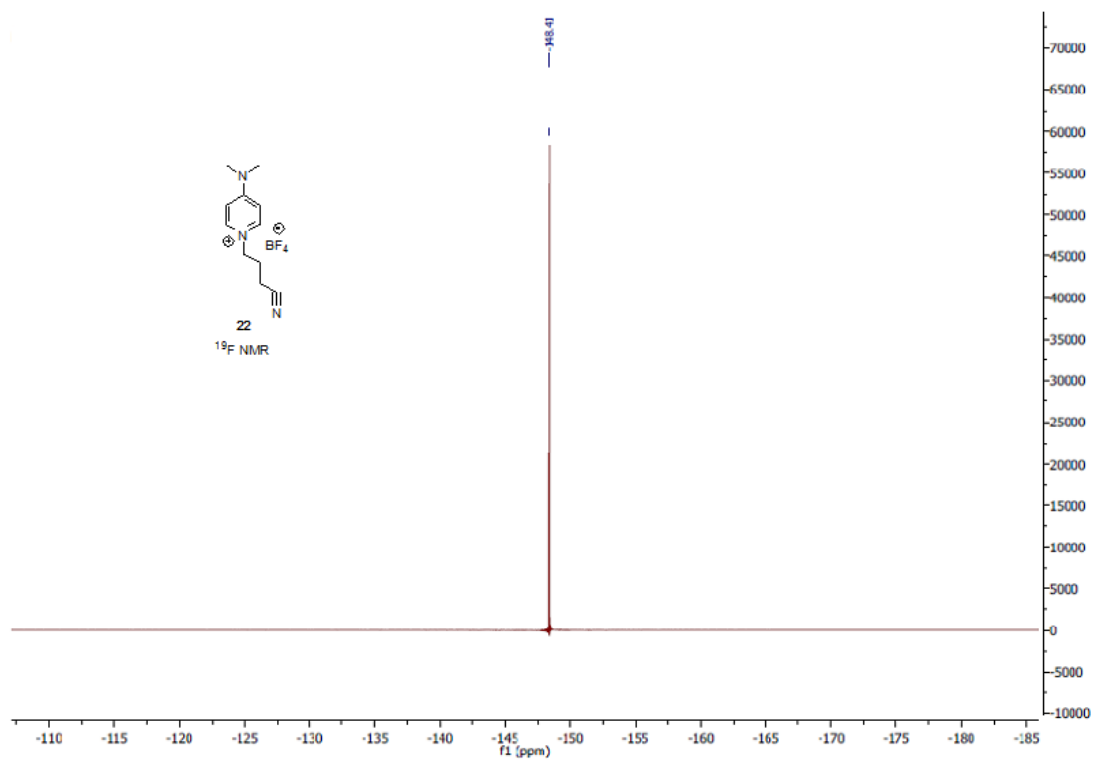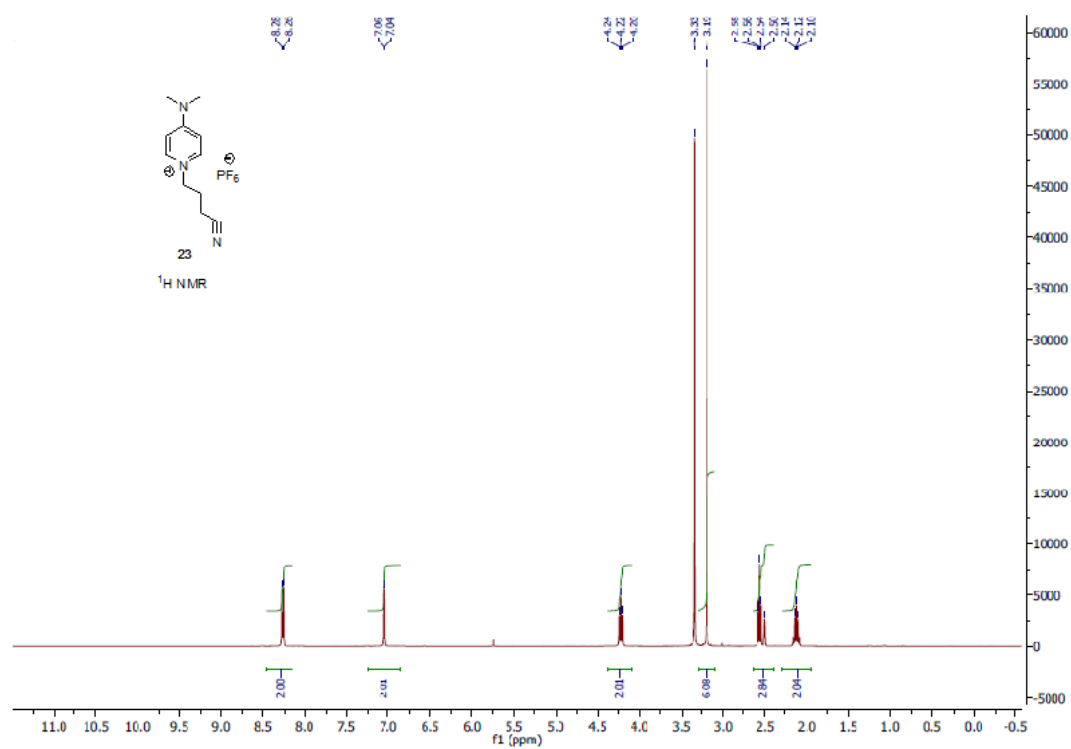

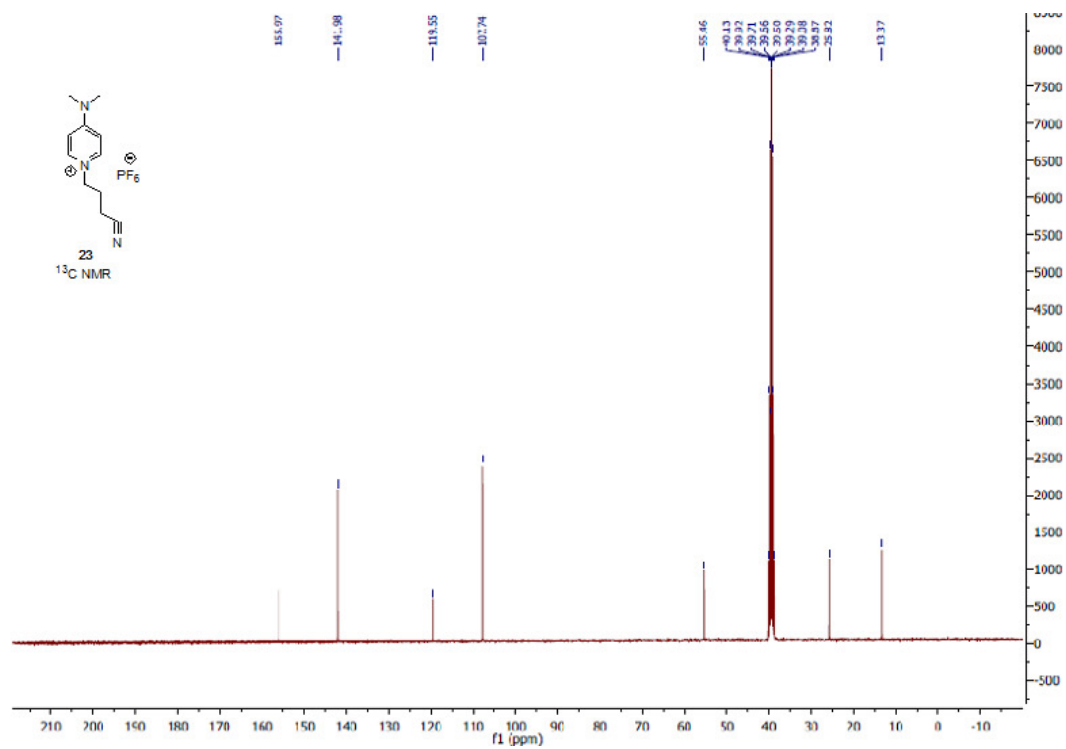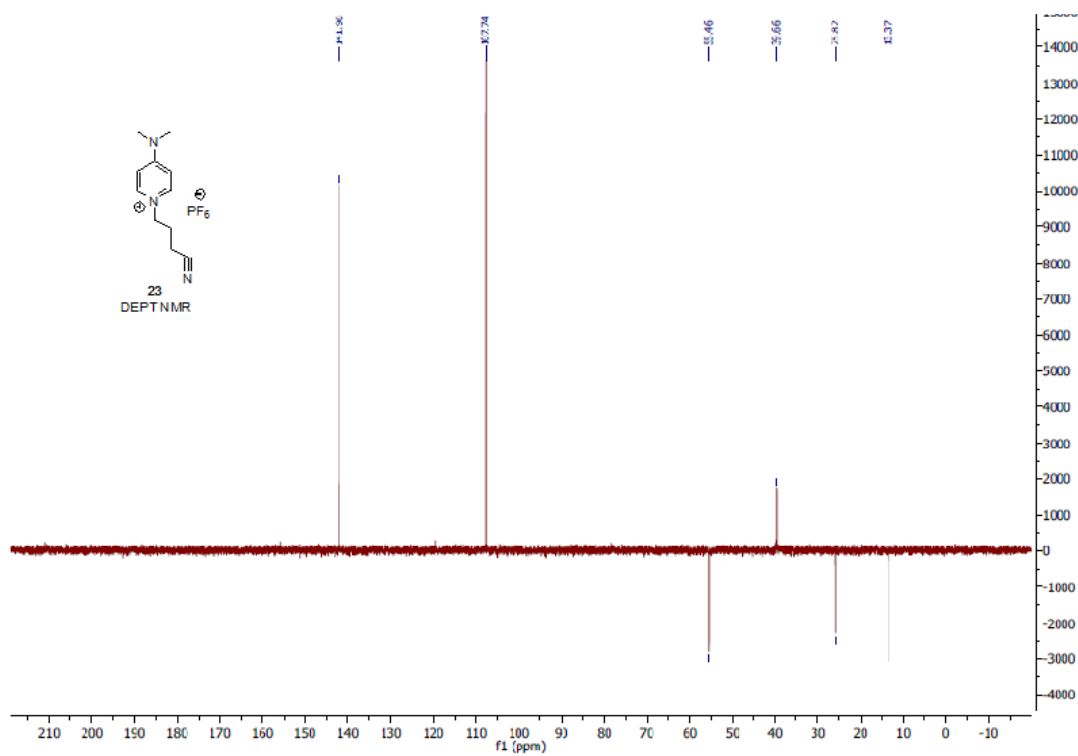

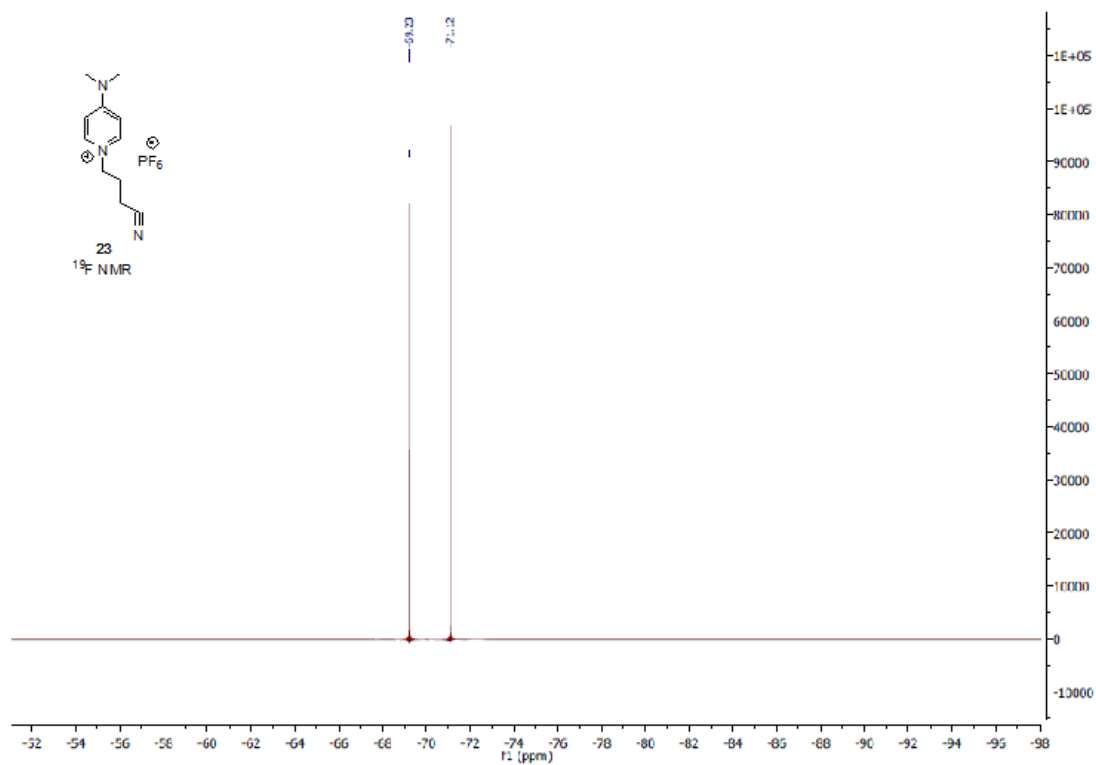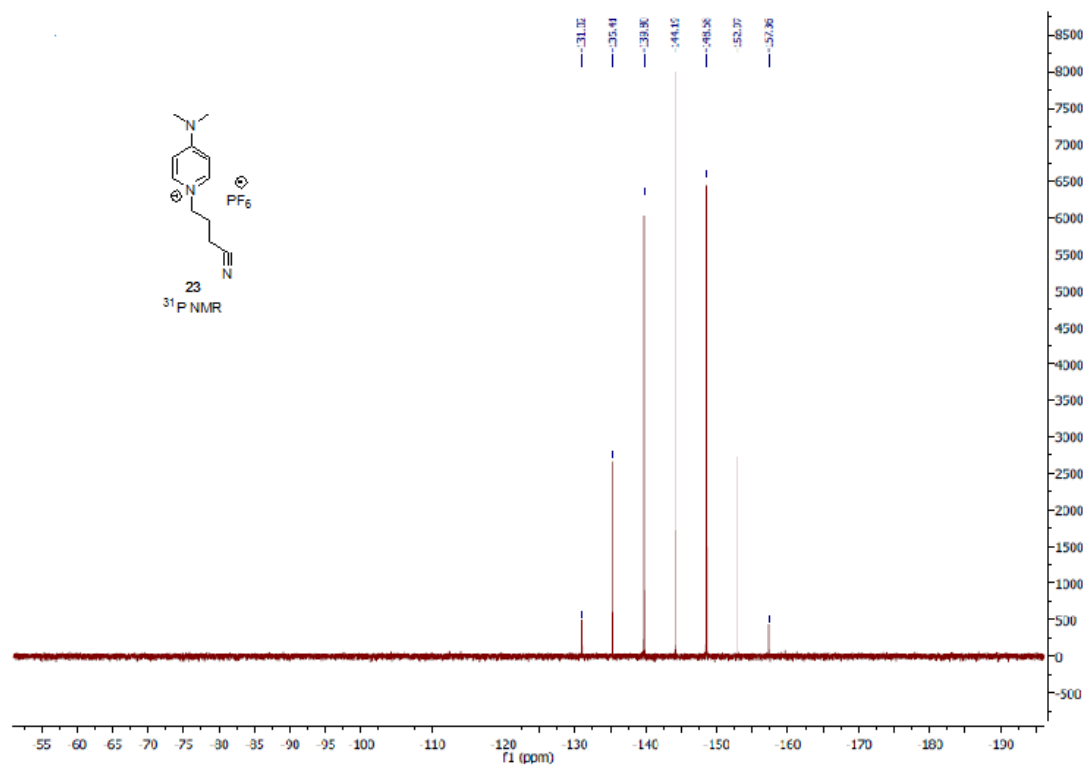

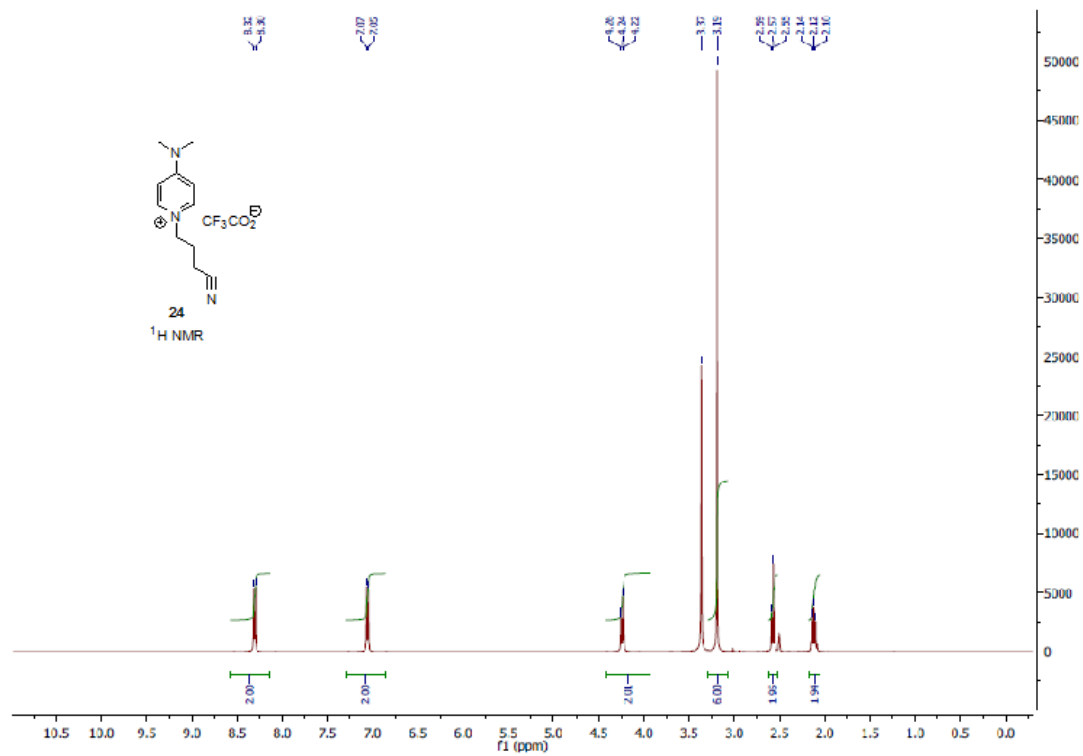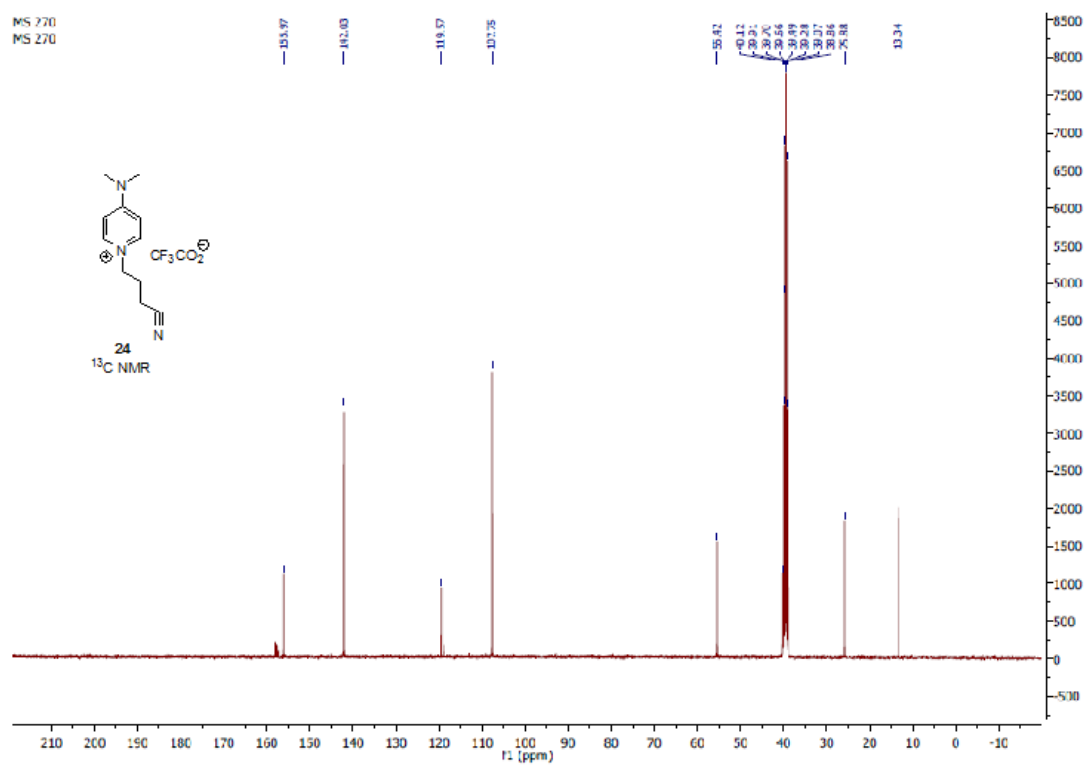

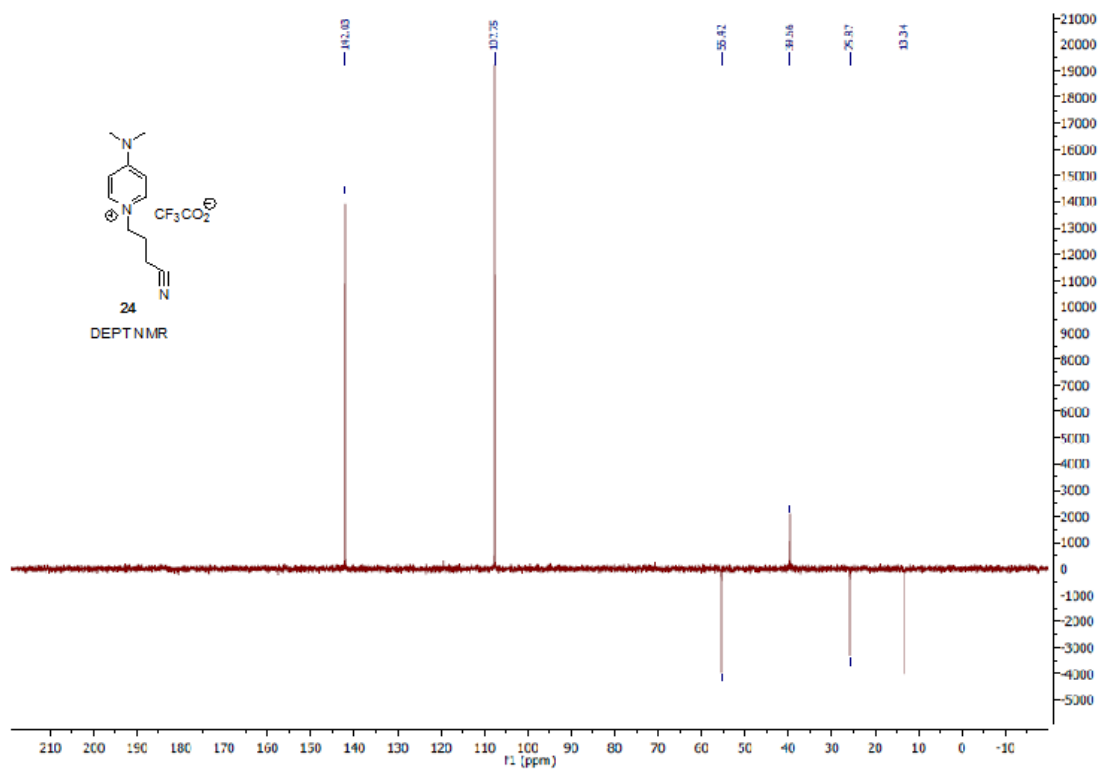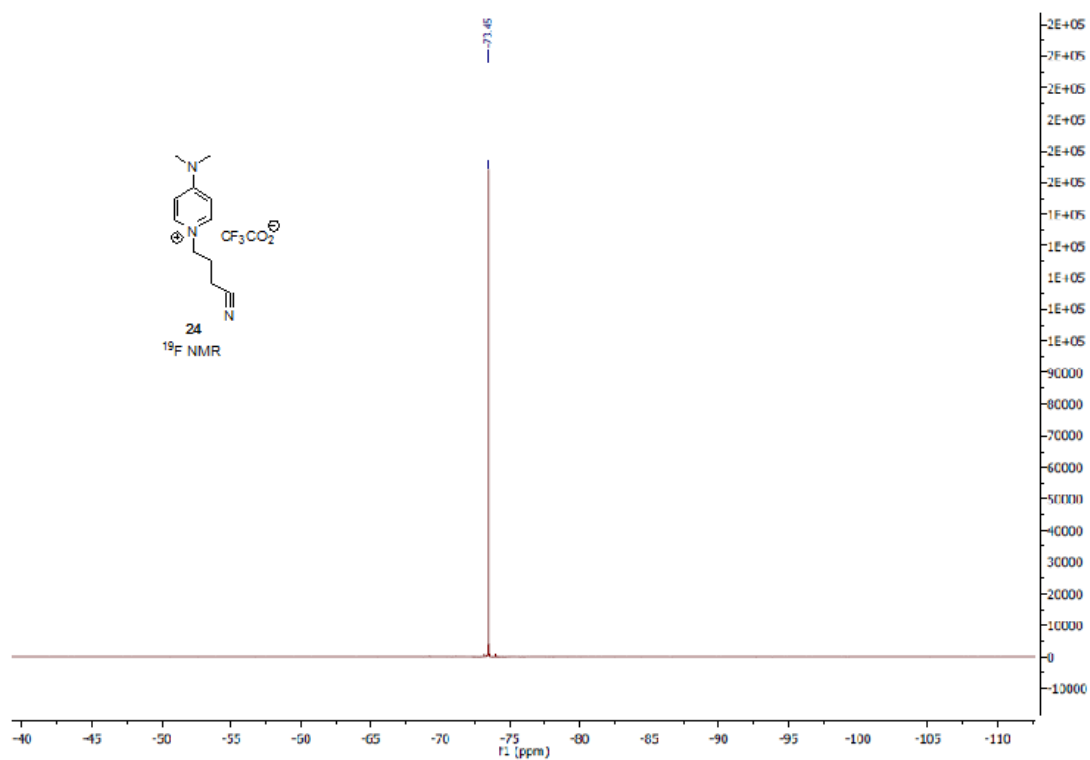

Supplement: Supplementary file 1 [file molecules-20-14936-s001.pdf]
